# Design and Synthesis of Novel Candidate CK1δ Proteolysis Targeting Chimeras (PROTACs)

**DOI:** 10.3390/molecules30224452

**Published:** 2025-11-18

**Authors:** Malte Arnold, Temi Thompson, Lorraine Glennie, Mattes Hollnagel, Gopal Sapkota, Christian Peifer

**Affiliations:** 1 Institute of Pharmacy, Department of Pharmaceutical and Medicinal Chemistry, Christian-Albrechts-University of Kiel, 24118 Kiel, Germany; marnold@pharmazie.uni-kiel.de (M.A.);; 2 MRC Protein Phosphorylation and Ubiquitylation Unit, Sir James Black Centre, School of Life Sciences, University of Dundee, Dundee DD1 5EH, Scotland, UK

**Keywords:** PROTACs, protein degradation, CK1δ, kinases, cancer therapy

## Abstract

The dysregulation of CK1 isoforms is linked to various types of diseases, including neurodegeneration and different types of neoplasia such as colon, pancreatic, breast, and ovarian cancer. For CK1 isoforms, a plethora of effective small molecule inhibitors are available. However, only a few degraders of CK1α and, more recently, proteolysis targeting chimeras (PROTACs) for CK1δ/CK1ε have been reported. In this study, we applied the PROTAC concept by harnessing molecular modelling to design and synthesize a series of candidate CK1δ-targeting PROTACs based on a highly specific and potent benzothiazole-based CK1δ inhibitor that we previously developed in our lab. In the present study, we established a modular synthetic platform to systematically generate a set of PROTAC degrader candidates consisting of the CK1δ-specific inhibitor scaffold, alkyl and PEG linker motifs with various lengths, and Cereblon (CRBN)-engaging pomalidomide and thalidomide derivatives as E3 ligase binders. We demonstrate that several PROTACs degrade CK1δ/ε in various cells. The most potent PROTAC **P1d** inhibits the phosphorylation of downstream substrates through CK1δ/ε degradation. We establish the requirement of CUL4A^CRBN^ and the proteasome for the **P1d**-mediated degradation of CK1δ/ε.

## 1. Introduction

**Figure 1:**
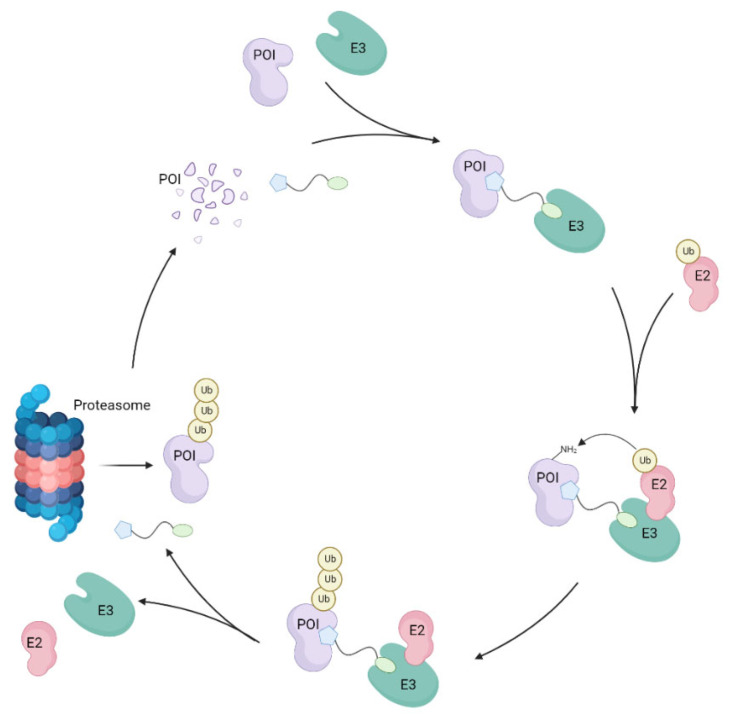
A schematic representation of the mechanism of action of PROTAC compounds. PROTACs hijack the UPS by forming ternary complexes with the POI and an E3 ligase, therefore artificially inducing proximity between these proteins [11,12]. The latter then recruits an E2 ligase, which transfers its ubiquitin molecules onto the POI. The polyubiquitinated POI is then marked for degradation by the 26S proteasome. Due to its catalytic nature, PROTACs can undergo this cycle of inducing the polyubiquitination and degradation of a targeted POI in sub-stoichiometric amounts [13,14,15]. Figure created with BioRender.com.

The concept of proteolysis targeting chimeras (PROTACs) represents contemporary and innovative therapeutic modalities, first established by Crews and colleagues in 2001 [1]. PROTACs’ catalytic mode of action (MOA) potentially offers several advantages over small molecule inhibitors (SMIs), such as the accessibility of previously undruggable targets, enhanced protein isoform selectivity, and reduced dosing frequency [2]. PROTACs induce the degradation of a protein of interest (POI) by hijacking the intrinsic cellular ubiquitin–proteasome system (UPS), thereby triggering a conditional POI knockdown phenotype [1,3]. The UPS itself is an essential pathway in cells that physiologically regulates the life cycle of proteins. Hence, while regulating the degradation of damaged or misfolded proteins in general, it plays major roles, e.g., in the regulation of the cell cycle, as well as inflammatory and immune responses [4,5,6]. The dysregulation of the UPS is often associated with diseases, including different forms of cancer as well as neurodegenerative diseases such as Parkinson’s, Alzheimer’s, or Huntington’s disease [7,8,9]. Compared to SMIs, the alternative MOA of PROTACs correlates to their nature as heterobifunctional molecules. Typical PROTAC compounds consist of both an E3 ligase ligand and a POI ligand, which are connected via different types of linkers [10]. These chimeric molecules are therefore able to simultaneously recruit the respective E3 ligase and the POI, forming ternary complexes. Consecutively, due to the induced spatial proximity, the E3 ligase is able to bind an E2 ligase that transfers ubiquitin moieties to lysine residues on the POI surface, thereby labelling that protein for cellular waste disposal [11,12]. Consequently, lysine polyubiquitination is then recognized by the 26S proteasome, and the targeted POI is degraded, while the PROTAC itself is released and able to form another ternary complex (Figure 1) [13,14]. In this regard, PROTACs are able to go through multiple cycles of complexing with the POI and E3 ligase, facilitating the catalytic degradation of the POI in sub-stoichiometric amounts [15].

When designing and developing novel, effective PROTAC compounds, picking the right linker motif plays a major role (“linkerology”). Since both the POI- and E3 ligase-addressing ligands are mostly set in their physicochemical properties, variation in the used linker is often key to influencing PROTAC features, such as biological activity, physicochemical parameters coding for solubility, cellular permeability, and metabolic stability [16,17,18]. Of note, empirically finding the right linker length is often key, and the absence or presence of a single atom within the linker moiety can differentiate whether a PROTAC is bioactive or not. In line with this notion, linkers that are too short may not allow each ligand to interact with their respective protein due to steric hindrance, which makes it impossible to form the required ternary complexes. As a result, the respective compounds do not lead to polyubiquitination and subsequent degradation. If the linker is too long, ternary complexes may be formed, although POI degradation may still not occur due to the formation of “non-ideal” proximity between the POI and E3 ligase being insufficient to result in ubiquitination [14,19].

### CK1 Isoforms as Proteins of Interest for PROTAC Design

The highly conserved and ubiquitously expressed CK1 family of serine/threonine kinases consists of seven members (α, β, γ1, γ2, γ3, δ, and ε). CK1 isoforms and various splice variants in humans are known to be linked to several regulatory roles such as DNA processing and repair, proliferation, apoptosis, cell differentiation, and circadian rhythm [20,21,22,23,24]. One of the most researched and best-characterized CK1-regulated mechanisms to date is the canonical Wnt/β-catenin pathway, which mainly controls cell proliferation. If Wnt receptors are stimulated through the binding of respective Wnt ligands, β-catenin is translocated to the nucleus, which ultimately leads to the transcription and expression of Wnt-dependent target genes. However, if Wnt ligands are absent, a “destruction complex” consisting of adenomatous polyposis coli (APC), AXIN, CK1α, and glycogen synthase kinase 3 (GSK3) is formed in the cytoplasm to capture β-catenin and induce its degradation through the phosphorylation of CK1α and GSK3 [23,25,26]. Additionally, CK1δ/ε controls Wnt signalling via the phosphorylation of Dishevelled (DVL1/2/3) proteins [27]. The dysregulation of the CK1δ isoform is known to be linked to various forms of diseases, e.g., cancer (i.e., colon, pancreatic, breast, and ovarian cancer), as well as neurodegenerative and metabolic diseases, rendering this isoform a highly attractive target for medicinal chemistry-based approaches [28,29,30].

**Scheme 1:**
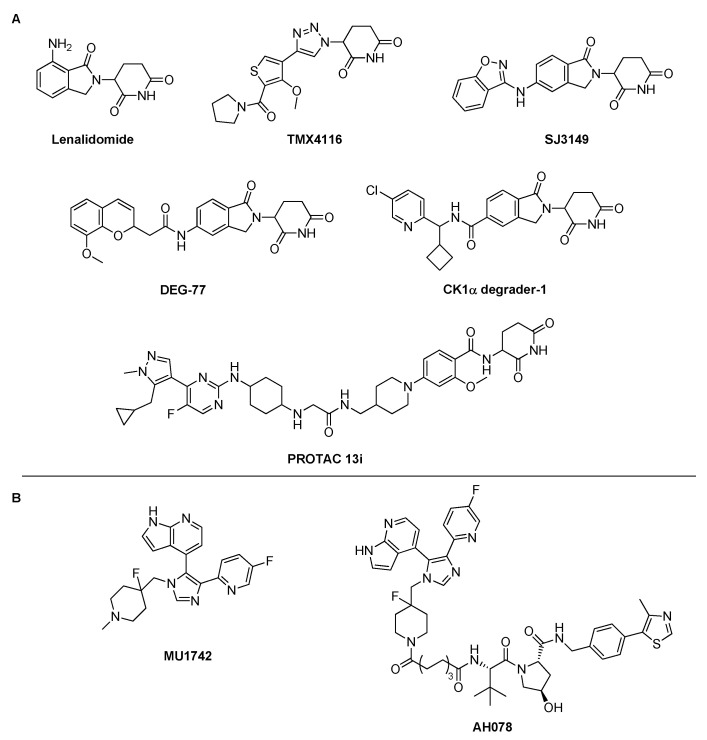
(**A**) Chemical structures of CK1α degrader probes, including both molecular glue (lenalidomide, TMX4116, SJ3149, DEG-77, CK1α degrader-1) and PROTAC (PROTAC 13i) compounds [31,32,33]. (**B**) Chemical structure of CK1 “tear drop” inhibitor (MU1742) utilized to synthesize degrader compound AH078 containing alkyl linker as well as VHL ligand [34].

When it comes to CK1 targeted protein degradation (TPD), so far, compounds targeting CK1α have been reported, including CK1 molecular glue degraders like lenalidomide, TMX4116, SJ7095/SJ3149, DEG-77 [31], CK1α degrader-1 [32], and PROTAC 13i [33] (Scheme 1). While our work was in progress, in December 2024, Haag et al. reported their degrader compounds targeting CK1δ and CK1ε. They demonstrated that although compound AH078 showed a reported DC_50_ value of 0.55 μM and D_max_ of 70% towards CK1δ, all compounds lacked isoform selectivity regarding CK1δ/ε, likely due to the high homology between their respective kinase ATP-binding domains [23,28,34].

**Scheme 2:**
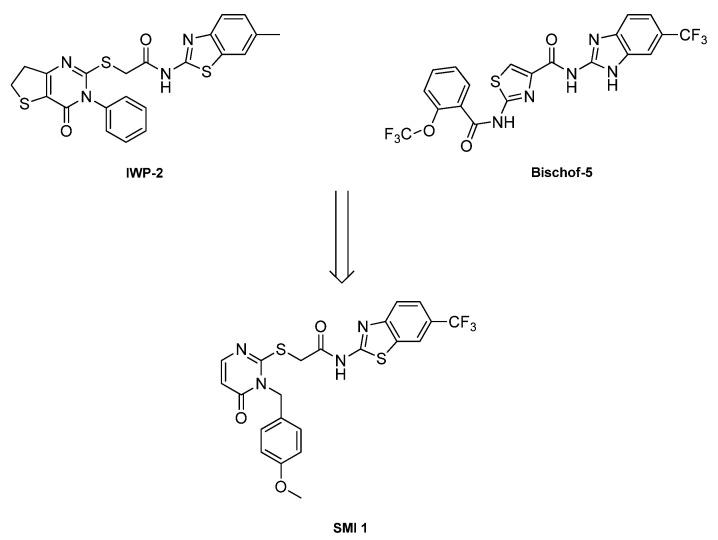
Combining relevant moieties from the chemical structures of IWP-2 [37,38], and Bischof-5 [39] led to the discovery of SMI 1, a highly CK1δ-specific and cellular effective inhibitor with a CK1δ IC_50_ value of 0.09 ± 0.01 μM [36].

Motivated especially by the lack of CK1δ-specific degraders at the inception of our project, we started a synthetic project working towards the development of a platform containing various types of linker motifs of different lengths and variable types of E3 ligase ligands. In contrast to the “tear drop” type of CK1δ inhibitor used by Haag et al. [34] in their recently reported CK1 PROTACs, we set out to employ a “linear” CK1δ inhibitor moiety in our project, since former efforts towards “linear” CK1δ/ε-specific inhibitors in our group led to the discovery of SMI 1 (Scheme 2) [35,36]. The CK1δ-specific inhibitor is based on the scaffold of established IWP compounds (i.e., IWP-2) [37,38] and known CK1-specific benzimidazole-based inhibitors such as Bischof-5 [39]. Such degrader probes would be highly useful to further study CK1 isoform-dependent processes, e.g., in cancer cells. Based on strong SAR data and having structural data of a ligand–protein complex for SMI 1 in hand, we first aimed to identify a suitable linker attachment position at the CK1δ-specific inhibitor scaffold.

## 2. Results and Discussion

### 2.1. CK1δ PROTAC Design Based on SMI 1

**Figure 2:**
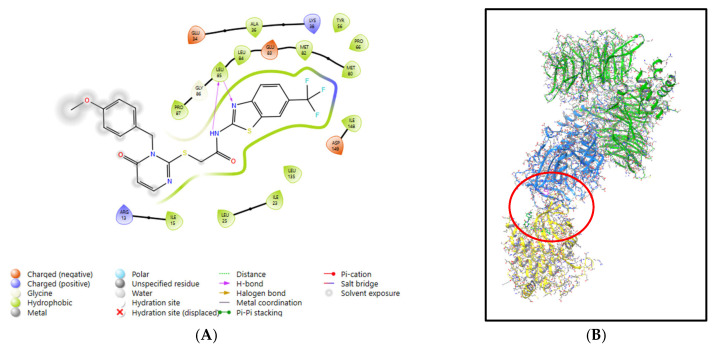

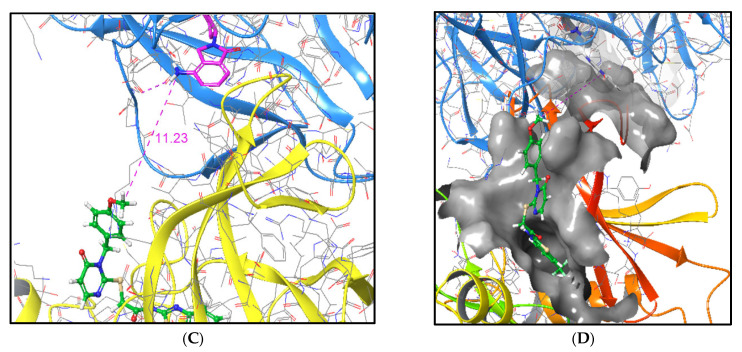
Molecular modelling to guide the design of candidate CK1δ-PROTAC compounds. (**A**) SMI 1 docked in the ATP-binding pocket of CK1δ (pdb 5OKT) [35] illustrated as a 2D ligand interaction diagram showing the key interactions of the binding pose of SMI 1 within the ATP pocket of CK1δ (bidental hinge-binding motif to Leu85 is highlighted). The benzyl moiety of SMI 1 with the p-MeO position is sitting in the solvent-open area (grey dots), indicating this position to be suitable for linker attachment. (**B**) The X-ray-defined apoCK1α/CRL4(CRBN)/lenalidomide complex (pdb 5FQD) [41] was used to herein replace the apoCK1α part via alignment with the CK1δ structure (pdb 5OKT) shown in yellow. The red labelled area shows the protein interaction site of the CK1δ-SMI 1/CRBN/lenalidomide model with close-up details in (**C**) CK1δ (yellow) with docked SMI 1 (green) in the ATP-binding pocket/Crl4(CRBN) (blue/green)/lenalidomide (magenta) complex. (**C**) The measured distance (dotted purple line) between the p-MeO function of SMI 1 (green) and the amino nitrogen atom of lenalidomide bound to CRL4(CRBN) (blue) revealed approximately *d* = 11 Å. (**D**) A close-up view of the highlighted area (side perspective) also showing the solvent-accessible protein surface.

Having established SMI 1 as a highly specific and potent inhibitor towards CK1δ, we set out to integrate this scaffold into the development of the desired CK1δ-targeting PROTACs. SAR data and initial molecular modelling using our ligand–CK1δ structure complex (pdb 5OKT) [35] suggested the p-MeO benzyl position of SMI 1 as a suitable position for linker attachment. This moiety occupies the solvent-open hydrophobic region II (HRII) of the CK1-ATP-binding pocket (Figure 2A), thus offering a strong rationale for effective CK1δ PROTAC design. Besides defining the *p*-benzyl moiety of the POI ligand as the desired function for linker attachment, we chose well-known thalidomide derivatives to address Cereblon (CRBN) as the employed E3 ligase for the first set of potential CK1δ-PROTACs. Having defined both the POI and E3 ligands, we next focused on the linker moiety itself. As previously described, the design of the linker is often crucial to the efficacy of PROTACs, due to the linker’s strong influence on both physicochemical properties and PROTAC functionality itself (i.e., forming of a stable ternary complex, etc.) [2,40]. In order to rationalize a suitable position for eligible linker moieties in SMI 1, we performed molecular modelling. As no experimentally resolved structure of the CK1δ–CRL4(CRBN) complex has been reported to date, the CK1α–CRL4(CRBN) [41] complex was used as a structural template, and CK1α was subsequently replaced by CK1δ to model the corresponding interaction, with SMI 1 docked into the ATP-binding pocket (Figure 2B–D). Herein, we determined an approximately 11 Å distance between the p-MeO-benzyl position of SMI 1 and the nitrogen atom of lenalidomide in its CRBN-binding pose. Noteworthy, our simple static modelling approach may only serve as a hint for PROTAC design and rationalizes a suitable attachment position of the linker moiety in SMI 1. Hence, being aware of lenalidomide’s role as a molecular glue in the original ligand–protein structure (pdb 5FQD) and taking conformational changes in an actual CK1δ-PROTAC-CRBN complex into account, we considered the modelled distance in our CK1δ-PROTAC approach only as a rough estimation. Therefore, we did not specifically focus on the modelled 11 Å distance regarding the linker length in the proposed PROTAC but instead designed a variable set of linker moieties to cover a broad range for the studied candidate compounds.

**Scheme 3:**
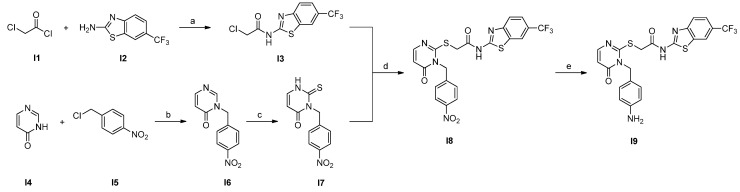
Synthesis of POI ligand and intermediate **I9**: (a) 1.1 equiv. TEA in CH_2_Cl_2_, rt, 24 h; (b) 1.5 equiv. KOH and nano iron (cat.) in abs. DMSO under inert atmosphere, 110 °C, 2 h; (c) (i) 2.5 equiv. phenyl chlorothionoformate and 1.1 equiv. sodium bicarbonate in ethyl acetate/water (1:1), rt, overnight; (ii) 6.0 equiv. TEA in methanol, 80 °C, 4 h; (d) 3.0 equiv. TEA in abs. DMF, 80 °C, 2 h; (e) H_2_ (3 bar) and 10% (m/m) Pd/C in methanol, rt, 24 h.

**Scheme 4:**
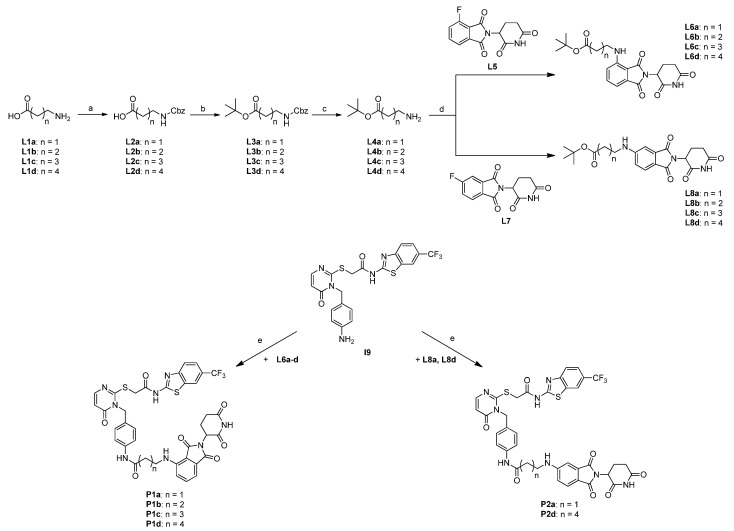
Synthesis of first set of PROTAC compounds **P1a–d**, as well as **P2a** and **P2d** containing alkyl linker motifs: (a) 1.1 equiv. benzyl chloroformate in 2 N sodium hydroxide solution, rt, overnight; (b) 1.1 equiv. phosphoryl chloride in ^t^BuOH/pyridine (3:2), rt, overnight; (c) H_2_ and 10% (m/m) Pd/C in methanol, rt, overnight; (d) 3.0 equiv. DIPEA in DMSO, 90 °C, 14 h; (e) (i) 1 mL TFA in CH_2_Cl_2_, rt, 1 h; (ii) 1.0 equiv. EDC × HCl, 0.1 equiv. HOBt, 1.0 equiv. DMAP and 5.0 equiv. DIPEA in MeCN, rt, 48 h.

Motivated by the modelling hypothesis based on the ligand–protein structure (pdb 5OKT), in our first attempts, we aimed at the designed CK1δ-PROTACs on the p-MeO benzyl moiety of SMI 1 to attach the linker via ether moieties as well. However, our various attempts to cleave the p-MeO position of SMI 1 did not result in sufficient yields of the corresponding phenol compound that would be needed. Therefore, we started implementing the respective aniline derivative **I9** to attach the linker as amides (see Scheme 3 and Scheme 4). Noteworthily, the SAR of the former project regarding SMI 1 demonstrated the variable substitution of the *p*-position of the benzyl moiety to impact CK1δ/ε-isoform selectivity. Accordingly, attaching different linker moieties to this position via an amide instead of ether is anticipated to potentially influence the isoform selectivity of the corresponding PROTACs and is considered in due course of this project.

### 2.2. Synthesis of Key Aniline CK1δ POI Ligand ***I9***

In order to generate the favoured aniline intermediate (**I9**), the synthesis of the corresponding p-nitro group compound (**I8**) was performed according to the general convergent method for synthesizing SMI 1. The subsequent reduction of the said p-nitro group delivered targeted **I9** (Scheme 3) [36,42,43,44]. Briefly, for the general synthesis strategy, 2-chloracetylchloride was reacted with the benzothiazole building block **I2** to yield amide **I3**. On the other hand, precursors **I4** and **I5** were implemented under basic conditions with nano iron in order to provide the selective NH substitution of tautomeric **I4** to generate intermediate **I6**. Next, the thioxo group was introduced using phenyl chlorothionoformate to afford intermediate **I7**. By coupling benzothiazole **I3** with the substituted 2-thiouracil derivative **I7**, we were able to generate intermediate **I8**. Finally, the nitro group in the p-position was catalytically reduced with hydrogen to the amine function in order to afford the desired key intermediate **I9**. It is to be noted that hydrogen pressure had to be increased to 3 bar to yield the intermediate **I9** in sufficient yields.

### 2.3. Synthesis of Candidate CK1δ PROTACs Containing Alkyl Linker Motifs

As previously described, most PROTACs published so far contain either PEG or alkyl linker motifs [17]. Due to their chemical accessibility and the extensive literature on attaching these linkers to thalidomide derivatives, we decided to use alkyl linker motifs in our first set of PROTAC compounds. The synthesis of the intermediate compounds **L2a–d** through **L4a–d** was performed based on a method reported by Schmuck et al. (Scheme 4) using the *N*-Cbz-protection of the corresponding amino acid under basic conditions using benzyl chloroformate in the first step and generating the respective *tert*-butyl ester compound afterward by using *tert*-butyl alcohol, pyridine, and phosphoryl chloride. In the final step of alkyl linker preparation, the Cbz protecting group was cleaved by stirring intermediates **L3a–d** and 10% (m/m) Pd/C under a hydrogen atmosphere to yield intermediate compounds **L4a–d** [45]. Initially, the syntheses resulted in unsatisfactory yields, which we were able to increase by extending the reaction time by letting the mixture after each step be stirred overnight. After successfully preparing the linker motifs, the compounds were coupled according to Kim et al. (Scheme 4) with the regioisomeric thalidomide derivatives 2-(2,6-dioxopiperidin-3-yl)-4-fluoroisoindoline-1,3-dione and 2-(2,6-dioxopiperidin-3-yl)-5-fluoroisoindoline-1,3-dione, respectively, under basic conditions to generate the desired intermediates **L6a–d** and **L8a–d** [46].

Having the variable linker–thalidomide compound series **L6a–d** and **L8a–d** in hand, we aimed to finally generate the first two sets of PROTAC compounds by coupling the respective intermediates **I9** and **L6a–d** or **L8a–d** to the inhibitor scaffold via an amide function. Therefore, the *tert*-butyl ester of the linker motifs needed to be cleaved first to afford the corresponding carboxylic acid compounds. Hence, in a one-pot approach, the intermediate compounds were dissolved in CH_2_Cl_2_, and TFA was added [46]. The solvent was evaporated, and the residue was dried under high vacuum to afford the carboxylic acid compounds in quantitative yields. The first attempts of amide coupling itself were performed according to Ghosh et al. (Scheme 4) using 1.5 equiv. HATU and 5.0 equiv. DIPEA but only with insufficient yields. We therefore decided to instead perform amide coupling by employing EDC hydrochloride, HOBt, DMAP, and DIPEA, which led to significantly higher, though still low, yields in the range of 6–24%. However, we considered these yields to be sufficient in the context of initial test compounds [47].

**Figure 3:**
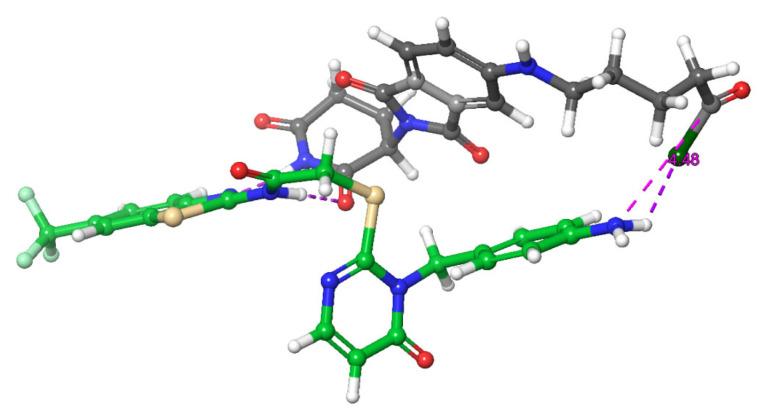
Exemplary molecular modelling suggesting complex-forming interactions between **I9** (green) and **L8c** (grey), thereby potentially preventing the amide coupling reaction.

Even though the employed amide coupling method using EDC, HOBT, and DMAP was successful for the synthesis of the initial degrader compounds **P1a–d** (linker lengths ranging from three to six carbon atoms), we were, however, unable to generate the PROTAC compounds **P2b–c** (four and five carbon atoms, respectively) using this method. To follow up on these molecules, we first verified the functionality of compounds **L8b–c** to actually undergo the desired amide coupling by utilizing p-toluidine as a dummy reactant for compound **I9**. Interestingly, both syntheses were performed successfully with quite good yields in the range of 85%. Next, various conditions were tested in order to yield compounds **P2b–c** (Table 1) but all with no success. Since the dummy reaction yields were excellent, we hypothesized that the actual inhibitor scaffold somehow prevents coupling, especially with these linker moieties (four and five carbon atoms), probably by forming complexes, thus hindering the actual amide coupling reaction. In line with this notion, molecular modelling suggested interactions between the hinge binder of **I9** and the imide structure of the employed thalidomide/pomalidomide (Figure 3), potentially complicating the formation of the desired amide bond.

Therefore, we performed coupling in various solvents and conditions and also added glutarimide in an attempt to saturate the hinge binder of **I9** so that the amide coupling moieties may be accessible to each other. However, in our hands, all these attempts did not lead to the successful formation of the desired degrader compounds. Hence, the exact molecular background of this method delivering various lengths of alkyl linker compounds, with the exception of compounds **P2b–c**, remains unclear. We next decided to focus on generating a set of CK1δ-PROTACs containing PEG linker motifs, due to their overall likely higher solubility and cell permeability. Furthermore, we favoured these especially regarding PROTAC compounds containing VHL ligands [40], which we intend to integrate into our synthetic platform in the future.

### 2.4. Synthesis of Candidate CK1δ PROTACs Containing PEG Linker Motifs

**Scheme 5:**
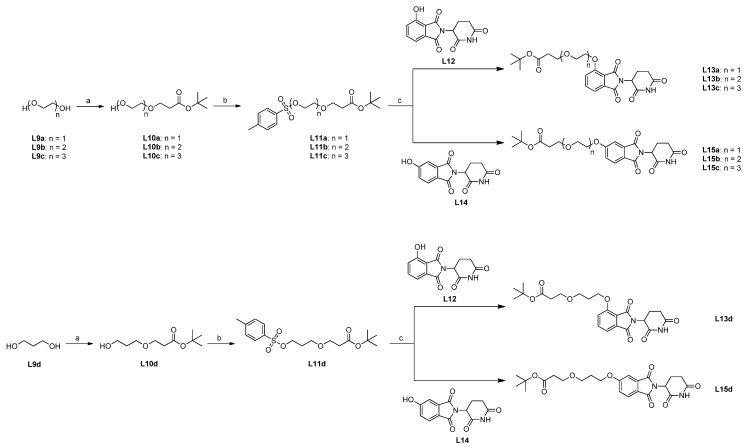
Synthesis for compounds **L13a–d** and **L15a–d** containing PEG linker motifs: (a) 1.0 equiv. *tert*-butyl acrylate and 0.04 equiv. Triton B solution (40% in water), rt, 120 h; (b) 1.5 equiv. tosyl chloride, 3.0 equiv. TEA and 0.1 equiv. DMAP in CH_2_Cl_2_, rt. 24 h; (c) 1.5 equiv. potassium bicarbonate and 0.1 equiv. sodium iodide in DMF, 80 °C, 16 h.

**Scheme 6:**
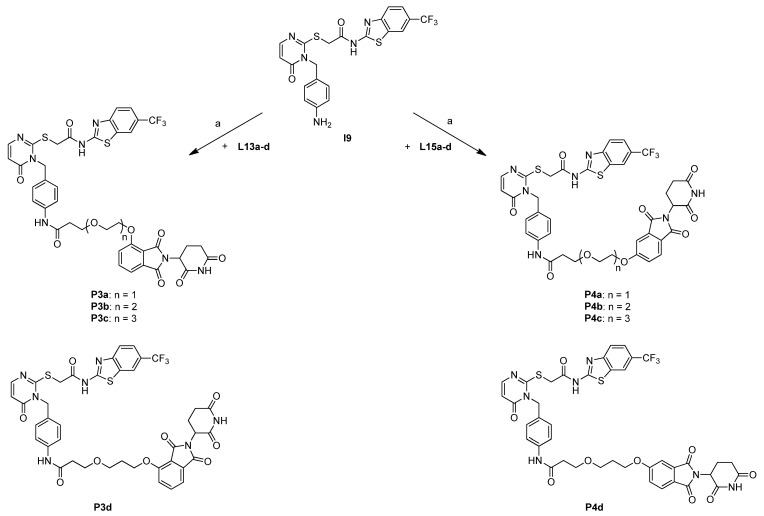
Synthesis for the second and third sets of PROTAC compounds **P3a–d** and **P4a–d** containing PEG linker motifs: (a) (i) 1 mL TFA in CH_2_Cl_2_, rt, 1 h; (ii) 1.0 equiv. EDC × HCl, 0.1 equiv. HOBt, 1.0 equiv. DMAP and 5.0 equiv. DIPEA in MeCN, rt, 48 h.

We sought to couple our POI ligand **I9** and the thalidomide derivatives via different types of PEG moieties. First, the corresponding diol precursors were coupled with *tert*-butyl acrylate according to the method by Cromm et al. (Scheme 5) in the sense of a Michael Addition under basic conditions [48]. Noteworthy, the diol precursors were used in a fivefold excess to prevent Michael Addition from occurring on both primary alcohol functions. According to the reported method, in the first attempts of attaching the PEG linker motifs to the thalidomide derivative, the remaining primary alcohol function was iodinated using iodine, triphenylphosphine, and imidazole [48] and then coupled with 2-(2,6-dioxopiperidin-3-yl)-4-hydroxyisoindoline-1,3-dione under basic S_N_ conditions. The latter step, however, resulted only in insufficient yields of approximately 2–10% for the targeted intermediates **L10a–d**. The main reason for this was the significant formation of a side product showing a dual linker substitution of **L12** at both the acidic phenol OH and the imide nitrogen NH of the thalidomide part (see Scheme S1, Supplementary Materials). Therefore, we opted to first protect the primary alcohol functions of intermediates **L10a–d** with a tosyl moiety as a leaving group in order to avoid substitution at the imide nitrogen based on the sterically demanding tosyl moiety (Scheme 5). This strategy proved to be successful, and by coupling these tosyl-protected intermediates with 2-(2,6-dioxopiperidin-3-yl)-4-hydroxyisoindoline-1,3-dione, we were able to increase the yield of the corresponding intermediates **L13a–d** to up to 80% while simultaneously keeping dual substitution to a minimum (Scheme 5). Finally, the syntheses of the newly designed CK1δ-PROTACs containing PEG linker motifs were performed according to the method used for producing the first two sets, **P1a–d**, **P2a**, and **P2d** (Scheme 6). As formerly observed in these final steps for the alkyl series, the coupling reactions for the PEG series yielding **P3a–d** and **P4a–d** also resulted in low but still sufficient yields for the biological testing of PROTACs.

### 2.5. Biological Results

#### 2.5.1. Differential Degradation of CK1δ/ε by Different PROTACs

**Figure 4:**
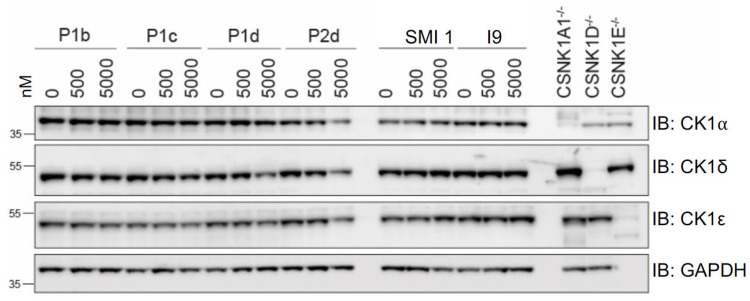
The dose response of PROTAC compounds **P1b**, **P1c**, **P1d**, and **P2d** at 0.5 and 5 μM. Wildtype (WT) U2OS cells were treated with DMSO, I9, SMI 1, or the indicated PROTACs at 0.5 and 5 μM for 24 h prior to lysis. Extracts (20 µg) were resolved by 4–20% Bis-Tris precast SDS-PAGE, transferred to nitrocellulose membrane, and immunoblots were performed with the indicated antibodies. GAPDH was employed as a loading control. Extracts from DLD-1 CSNK1A1^−/−^, CSNK1D^−/−^, and CSNK1E^−/−^ cells were included to validate CK1α, CK1δ, and CK1ε antibodies, respectively. All blots are representative of three independent biological repeats.

We tested the first series of PROTACs that we synthesized for their ability to degrade CK1α, CK1δ, and CK1ε in wildtype (WT) U2OS osteosarcoma cells at 500 and 5000 nM for 24 h prior to lysis. An equivalent volume of DMSO that was used to dilute each compound applied to cells was included as a negative control. Compared to DMSO-, I9-, and SMI 1-treated controls, **P1d** caused a reduction in the levels of both CK1δ and CK1ε at 5 µM but no change in the levels of CK1α (Figure 4), demonstrating isoform selectivity. **P2d** caused a reduction in the levels of CK1α, CK1δ, and CK1ε at 5 µM (Figure 4). In contrast, **P1b** and **P1c** did not cause any changes in the levels of CK1α, CK1δ, and CK1ε at any concentrations (Figure 4). The specificity of antibodies recognizing CK1α, CK1δ, and CK1ε was confirmed by immunoblotting extracts from the respective knockout DLD-1 cells (Figure 4). The levels of GAPDH did not change with any compound treatment (Figure 4).

**Figure 5:**
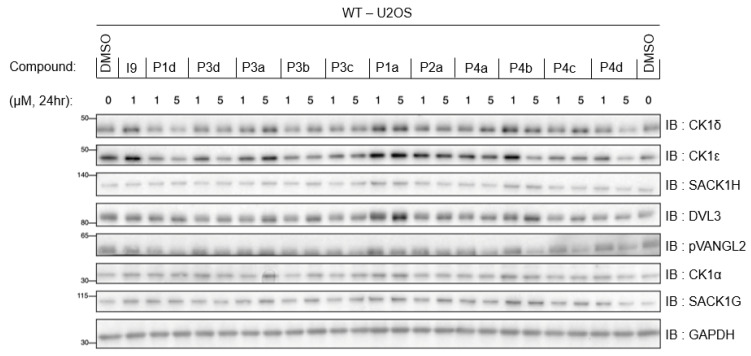
Dose response of all PROTAC compounds at 1 and 5 μM. Wildtype (WT) U2OS cells were treated with DMSO, reference inhibitor I9 at 1 μM, or indicated PROTACs (**P1d** to **P4d**) at 1 and 5 μM for 24 h prior to lysis. Extracts (20 µg) were resolved by 4–20% Bis-Tris precast SDS-PAGE, transferred to nitrocellulose membrane, and immunoblots were performed with indicated antibodies. GAPDH was employed as loading control. All blots are representative of three independent biological repeats.

Encouraged by **P1d** causing reduced levels of CK1δ and CK1ε, once we completed the synthesis of the remaining molecules, we sought to test the remaining series of PROTACs side by side along with **P1d** in WT U2OS cells at 1 µM and 5 µM concentrations for 24 h, along with the inhibitor warhead I9 as a control, for their ability to degrade CK1δ and CK1ε. The treatment of U2OS cells with 1 µM I9 slightly increased the levels of CK1δ but did not substantially alter the levels of CK1ε compared to DMSO treatment (Figure 5). **P1d**, **P3d**, and **P4d** led to a dose-dependent reduction in the levels of CK1δ and CK1ε at 1 µM and 5 µM compared to DMSO-treated controls (Figure 5). **P3c** and **P4b** both displayed a slight dose-dependent reduction in the levels of CK1δ/ε, while **P3a**, **P3b**, **P1a**, **P4a**, and **P4c** showed no robust dose-dependent decrease in the levels of CK1δ/ε (Figure 5).

DVL3 and Vangl2 are known CK1δ/ε substrates [27,49]. Therefore, the inhibition or degradation of CK1δ/ε would be expected to block the phosphorylation of these proteins. DVL3 is heavily phosphorylated by CK1δ/ε, causing an upward electrophoretic mobility shift in DVL3. No robust DVL3 mobility shift was observed with 1 µM I9 or any of the PROTACs, while a minor downward mobility shift was observed with most PROTACs at 5 µM (Figure 5), potentially suggesting the inhibition of CK1δ/ε at 5 µM. A lack of robust electrophoretic mobility shift in DVL3 evident here was possibly due to the use of 4–20% polyacrylamide Bis-Tris precast gels (Invitrogen), and we subsequently improved this for further experiments by using 8% polyacrylamide gels. Vangl2 is phosphorylated on Ser84 by CK1δ/ε [50]. The treatment of cells with 1 µM I9 caused a reduction in the pVangl2 signal compared to the DMSO-treated control (Figure 5), suggesting the successful inhibition of CK1δ/ε. **P1d**, **P3d**, and **P4d**, as well as all other PROTACs, especially at 5 µM, led to a reduction in the pVangl2 signal compared to the DMSO-treated control (Figure 5). This data suggests that most PROTACs lead to the inhibition of CK1δ/ε, even if not all of them cause the degradation of CK1δ/ε (Figure 5).

CK1δ/ε co-exist in cells with SACK1A, B, E, and H (formerly known as FAM83A, B, E, and H) [51]. The treatment of U2OS cells with PROTACs did not yield the co-depletion of SACK1H, even under conditions where certain PROTACs led to the depletion of CK1δ/ε (Figure 5). In similar assays, CK1α degraders DEG-77 and SJ3149 have been shown to co-degrade CK1α-interactors SACK1B, SACK1D, SACK1F, and SACK1G [52]. The levels of CK1α or SACK1G, which only interacts with CK1α but not CK1δ/ε, were also not substantially affected by any of the PROTACs (Figure 5). The levels of GAPDH, employed as a loading control, were unaltered by any of the treatment conditions (Figure 5).

#### 2.5.2. PROTACs as Inhibitors of CK1δ/ε

**Figure 6:**
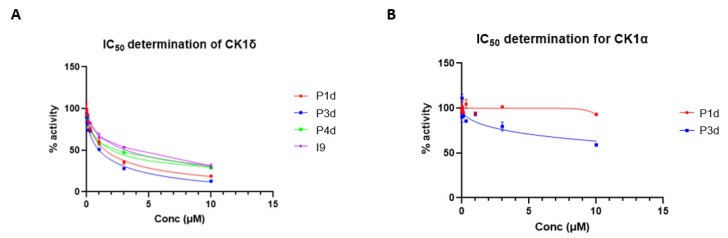
In vitro IC50 determination for the indicated compounds in vitro against CK1δ (**A**) and CK1α (**B**) kinase activity using a peptide substrate. All assays were performed by the MRC PPU International Centre for Kinase Profiling.

Despite some PROTACs not causing the degradation of CK1δ/ε, all PROTACs at 5 µM caused a robust reduction in the levels of pVangl2 and a noticeable downward mobility shift in DVL3 (Figure 6A), suggesting that the PROTACs inhibited CK1δ/ε. We evaluated the potency of CK1δ inhibition in vitro by the three best degraders, **P1d**, **P3d**, and **P4d**, relative to warhead inhibitor compound **I9**. An in vitro kinase assay for CK1δ using a peptide substrate was conducted by the MRC PPU International Centre for Kinase Profiling (https://www.kinase-screen.mrc.ac.uk/, accessed on 1 August 2025). The IC_50_ for CK1δ inhibition in vitro for P3d was 1.96 µM, while it was ~2.2 µM for **P1d** and **P4d** and 3.9 µM for CK1δ inhibitor I9 (Figure 6A), suggesting that the PROTACs were more potent inhibitors of CK1δ in vitro than the inhibitor warhead molecule. Without further controls, these findings make it hard to interpret whether the cellular effects of PROTACs are due to the chemical inhibition of CK1δ/ε or the modest degradation of CK1δ/ε or the combined effects of both. In similar assays in vitro, we showed that **P1d** and **P4d** did not inhibit CK1α kinase activity substantially (Figure 6B), suggesting that the compounds were selective against CK1δ/ε.

#### 2.5.3. Extended Dose–Response Analysis of CK1δ/ε Degradation by Best Degrader **P1d** Across Multiple Cells

**Figure 7:**
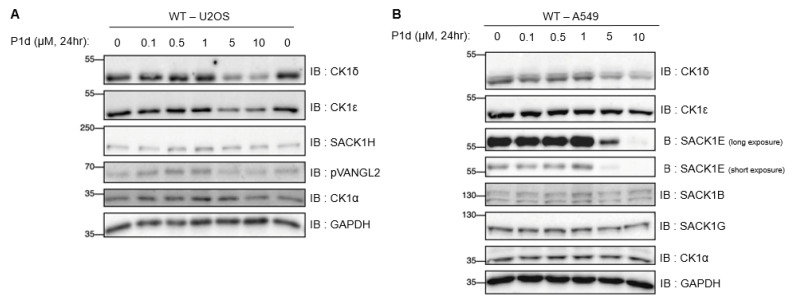
Dose response of compound **P1d** at 0–10 μM in various cell lines. (**A**) Wildtype (WT) U2OS and (**B**) A549 adenocarcinoma cells were treated with DMSO or **P1d** (from 0.1 to 10 µM) for 24 h prior to lysis. Extracts (20 µg) were resolved by SDS-PAGE, transferred to nitrocellulose membrane, and immunoblots were performed with indicated antibodies. All blots are representative of three independent biological repeats.

The initial tests of PROTACs in cells revealed that **P1d** showed the most potent *CK1δ/ε* degradation. Therefore, a comprehensive dose–response analysis of **P1d** was performed in multiple cell lines to assess *CK1δ/ε* degradation and downstream consequences. In U2OS cells, a robust degradation of CK1δ and CK1ε was observed at 5 and 10 µM when treated for 24 h (Figure 7A). Concomitantly, at the given concentrations, there was a clear reduction in the levels of pVANGL2 (Figure 7A), suggesting the inhibition of VANGL2 phosphorylation as CK1δ/ε was degraded. No substantial changes in CK1α, SACK1H, or GAPDH were observed at any concentrations of **P1d** (Figure 7A), suggesting the selective degradation of CK1δ/ε and no co-degradation of SACK1H. In A549 adenocarcinoma cells, **P1d** also caused a robust degradation of CK1δ and CK1ε at 5 and 10 µM (Figure 7A). Unlike U2OS cells, A549 cells express detectable levels of CK1δ/ε-binders SACK1B and SACK1E. No co-depletion of SACK1B was observed, but a robust co-depletion of SACK1E, which exists in complex with CK1δ/ε, at 5 and 10 µM was observed (Figure 7A), potentially suggesting the co-degradation of the CK1δ/ε-SACK1E complex. No substantial changes in the levels of CK1α and its interactor SACK1G or GAPDH were observed at any concentrations of **P1d** (Figure 7B).

#### 2.5.4. Kinetic Analysis of CK1δ/ε Degradation by **P1d**

**Figure 8:**
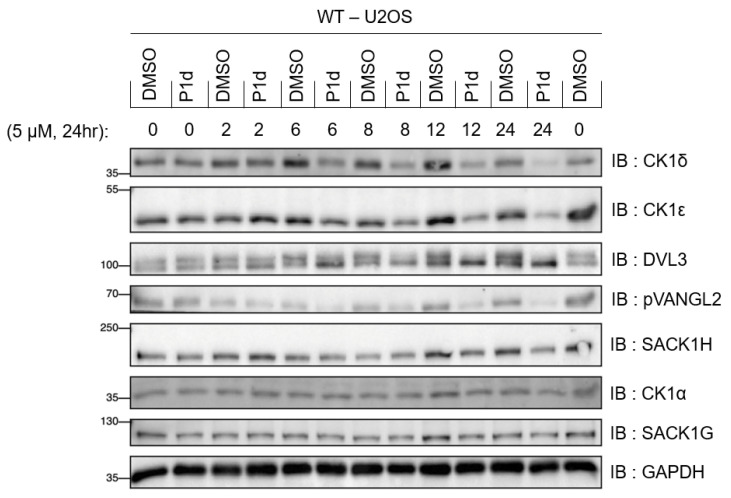
Kinetic analysis of *CK1δ/ε* degradation by compound **P1d** at 5 μM. Wildtype (WT) U2OS cells were treated with DMSO or 5 μM **P1d** for indicated times prior to lysis. Extracts (20 µg) were resolved by SDS-PAGE, transferred to nitrocellulose membrane, and immunoblots were performed with indicated antibodies. All blots are representative of three independent biological repeats.

Given the robust degradation of CK1δ/ε by **P1d** at 5 µM for 24 h, specific time points across 0–24 h were chosen to assess the kinetics of degradation. Compared to the DMSO control, **P1d** showed a time-dependent degradation of CK1δ/ε, where degradation started at 6 h and reached the maximal levels at 24 h following **P1d** treatment (Figure 8). In line with the degradation kinetics of CK1δ/ε, a downward mobility shift in DVL3 was observed, indicating the inhibition of DVL3 phosphorylation (Figure 8). Similarly, a reduction in the levels of pVANGL2 was observed at 2 h following **P1d** treatment, and it showed almost complete reduction after 6 h (Figure 8). Throughout the full 24 h with **P1d** treatment, there was no change in the levels of CK1α, its interactor SACK1G, or GAPDH (Figure 8).

#### 2.5.5. Establishing Mode of Action for **P1d**

**Figure 9:**
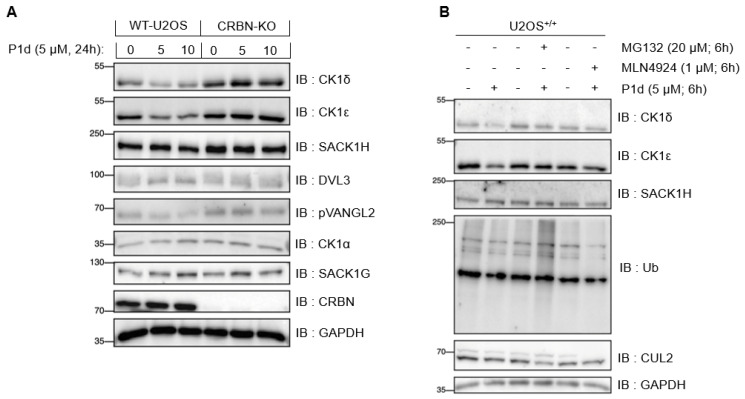
Mode of E3 ligase action compound **P1d** at 5 and 10 μM. (**A**) Wildtype (WT) or CRBN-knockout (CRBN-KO) U2OS cells were treated with DMSO or **P1d** at 5 and 10 µM for 24 h prior to lysis. (**B**) WT U2OS cells were treated with DMSO or **P1d** at 5 µM without or together with 1 µM MLN4924 (NAE1 inhibitor) or 20 µM of MG132 (proteasome inhibitor). For (**A**,**B**), extracts (20 µg) were resolved by SDS-PAGE, transferred to nitrocellulose membrane, and immunoblots were performed with indicated antibodies. All blots are representative of three independent biological repeats.

PROTACs recruit a native E3 ligase to ubiquitylate the POI, which triggers the UPS to successfully degrade the POI. We sought to explore the mode of action through which **P1d** led to the reduction in the levels of CK1δ/ε. As **P1d** uses a thalidomide warhead that binds to and recruits the CUL4^CRBN^ E3 ligase, we sought to investigate the requirement of CRBN by using CRBN-knockout (KO) U2OS cells. As shown earlier, the treatment of WT U2OS cells with 5 μM and 10 μM **P1d** led to a robust degradation of CK1δ/ε compared to the DMSO control (Figure 9A). In contrast, in CRBN-KO cells, generated through CRISPR/Cas9 genome editing [52] and validated by the absence of signals with anti-CRBN immunoblot, no CK1δ/ε degradation was observed upon the treatment of cells with 5 μM and 10 μM **P1d** (Figure 4, Figure 5, Figure 6, Figure 7 and Figure 8), confirming the requirement of CRBN for CK1δ/ε degradation. More importantly, WT and CRBN-KO U2OS cells also allowed us to determine the relative contribution of CK1δ/ε degradation vs. inhibition by 5 μM and 10 μM **P1d** on downstream signalling, as only inhibition would be possible in CRBN-KO cells. Excitingly, the extent of the downward mobility shift in DVL3 and reduction in VANGL2 phosphorylation observed in WT U2OS cells caused by **P1d** at both 5 μM and 10 μM was much lower in CRBN-KO cells (Figure 9A), suggesting that the degradation of CK1δ/ε in WT cells caused a better inhibition of downstream signalling than just the inhibition of CK1δ/ε in CRBN-KO U2OS cells caused by **P1d**. No reduction in the levels of SACK1H, CK1α, SACK1G, or GAPDH was observed by **P1d** in WT or CRBN-KO U2OS cells (Figure 9A). CUL4A^CRBN^ is activated by NEDDylation, which can be inhibited by the NAE1 inhibitor MLN4924. The treatment of U2OS cells with 1 μM MLN4924 blocked the degradation of CK1δ/ε caused by 5 μM **P1d** (Figure 9B). Similarly, the proteasome inhibitor MG132 at 20 μM also blocked the degradation of CK1δ/ε caused by 5 μM **P1d** (Figure 9B), suggesting the requirement of the proteasome for degradation. Used as positive controls, MLN4924 blocked the NEDDylation of CUL2, causing the disappearance of the higher-mobility species, and MG132 led to the accumulation of polyubiquitylated chains compared to DMSO controls (Figure 9B). Collectively, these data suggest that **P1d** causes CK1δ/ε degradation through CUL4^CRBN^ and the proteasome, and the degradation at the same PROTAC concentration appears to impact downstream signalling much more than inhibition.

#### 2.5.6. Correlation Between Computed and Experimental Linker Dimensions

To better understand the relationship between linker length and the observed efficacy in the degradation of our compounds, we compared the computationally estimated linker distance with experimentally realized structures. As previously mentioned, the calculated optimal linker length was determined to be approximately 11 Å (Figure 2C). Interestingly, our best-performing compounds (**P1d** and **P4d**, Figure 5) contain shorter linkers, measuring 7.413 Å and 8.492 Å, respectively (Table 2). Compounds **P3b** and **P4b**, whose linker lengths are the closest to the calculated value, exhibit minimal or no degradation under the tested conditions. Although the current modelling approach does not yet allow for a precise prediction of optimal linker dimensions, it provided a valuable guideline that helped to orient our experimental design and interpret the observed structure–activity trends. Since the current synthesis route only allowed for the successful generation of compounds containing shorter alkyl linkers, the exact influence of longer alkyl chains remains to be clarified. In future studies, we intend to synthesize and evaluate compounds with extended alkyl linkers to systematically investigate how increasing linker length affects both molecular stability and functional performance.

## 3. Materials and Methods

### 3.1. Molecular Modelling

**Figure 10:**
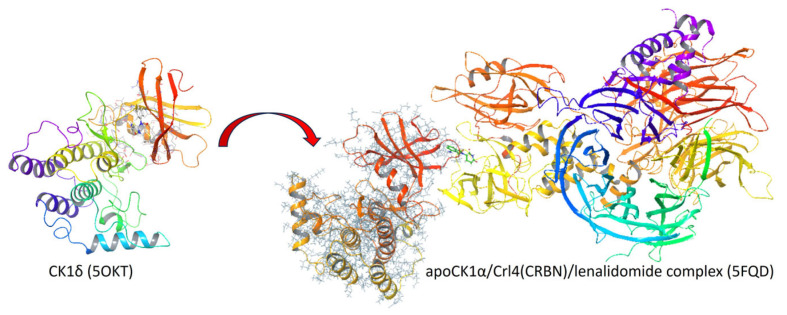
The structural replacement of CK1α in the CK1α–CRL4(CRBN) complex (pdb 5FQD) with CK1δ (pdb 5OKT) to model the corresponding CK1δ–CRL4(CRBN) interaction and to estimate the linker attachment site and required linker length.

Molecular modelling was performed using Schrödinger Maestro version 13.8.135, MMshare Version 6.4.135, Release 2023-4, Platform Windows-x64. The apoCK1α/Crl4(CRBN)/lenalidomide complex (pdb 5FQD) and CK1δ/ligand structure (pdb 5OKT) were imported and processed using the standard settings within the Protein Preparation Wizard [53]. The CK1α part of 5FQD was used to align CK1δ (pdb 5OKT) using the Protein Structure Alignment tool integrated in the Schrödinger suite, followed by the replacement of the CK1α part (Figure 10).

The molecular structure of ligand SMI 1 was prepared by minimization [54], followed by the use of the LigPrep tool (Schrödinger, LLC., New York, NY, USA, 2025). Docking with Glide (SP mode) [55] for SMI 1 into the receptor grid of the ATP-binding pocket of CK1δ (pdb 5OKT) revealed the binding pose. A 2D ligand–protein interaction diagram was used to illustrate the binding pose. In the 3D structure, the molecular measurement between the carbon atom of the p-MeO of SMI 1 and the amino nitrogen atom of lenalidomide bound to the Crl4(Crbn)/Ck1δ engagement site revealed a distance of approximately *d* = 11 Å, suggesting a putative linker position in SMI 1. The solvent-accessible protein surfaces of the structures were generated using the standard settings of the “surface binding site” tool.

### 3.2. Chemistry

#### 3.2.1. General Information

Reagents and solvents were purchased from abcr (Karlsruhe, Germany), Deutero (Kastellaun, Germany), Merck (Darmstadt, Germany), Roth (Karlsruhe, Germany), or TCI (Zwijndrecht, Belgium) with a purity of ≥95% and used as received.

Analytical RP-HPLC experiments for all intermediate compounds were performed using an Agilent 1290 (Agilent Technologies, Heilbronn, Germany) system (column: Agilent Poroshell, EC-C18, 2.7 μm, 50 × 3 mm) with 0.1% acetic acid in water (Solvent A) and acetonitrile (Solvent B) as eluents. All experiments were performed using a gradient with an increase of 5.88% Solvent B/min at a flow rate of 0.8 mL/min with a total run time of 16 min, injection volume of 1 μL, and detection between 210 and 400 nm.

Analytical RP-HPLC experiments for the PROTAC compounds were performed using an Agilent 1100 system (column: STAGROMA^®^C_18_ 3 μm, 125 × 4 mm; Stagroma AG, Reinach, Switzerland) with 0.01 M potassium dihydrogen phosphate buffer pH 2.3 (Solvent A) and acetonitrile (Solvent B) as eluents. All experiments were performed using a linear gradient with an increase of 3.33% Solvent B/min at a flow rate of 1.5 mL/min with a total run time of 30 min, injection volume of 10 μL, and detection at 254 nm.

The MS spectra of all intermediate compounds were measured using an AmaZon SL (Bruker, Billerica, MA, USA) system. The HR-MS characterizations of the PROTAC compounds were performed using a Q Exactive Plus Quadrupole-Orbitrap (Thermo Scientific, Darmstadt, Germany) system. The NMR spectra of all compounds were generated using a Bruker Avance III 400 system with the corresponding deuterated solvent as an internal standard.

#### 3.2.2. Synthesis of Compound **I3**

2-Amino-6-(trifluoromethyl) benzothiazole (**I2**, 1.00 equiv) and TEA (1.10 equiv) are dissolved in 5 mL dichloromethane. Chloroacetyl chloride (**I1**, 1.10 equiv) is dissolved in 5 mL dichloromethane and added to the reaction mixture dropwise over an hour. The reaction mixture is left to stir at RT overnight. The solvent is evaporated to afford the product, which is dissolved in ethyl acetate, washed with water, and concentrated in vacuo again. The product is recrystallized (ethanol/water 1:1) to afford compound **I3**.

#### 3.2.3. Synthesis of Compound **I6**

KOH (1.5 equiv) is ground, weighed, and left under vacuum for 45 min at RT. The system is flooded with argon afterwards. KOH is dissolved in 9.5 mL DMSO while stirring at 110 °C. 4(3H)-Pyrimidinone (**I4**, 1.00 equiv) and 4-Nitrobenzyl chloride (**I5**, 1.10 equiv) are dissolved in 10 mL DMSO and added to the reaction mixture via injection. Iron (nano) (0.10 equiv) is suspended in 0.5 mL DMSO and added via injection. Afterwards, the reaction mixture is left to stir at 110 °C for 2 h. After cooling down, the reaction mixture is decanted on ice and, if needed, neutralized with a few drops of 1M HCl. The aqueous layer is extracted with CH_2_ Cl_2_ [3 × 30 mL]. The organic layers are combined, washed with water [3 × 30 mL], and dried over sodium sulfate. The solvent is evaporated, and the crude product is purified via flash chromatography (silica gel, petroleum ether/ethyl acetate 80/20–0/100) to afford compound **I6**.

#### 3.2.4. Synthesis of Compound **I7**

Intermediate **I6** (1.00 equiv) and sodium bicarbonate (1.10 equiv) are dissolved in 30 mL EA/H2O 1:1. O-Phenyl chlorothionoformate (2.50 equiv) is added dropwise, and the reaction mixture is left to stir overnight afterwards. The aqueous layer is extracted with EA (3 × 15 mL). The organic layers are combined, washed with brine (3 × 15 mL), and dried over sodium sulfate. The solvent is evaporated, and the isolated intermediate stage is dissolved in 15 mL MeOH. TEA (6.00 equiv) is added, and the reaction mixture is left to stir at 80 °C for 4h. The solvent is evaporated, and the crude product is first purified via flash chromatography (dichloromethane/methyl alcohol). The crude is then suspended in ethyl acetate and filtered. The residue is dissolved in dichloromethane, and the solvent is evaporated to afford compound **I7**.

#### 3.2.5. Synthesis of Compound **I8**

Intermediate **I7** (1.00 equiv) and intermediate **I8** (1.00 equiv) are dissolved in 10 mL DMF. TEA (3.00 equiv) is added via injection, and the reaction mixture is left to stir at 80 °C for 2h. After cooling down, the reaction is quenched with water, and the mixture is extracted with EA (3 × 15 mL). The organic layers are combined, washed with brine (2 × 15 mL) and water (1 × 15 mL), and dried with sodium sulfate. The solvent is evaporated and the crude product purified via flash chromatography (silica gel, petroleum ether/ethyl acetate 80/20–0/100) to afford compound **I8**.

#### 3.2.6. Synthesis of Compound **I9**

Intermediate **I8** is dissolved in 20 mL of methyl alcohol. Palladium on activated carbon (10%, 0.1 equiv) is added to the reaction mixture. The atmosphere is exchanged for hydrogen (3 bar). The reaction is stirred at room temperature for 48 h. Afterwards, the catalyst is filtered off through a Celite pad, which is washed with methyl alcohol (3 × 20 mL). The filtrate and washings are combined, the solvent is evaporated, and the crude product is purified via flash chromatography (silica gel, dichloromethane/methyl alcohol 99/1–90/10) to afford compound **I9**.

#### 3.2.7. The General Procedure for the Synthesis of Linker Compounds **L2a–d**

The respective ϖ-Aminocarboxylic acid is dissolved in 2N sodium hydroxide solution (17 mL) and cooled in an ice bath to 0 °C. Once 0 °C is reached, benzyl chloroformate (1.10 equiv) and 2N sodium hydroxide solution (19 mL) are added within 2 min. The reaction mixture is left to stir at room temperature overnight and extracted with diethyl ether (4 × 40 mL) afterwards. The aqueous layer is separated, and the pH is adjusted to 2 with 2N hydrochloric acid. The resulting emulsion is extracted with ethyl acetate (3 × 30 mL). The organic phases are combined, washed with brine (3 × 20 mL), and dried over sodium sulfate. The solvent is evaporated to afford compounds **L2a–d**.

#### 3.2.8. The General Procedure for the Synthesis of Linker Compounds **L3a–d**

The respective compound **L2a–d** is dissolved in *tert*-butyl alcohol (45 mL). Pyridine (27 mL) is added, and the reaction mixture is chilled to −10 °C. Once −10 °C is reached, phosphoryl chloride (1.10 equiv) is added dropwise under vigorous stirring. The reaction mixture is stirred at −10 °C for 30 min and then left to stir at room temperature overnight. The reaction mixture is concentrated in vacuo and diluted with water (20 mL). The reaction mixture is extracted with ethyl acetate (3 × 50 mL) afterwards. The organic layers are combined; washed with water (3 × 55 mL), saturated sodium sulfate solution (3 × 55 mL), water again (3 × 55 mL), 2N potassium bisulfate solution (3 × 55 mL), and brine (2 × 30 mL); and dried over sodium sulfate. The solvent is evaporated to afford the corresponding compounds **L3a–d**.

#### 3.2.9. The General Procedure for the Synthesis of Linker Compounds **L4a–d**

The respective compound **L3a–d** is dissolved in methyl alcohol (30 mL). Palladium on activated carbon (10%, 0.10 equiv) is added, and the atmosphere is first exchanged for nitrogen and then exchanged for hydrogen. The reaction mixture is left to stir at room temperature overnight. The catalyst is filtered off through a Celite pad, which is washed with methyl alcohol (3 × 20 mL). The filtrate and washings are combined, and the solvent is evaporated to afford the corresponding compounds **L4a–d**.

#### 3.2.10. The General Procedure for the Synthesis of Compounds **L6a–d**

The respective compound **L4a–d** and 2-(2,6-dioxopiperidin-3-yl)-4-fluoroisoindoline-1,3-dione (1.00 equiv) are dissolved in DMSO (10 mL). N, N-Diisopropylethylamine (DIPEA, 3.0 equiv) is added, and the reaction mixture is stirred at 90 °C for 12 h. After cooling down, the reaction mixture is diluted with water (30 mL) and extracted with ethyl acetate (3 × 30 mL). The organic layers are combined, washed with brine (3 × 30 mL), and dried over sodium sulfate. The solvent is evaporated, and the corresponding crude product is purified by flash column chromatography (C18 reversed-phase silica gel, water/acetonitrile = 95/5–0/100). The solvent is evaporated to afford the corresponding compounds **L6a–d**.

#### 3.2.11. The General Procedure for the Synthesis of Compounds **L8a–d**

The respective compound **L4a–d** and 2-(2,6-dioxopiperidin-3-yl)-5-fluoroisoindoline-1,3-dione (1.00 equiv) are dissolved in DMSO (10 mL). N, N-Diisopropylethylamine (DIPEA, 3.0 equiv) is added, and the reaction mixture is stirred at 90 °C for 12 h. After cooling down, the reaction mixture is diluted with water (30 mL) and extracted with ethyl acetate (3 × 30 mL). The organic layers are combined, washed with brine (3 × 30 mL), and dried over sodium sulfate. The reaction mixture is concentrated in vacuo, and the corresponding crude product is purified by flash column chromatography (C18 reversed-phase silica gel, water/acetonitrile = 95/5–0/100). The solvent is evaporated to afford the corresponding compounds **L8a–d**.

#### 3.2.12. The General Procedure for the Synthesis of Linker Compounds **L10a–d**

The respective glycol and *tert*-butyl acrylate (1.00 equiv) are dissolved in acetonitrile (20 mL). Benzyltrimethylammonium hydroxide (Triton B) solution (40% in water, 0.04 equiv.) is added, and the reaction mixture is stirred at room temperature for 120 h. The solvent is evaporated, and the corresponding crude product is purified by flash column chromatography (silica gel, petroleum ether/ethyl acetate = 100/0–0/100). The solvent is evaporated to afford the corresponding compounds **L10a–d**.

#### 3.2.13. The General Procedure for the Synthesis of Linker Compounds **L11a–d**

The respective compound **L10a–d** and tosyl chloride (1.50 equiv) are dissolved in dichloromethane (5 mL). Triethylamine (3.00 equiv) and 4-dimethylaminopyridine (DMAP, 0.10 equiv) are added, and the reaction mixture is stirred at room temperature overnight. The solvent is evaporated, and the corresponding crude product is purified by flash column chromatography (silica gel, petroleum ether/ethyl acetate = 80/20–50/50). The solvent is evaporated to afford the corresponding compounds **L11a–d**.

#### 3.2.14. The General Procedure for the Synthesis of Compounds **L13a–d**

The respective compound **L11a–d** and 2-(2,6-dioxopiperidin-3-yl)-4-hydroxyisoindoline-1,3-dione (1.00 equiv) are dissolved in DMF (5 mL). Potassium bicarbonate (1.50 equiv) and sodium iodide (0.10 equiv) are added, and the reaction mixture is stirred at 80 °C for 12 h. After cooling down, the reaction mixture is quenched with water (10 mL) and extracted with ethyl acetate (3 × 20 mL). The organic layers are combined and dried over sodium sulfate. The solvent is evaporated, and the corresponding crude product is purified by flash column chromatography (C18 reversed-phase silica gel, water/acetonitrile = 95/5–0/100). The solvent is evaporated to afford the corresponding compounds **L13a–d**.

#### 3.2.15. The General Procedure for the Synthesis of Compounds **L15a–d**

The respective compound **L11a–d** and 2-(2,6-dioxopiperidin-3-yl)-5-hydroxyisoindoline-1,3-dione (1.00 equiv) are dissolved in DMF (5 mL). Potassium bicarbonate (1.50 equiv) and sodium iodide (0.10 equiv) are added, and the reaction mixture is stirred at 80 °C for 12 h. After cooling down, the reaction mixture is quenched with water (10 mL) and extracted with ethyl acetate (3 × 20 mL). The organic layers are combined and dried over sodium sulfate. The solvent is evaporated, and the corresponding crude product is purified by flash column chromatography (C18 reversed-phase silica gel, water/acetonitrile = 95/5–0/100). The solvent is evaporated to afford the corresponding compounds **L15a–d**.

#### 3.2.16. The General Procedure for the Synthesis of PROTAC Compounds **P1a–d**, **P2a**, **P2d**, **P3a–d**, and **P4a–d**

First, the respective compounds **L6a–d**, **L8a**, **L8d**, **L13a–d**, and **L15a–d** are dissolved in dichloromethane (1 mL). A total of 1 mL of trifluoroacetic acid is added, and the reaction mixture is stirred at room temperature for 1 h. The solvent is evaporated, and the product is dried under high vacuum to afford the corresponding carboxylic acid in a quantitative yield. The deprotection of the *tert*-butyl ester is verified via LC-MS, and the intermediate product is used without further purification.

The intermediate is dissolved in dichloromethane (10 mL), and intermediate **I9** (1.00 equiv) is added. 1-(3-dimethylaminopropyl)-3-ethylcarbodiimide hydrochloride (EDC, 1.00 equiv), Hydroxybenzotriazole (HOBt, 0.10 equiv), DMAP (1.00 equiv), and DIPEA (5.00 equiv) are added, and the reaction mixture is stirred at room temperature for 48 h. The solvent is removed, and the residue is dissolved in ethyl acetate (10 mL) and quenched with saturated sodium bicarbonate solution (30 mL). The aqueous layer is extracted with ethyl acetate (3 × 30 mL). The organic layers are combined and dried over sodium sulfate, and the solvent is evaporated. The crude product is purified by flash column chromatography twice (1: silica gel, petroleum ether/ethyl acetate 80/20–0/100, elution with dichloromethane/methyl alcohol 90/10; 2: c18 reversed-phase silica gel, water/acetonitrile 5/95–0/100) to yield the corresponding PROTAC compounds **P1a–d**, **P2a**, **P2d**, **P3a–d**, and **P4a–d**.

### 3.3. Biology

#### 3.3.1. Cell Culture, Drug Treatment, and Lysis

U2OS osteosarcoma, A549 adenocarcinoma, and DLD1 colorectal cancer cells were cultured in Dulbecco’s modified Eagle Medium (DMEM) supplemented with 10% (*v*/*v*) fetal bovine serum (FBS), 2 mM L-glutamine (Lonza, Basel, Switzerland), 100 U/mL penicillin (Lonza), and 0.1 mg/mL streptomycin (Lonza). Cells were incubated in a humidified incubator at 37 °C with 5% CO_2_ and handled under aseptic conditions within a laminar flow hood. For passaging, cells were washed in PBS and incubated with trypsin/EDTA at 37 °C until detached and transferred to fresh plates. CRBN^−/−^ U2OS cells were generated using a CRISPR/Cas9 genome editing approach as described previously [56]. CSNK1A1^−/−^ [52], CSNK1D^−/−^, and CSNKE^−/−^ DLD-1 cells were generated by K. Dunbar (Sapkota lab). All knockout cells were validated by immunoblotting (Figure 4) and DNA sequencing. All PROTAC compounds and small molecule inhibitors were dissolved in DMSO and were added directly to culture medium at the indicated concentrations and incubated with cells for the specified times prior to cell lysis. An equivalent volume of DMSO was added to cells as a treatment control. MG132 (Abcam; Ab141003, Cambridge, UK) and MLN4294 (Sigma-Aldrich, Dorset, UK; 5054770001) were also diluted in DMSO and applied to cells as above. Cells were lysed by removing culture media, washing twice with ice-cold PBS, and then scraping them directly into ice-cold NP-40 cell lysis buffer (50 mM Tris-HCl pH 7.5, 0.27 M sucrose, 150 mM NaCl, 1 mM EGTA, 1 mM EDTA, 1 mM sodium orthovanadate, 10 mM sodium b-glycerophosphate, 50 mM sodium fluoride, 5 mM sodium pyrophosphate, and 1% NP-40) supplemented with 1 × cOmplete protease inhibitor cocktail (Roche, Basel, Switzerland) and 1 mM DTT. Cell extracts were transferred to Eppendorf tubes and clarified by centrifugation at 14,000 rpm for 15 min at 4 °C. Protein concentration was measured by Bradford assay. For SDS-PAGE analysis, samples were prepared to an equal protein concentration by normalizing them with NP-40 lysis buffer and 1X LDS sample buffer supplemented with 2.5% β-mercaptoethanol. Samples were boiled at 95 °C for 5 min before SDS-PAGE. 

#### 3.3.2. SDS-PAGE and Immunoblotting

Cell extracts (20 µg protein) were loaded into 8% polyacrylamide gels or precast 4–20% NuPAGE™ Midi Bis-Tris gels (Invitrogen, Darmstadt, Germany) and resolved at 120 V for 90–120 min. Protein was then transferred to a nitrocellulose membrane. Membranes were blocked in 5% (*w*/*v*) non-fat milk (Marvel, Dublin, Ireland) in TBS-T (50 mM Tris–HCl pH 7.5, 150 mM NaCl, 0.2% Tween 20) for 1 h and incubated overnight at 4 °C with the appropriate primary antibodies, also diluted in 5 mL TBS-T/5% non-fat milk. Membranes were then washed for 3 × 10 min using TBS-T in a shaking platform. They were then incubated with HRP-conjugated secondary antibodies diluted in 5% non-fat milk + TBS-T for 1 h at room temperature, after which they were washed for 3 × 10 min in TBST-T. Signal detection was performed using an enhanced chemiluminescence (ECL) system and data obtained using the ChemiDoc Imaging System (Bio-Rad, Hercules, CA, USA). The primary antibodies used were the following: CK1δ (MRC PPU; SA609); CK1α (MRC PPU; SA527); CK1ε (MRC PPU; SA610); DVL3 (ProteinTech, Manchester, UK; 13444 1-AP); pVANGL2 (ABclonal Technology, Woburn, UK; AP1206) Vinculin (CST; 4650); GAPDH (ProteinTech, Manchester, UK; 60004-1Ig); GAPDH-HRP (ProteinTech; HRP-60004); SACK1A (MRC PPU; S911D); SACK1B (MRC PPU; SA270); SACK1E (MRC PPU; SA272); SACK1H (MRC PPU; SA273); SACK1G (Abcam; Ab121750); CUL2 (Invitrogen, Darmstadt, Germany; 51-8100); ubiquitin (Abcam; Ab19247); Cereblon (CST; 60312/71810); Goat anti-rabbit IgG (7074, CST, 1:2500); and Rabbit anti-sheep IgG (31480, Thermo Fisher Scientific, Darmstadt, Germany, 1:2500).

#### 3.3.3. In Vitro Kinase Assays for IC_50_ Determination

In vitro kinase assays were performed by MRC PPU International Centre for Kinase Profiling (https://www.kinase-screen.mrc.ac.uk/services/ic50, accessed on 1 August 2025). CK1α or δ (5 mU diluted in 20 mM HEPES pH7.5, 0.15 M NaCl, 0.1 mM EGTA, 0.1% Triton X-100, 5 mM DTT, 50% glycerol) was assayed against a CK1 substrate peptide (RRKDLHDDEEDEAMSITA) in a final volume of 25.5 µL containing 20 mM HEPES pH 7.5, 0.15 M NaCl, 0.1 mM EDTA, 5 mM DTT, 0.1% Triton-X 100, CK1 peptide (0.5 mM), 10 mM magnesium acetate, indicated concentrations of compounds or DMSO control, and 0.02 mM [^33P-γ^ATP] (500–1000 cpm/pmole) and incubated for 30 min at room temperature. Assays were stopped by the addition of 5 µL of 0.5 M (3%) orthophosphoric acid and then harvested onto P81 Unifilter plates with a wash buffer of 50 mM orthophosphoric acid. ^33^P-radioactivity (cpm) for the kinase assay conducted in the presence of the DMSO control was assigned 100% CK1 activity.

## 4. Conclusions

In this study, we established a flexible synthetic platform for the modular preparation of designed CK1δ-PROTACs following the ternary scheme CK1δ/ligand/linker/E3 ligase ligand. We applied this platform to generate two different sets of projected PROTAC degrader compounds towards CK1δ. The synthetic route previously published by our group in 2019 was applied and extended to generate an inhibitor compound (**I9**) containing an aniline moiety, facilitating linker attachment via amide coupling. All dimeric intermediate compounds (**L6a–d**, **L8a–d**) were generated in sufficient quantities to be employed in the final amide coupling in order to generate the PROTAC compounds.

While alkyl-linker PROTAC compounds **P1a–d** (three through six carbon atoms, respectively) were synthesized successfully with yields in the range from 6 to 22%, compounds **P2b** and **P2c** (four and five carbon atoms) of this series could not be generated by the method. Despite our efforts towards expanded variations regarding solvent, reaction time, temperature, and coupling reagents, all attempts were ineffective in yielding the desired compounds **P2b** and **P2c**.

The set of PROTACs containing PEG linker motifs, however, were synthesized successfully, possibly due to their length and higher conformational flexibility compared to alkyl linkers. Overall, we were successful in establishing a versatile and effective synthetic platform for the systematic synthesis of various PROTAC candidate compounds.

We were able to apply all our PROTAC candidate molecules in various cells to assess the potential degradation of CK1 isoforms, their interacting partners, and the impact on the phosphorylation of downstream substrates. A few PROTACs induced a dose-dependent degradation of CK1δ and CK1ε but not the related kinase CK1α. **P1d** was the most potent CK1δ/ε degrader, robustly degrading these kinases at concentrations of 5 μM and 10 μM within 6 h in multiple cell lines, including U2OS and A549. We established that **P1d** degrades CK1δ/ε via the CUL4^CRBN^ E3 ligase complex and the proteasome.

A key challenge in utilizing kinase inhibitors as warheads for PROTACs is that the PROTACs could still act as inhibitors even in the absence of degradation, and the effects of PROTACs on downstream biology might be due to inhibition, without the added benefit of degradation. Therefore, it is important to evaluate the relative contribution of inhibition vs. degradation on downstream biology, as well as the potency of kinase inhibition in vitro. For our top PROTAC **P1d**, an in vitro kinase inhibition assay against CK1δ carried out in parallel with the inhibitor I9 showed IC_50_ values of 2.16 μM compared to 3.43 μM for I9. These data suggested the potential of **P1d** to be a more potent cellular inhibitor of CK1δ/ε than I9. However, the superiority of the **P1d** PROTAC at inhibiting downstream CK1δ/ε via degradation compared to inhibition comes from the observations when WT and CRBN-KO U2OS cells were treated with **P1d**. Here, **P1d** degrades CK1δ/ε in WT cells but not in CRBN-KO U2OS cells; however, it should still be able to inhibit CK1δ/ε activity in CRBN-KO cells. Excitingly, the **P1d**-induced inhibition of both DVL3 and VANGL2 phosphorylation at both 5 μM and 10 μM in WT U2OS cells is much more potent than in CRBN-KO U2OS cells, suggesting that it is indeed the degradation of CK1δ/ε that accounts for the potent inhibition of downstream signalling. Nonetheless, 10 μM of **P1d** still potently inhibits DVL3 and VANGL2 phosphorylation in CRBN-KO U2OS cells, implying that the compound enters the cells and inhibits CK1δ/ε, as no CK1δ/ε degradation is seen in CRBN-KO U2OS cells. Another exciting observation we made is that in A549 cells, **P1d** induced the degradation of SACK1E (formerly FAM83E) but no other SACK1 proteins, although the functional significance of this needs further investigation. In cells it is known that CK1δ/ε exist in complex with SACK1A/FAM83A, SACK1B/FAM83B, SACK1E/FAM83E, and SACK1H/FAM83H, but the function of SACK1E- CK1δ/ε complexes is unknown [23].

The current minimum effective dose of **P1d** of 5 μM required to degrade CK1δ/ε does not compare favourably with more potent PROTACs against other targets that work at low nanomolar concentrations. This limitation is most likely due to the utilized CK1δ inhibitor moiety, which inhibits CK1δ in vitro with an IC_50_ of 3.43 μM. We aim to develop new series of compounds with a more potent inhibitor warhead and alternative linkers as well as other E3 ligands, including those recruiting VHL, KLHDC2, and cIAP2. Based on the established procedures providing dimers containing E3 ligase ligands and linkers of varying motifs and lengths readily available, we aim to apply the modular platform for the preparation of further PROTAC sets towards various proteins of interest.

## Figures and Tables

**Table 1:** Conditions employed in order to try to generate compounds **P2b–c**.

I9	L8b–c	Solvent	Base (5 equiv.)	Coupling Reagents	Temperature [°C]	Reaction Time	Additive
1 equiv.	1 equiv.	MeCN	DIPEA	EDC, HOBT, DMAP	40 °C	48 h	-
1 equiv.	1 equiv.	DMSO	DIPEA	EDC, HOBT, DMAP	40 °C	48 h	-
1 equiv.	1 equiv.	DMF	DIPEA	EDC, HOBT, DMAP	40 °C	48 h	-
1 equiv.	1 equiv.	THF	DIPEA	EDC, HOBT, DMAP	40 °C	48 h	-
1 equiv.	1 equiv.	MeCN	DIPEA	EDC, HOBT, DMAP	25 °C	48 h	-
1 equiv.	1 equiv.	MeCN	Potassium carbonate	EDC, HOBT, DMAP	40 °C	48 h	-
1 equiv.	1 equiv.	MeCN	DIPEA	EDC, HOBT, DMAP	40 °C	48 h	-
1 equiv.	1 equiv.	MeCN	TEA	EDC, HOBT, DMAP	40 °C	48 h	-
1 equiv.	1 equiv.	MeCN	DBU	EDC, HOBT, DMAP	40 °C	48 h	-
1 equiv.	1 equiv.	MeCN	DIPEA	EDC, HOBT, DMAP	40 °C	48 h	Mole sieve (3 Å)
2 equiv.	1 equiv.	MeCN	DIPEA	EDC, HOBT, DMAP	40 °C	48 h	-
1 equiv.	2 equiv.	MeCN	DIPEA	EDC, HOBT, DMAP	40 °C	48 h	-
1 equiv.	1 equiv.	MeCN	DIPEA	EDC, HOBT, DMAP	40 °C	48 h	Glutarimide (5 equiv.)
1 equiv.	1 equiv.	MeCN	DIPEA	EDC, HOBT, DMAP	40 °C	48 h	Glutarimide (10 equiv.)
1 equiv.	1 equiv.	MeCN	DIPEA	HATU	40 °C	5 h	-
1 equiv.	1 equiv.	MeCN	Potassium carbonate	HATU	40 °C	5 h	-
1 equiv.	1 equiv.	MeCN	TEA	HATU	40 °C	5 h	-
1 equiv.	1 equiv.	MeCN	DBU	HATU	40 °C	5 h	-
1 equiv.	1 equiv.	MeCN	DIPEA	HATU	40 °C	5 h	Mole sieve (3 Å)
1 equiv.	1 equiv.	MeCN	DIPEA	HATU	70 °C	5 h	-

**Table 2:** Overview of compounds and corresponding linker lengths.

PROTAC	Linker Length (Å) ^1^
**P1a**	4.179
**P1b**	4.991
**P1c**	6.428
**P1d**	7.413
**P2a**	4.179
**P2d**	7.413
**P3a**	7.103
**P3b**	10.712
**P3c**	14.062
**P3d**	8.492
**P4a**	7.103
**P4b**	10.712
**P4c**	14.062
**P4d**	8.492

^1^ Measured linker lengths between the amide nitrogen connecting the linker to the inhibitor and the nitrogen or oxygen atom linking the linker to the thalidomide derivative. Linker lengths were measured based on 3D molecular models of the corresponding PROTACs, constructed and energy-minimized using Chem3D Pro 12.0.

## Data Availability

The original contributions presented in this study are included in the article/Supplementary Materials. Further inquiries can be directed to the corresponding author(s).

## Supplementary Materials

The following supporting information can be downloaded at https://www.mdpi.com/article/10.3390/molecules30224452/s1, Scheme S1. Schematic synthesis routes resulting in the desired intermediate compounds containing a glycol linker attached to the thalidomide derivative. The route employing the iodinated linker intermediates lead to resulted in very low yields as well as dual linker substitution. The route employing the tosylated linker intermediates resulted in much better yields, while keeping dual substitution to a minimum. (a) 1.0 equiv. *tert*-butyl acrylate and 0.04 equiv. Triton B solution (40% in water), rt, 120 h; (b) 1.2 equiv. triphenylphosphine, 1.2 equiv. imidazole and 1.2 equiv. iodine in THF, rt. 2 h; (c) 3.0 equiv. caesium carbonate in DMF, rt, 16 h. (d) 1.0 equiv. *tert*-butyl acrylate and 0.04 equiv. Triton B solution (40% in water), rt, 120 h; (e) 1.5 equiv. tosyl chloride, 3.0 equiv. TEA and 0.1 equiv. DMAP in CH2Cl2, rt. 24 h; (f) 1.5 equiv. potassium bicarbonate and 0.1 equiv. sodium iodide in DMF, 80 °C, 16 h. Figures S1–S14: ^1^H- and ^13^C-NMR spectra for the PROTAC compounds; Figures S15–S28: HPLC and MS spectra for the PROTAC compounds.

## Abbreviations

CK1	Casein Kinase 1
PROTAC	Proteolysis Targeting Chimera
PEG	Polyethylene Glycol
MOA	Mode of Action
SMI	Small Molecule Inhibitor
POI	Protein of Interest
UPS	Ubiquitin Proteasome System
APC	Adenomatous Polyposis Coli
AXIN	Axis Inhibition Protein
GSK3 protein	Glycogen Synthase Kinase 3 Protein
HRII	Hydrophobic Region II
ATP	Adenosine Triphosphate
TPD	Targeted Protein Degradation
IWP	Inhibitor of Wnt Production
CRBN	Cereblon
p-MeO	Para-Methoxy
SAR	Structure–Activity Relationship
TEA	Triethylamine
DMSO	Dimethylsulfoxide
DMF	Dimethylformamide
equiv.	Equivalent
TFA	Trifluororacetic Acid
HATU	Hexafluorophosphate Azabenzotriazole Tetramethyl Uronium
DIPEA	N, N-Diisopropylethylamine
EDC	1-Ethyl-3-(3-dimethylaminopropyl) Carbodiimide
HOBt	Hydroxybenzotriazole
DMAP	4-Dimethylaminopyridine
MeCN	Acetonitrile
RP-HPLC	Reversed-Phase High-Performance Liquid Chromatography
MS	Mass Spectrometry
HR-MS	High-Resolution Mass Spectrometry
NMR	Nuclear Magnetic Resonance
EA	Ethyl Acetate
MeOH	Methanol

## References

[1] Békés M., Langley D.R., Crews C.M. (2022). PROTAC targeted protein degraders: The past is prologue. Nat. Rev. Drug Discov..

[2] Li K., Crews C.M. (2022). PROTACs: Past, present and future. Chem. Soc. Rev..

[3] Nalawansha D.A., Crews C.M. (2020). PROTACs: An Emerging Therapeutic Modality in Precision Medicine. Cell Chem. Biol..

[4] Hershko A., Ciechanover A. (1998). The ubiquitin system. Annu. Rev. Biochem..

[5] Kleiger G., Mayor T. (2014). Perilous journey: A tour of the ubiquitin-proteasome system. Trends Cell Biol..

[6] Nandi D., Tahiliani P., Kumar A., Chandu D. (2006). The ubiquitin-proteasome system. J. Biosci..

[7] Paiva S.-L., Da Silva S.R., de Araujo E.D., Gunning P.T. (2018). Regulating the Master Regulator: Controlling Ubiquitination by Thinking Outside the Active Site. J. Med. Chem..

[8] Popovic D., Vucic D., Dikic I. (2014). Ubiquitination in disease pathogenesis and treatment. Nat. Med..

[9] Jiang Y., Beaudet A.L. (2004). Human disorders of ubiquitination and proteasomal degradation. Curr. Opin. Pediatr..

[10] Paiva S.-L., Crews C.M. (2019). Targeted protein degradation: Elements of PROTAC design. Curr. Opin. Chem. Biol..

[11] An S., Fu L. (2018). Small-molecule PROTACs: An emerging and promising approach for the development of targeted therapy drugs. EBioMedicine.

[12] Berndsen C.E., Wolberger C. (2014). New insights into ubiquitin E3 ligase mechanism. Nat. Struct. Mol. Biol..

[13] Schreiber A., Peter M. (2014). Substrate recognition in selective autophagy and the ubiquitin-proteasome system. Biochim. Biophys. Acta.

[14] Alabi S.B., Crews C.M. (2021). Major advances in targeted protein degradation: PROTACs, LYTACs, and MADTACs. J. Biol. Chem..

[15] Bondeson D.P., Mares A., Smith I.E.D., Ko E., Campos S., Miah A.H., Mulholland K.E., Routly N., Buckley D.L., Gustafson J.L. (2015). Catalytic in vivo protein knockdown by small-molecule PROTACs. Nat. Chem. Biol..

[16] Zagidullin A., Milyukov V., Rizvanov A., Bulatov E. (2020). Novel approaches for the rational design of PROTAC linkers. Explor. Target. Anti-Tumor Ther..

[17] Troup R.I., Fallan C., Baud M.G.J. (2020). Current strategies for the design of PROTAC linkers: A critical review. Explor. Target. Anti-Tumor Ther..

[18] Poongavanam V., Peintner S., Abeje Y., Kölling F., Meibom D., Erdelyi M., Kihlberg J. (2025). Linker-Determined Folding and Hydrophobic Interactions Explain a Major Difference in PROTAC Cell Permeability. ACS Med. Chem. Lett..

[19] Cyrus K., Wehenkel M., Choi E.-Y., Han H.-J., Lee H., Swanson H., Kim K.-B. (2011). Impact of linker length on the activity of PROTACs. Mol. Biosyst..

[20] Knippschild U., Krüger M., Richter J., Xu P., García-Reyes B., Peifer C., Halekotte J., Bakulev V., Bischof J. (2014). The CK1 Family: Contribution to Cellular Stress Response and Its Role in Carcinogenesis. Front. Oncol..

[21] Narasimamurthy R., Hunt S.R., Lu Y., Fustin J.-M., Okamura H., Partch C.L., Forger D.B., Kim J.K., Virshup D.M. (2018). CK1δ/ε protein kinase primes the PER2 circadian phosphoswitch. Proc. Natl. Acad. Sci. USA.

[22] Sinnberg T., Wang J., Sauer B., Schittek B. (2016). Casein kinase 1α has a non-redundant and dominant role within the CK1 family in melanoma progression. BMC Cancer.

[23] Fulcher L.J., Sapkota G.P. (2020). Functions and regulation of the serine/threonine protein kinase CK1 family: Moving beyond promiscuity. Biochem. J..

[24] Gybeľ T., Čada Š., Klementová D., Schwalm M.P., Berger B.-T., Šebesta M., Knapp S., Bryja V. (2024). Splice variants of CK1α and CK1α-like: Comparative analysis of subcellular localization, kinase activity, and function in the Wnt signaling pathway. J. Biol. Chem..

[25] Liu J., Xiao Q., Xiao J., Niu C., Li Y., Zhang X., Zhou Z., Shu G., Yin G. (2022). Wnt/β-catenin signalling: Function, biological mechanisms, and therapeutic opportunities. Signal Transduct. Target. Ther..

[26] MacDonald B.T., Tamai K., He X. (2009). Wnt/beta-catenin signaling: Components, mechanisms, and diseases. Dev. Cell.

[27] Bryja V., Bernatík O. (2014). Dishevelled at the Crossroads of Pathways. Wnt Signaling in Development and Disease: Molecular Mechanisms and Biological Functions.

[28] Xu P., Ianes C., Gärtner F., Liu C., Burster T., Bakulev V., Rachidi N., Knippschild U., Bischof J. (2019). Structure, regulation, and (patho-)physiological functions of the stress-induced protein kinase CK1 delta (CSNK1D). Gene.

[29] Mazzoldi E.L., Pastò A., Ceppelli E., Pilotto G., Barbieri V., Amadori A., Pavan S. (2019). Casein Kinase 1 Delta Regulates Cell Proliferation, Response to Chemotherapy and Migration in Human Ovarian Cancer Cells. Front. Oncol..

[30] Cheong J.K., Virshup D.M. (2011). Casein kinase 1: Complexity in the family. Int. J. Biochem. Cell Biol..

[31] Nishiguchi G., Caine E.A., McGowan K., Shi Z., Aggarwal A., Mayasundari A., Price J., Yang L., Li Y., Fu X. (2024). Structure–Activity Relationship of Potent, Selective, and Orally Bioavailable Molecular Glue Degraders of CK1α. ACS Med. Chem. Lett..

[32] MedchemExpress.com. https://www.medchemexpress.com/ck1%CE%B1-degrader-1.html.

[33] Wang K., Jiang M., Liu H., Meng C., Li M., Lu H. (2024). Discovery of novel co-degradation CK1α and CDK7/9 PROTACs with p53 activation for treating acute myeloid leukemia. Bioorganic Chem..

[34] Haag A., Němec V., Janovská P., Bartošíková J., Adhikari B., Müller J., Schwalm M.P., Čada Š., Ohmayer U., Daub H. (2025). Development and Discovery of a Selective Degrader of Casein Kinases 1 δ/ε. J. Med. Chem..

[35] García-Reyes B., Witt L., Jansen B., Karasu E., Gehring T., Leban J., Henne-Bruns D., Pichlo C., Brunstein E., Baumann U. (2018). Discovery of Inhibitor of Wnt Production 2 (IWP-2) and Related Compounds As Selective ATP-Competitive Inhibitors of Casein Kinase 1 (CK1) δ/ε. J. Med. Chem..

[36] Liu C., Witt L., Ianes C., Bischof J., Bammert M.-T., Baier J., Kirschner S., Henne-Bruns D., Xu P., Kornmann M. (2019). Newly Developed CK1-Specific Inhibitors Show Specifically Stronger Effects on CK1 Mutants and Colon Cancer Cell Lines. Int. J. Mol. Sci..

[37] Schade D., Plowright A.T. (2015). Medicinal Chemistry Approaches to Heart Regeneration. J. Med. Chem..

[38] Chen B., Dodge M.E., Tang W., Lu J., Ma Z., Fan C.-W., Wei S., Hao W., Kilgore J., Williams N.S. (2009). Small molecule-mediated disruption of Wnt-dependent signaling in tissue regeneration and cancer. Nat. Chem. Biol..

[39] Bischof J., Leban J., Zaja M., Grothey A., Radunsky B., Othersen O., Strobl S., Vitt D., Knippschild U. (2012). 2-Benzamido-N-(1H-benzodimidazol-2-yl)thiazole-4-carboxamide derivatives as potent inhibitors of CK1δ/ε. Amino Acids.

[40] Abeje Y.E., Wieske L.H.E., Poongavanam V., Maassen S., Atilaw Y., Cromm P., Lehmann L., Erdelyi M., Meibom D., Kihlberg J. (2025). Impact of Linker Composition on VHL PROTAC Cell Permeability. J. Med. Chem..

[41] Petzold G., Fischer E.S., Thomä N.H. (2016). Structural basis of lenalidomide-induced CK1α degradation by the CRL4(CRBN) ubiquitin ligase. Nature.

[42] Hernández-Núñez E., Tlahuext H., Moo-Puc R., Torres-Gómez H., Reyes-Martínez R., Cedillo-Rivera R., Nava-Zuazo C., Navarrete-Vazquez G. (2009). Synthesis and in vitro trichomonicidal, giardicidal and amebicidal activity of N-acetamide(sulfonamide)-2-methyl-4-nitro-1H-imidazoles. Eur. J. Med. Chem..

[43] Roopan S.M., Khan F.R.N., Mandal B.K. (2010). Fe nano particles mediated C–N bond-forming reaction: Regioselective synthesis of 3-[(2-chloroquinolin-3-yl)methyl]pyrimidin-4(3H)ones. Tetrahedron Lett..

[44] Xu J., Yadan J.-C. (1995). A New and Convenient Synthesis of Imidazole-2-thiones from Imidazoles. Synlett.

[45] Schmuck C., Rehm T., Geiger L., Schäfer M. (2007). Synthesis and self-association properties of flexible guanidiniocarbonylpyrrole-carboxylate zwitterions in DMSO: Intra- versus intermolecular ion pairing. J. Org. Chem..

[46] Kim K., Lee D.H., Park S., Jo S.-H., Ku B., Park S.G., Park B.C., Jeon Y.U., Ahn S., Kang C.H. (2019). Disordered region of cereblon is required for efficient degradation by proteolysis-targeting chimera. Sci. Rep..

[47] Ghosh A.K., Shahabi D. (2021). Synthesis of amide derivatives for electron deficient amines and functionalized carboxylic acids using EDC and DMAP and a catalytic amount of HOBt as the coupling reagents. Tetrahedron Lett..

[48] Cromm P.M., Samarasinghe K.T.G., Hines J., Crews C.M. (2018). Addressing Kinase-Independent Functions of Fak via PROTAC-Mediated Degradation. J. Am. Chem. Soc..

[49] Kaucká M., Petersen J., Janovská P., Radaszkiewicz T., Smyčková L., Daulat A.M., Borg J.-P., Schulte G., Bryja V. (2015). Asymmetry of VANGL2 in migrating lymphocytes as a tool to monitor activity of the mammalian WNT/planar cell polarity pathway. Cell Commun. Signal. CCS.

[50] Yang W., Garrett L., Feng D., Elliott G., Liu X., Wang N., Wong Y.M., Choi N.T., Yang Y., Gao B. (2017). Wnt-induced Vangl2 phosphorylation is dose-dependently required for planar cell polarity in mammalian development. Cell Res..

[51] Fulcher L.J., Bozatzi P., Tachie-Menson T., Wu K.Z.L., Cummins T.D., Bufton J.C., Pinkas D.M., Dunbar K., Shrestha S., Wood N.T. (2018). The DUF1669 domain of FAM83 family proteins anchor casein kinase 1 isoforms. Sci. Signal..

[52] Glennie L., Curnutt N., Cartwright T., Dunbar K., Le Chatelier B., Wood N.T., Macartney T.J., Woo C.M., Sapkota G.P. (2025). The contribution of native protein complexes to targeted protein degradation. BioRxiv.

[53] Sastry G.M., Adzhigirey M., Day T., Annabhimoju R., Sherman W. (2013). Protein and ligand preparation: Parameters, protocols, and influence on virtual screening enrichments. J. Comput.-Aided Mol. Des..

[54] Mohamadi F., Richards N.G.J., Guida W.C., Liskamp R., Lipton M., Caufield C., Chang G., Hendrickson T., Still W.C. (1990). Macromodel—An integrated software system for moedling organic and bioorganic molecules using molecular mechanics. J. Comput. Chem..

[55] Friesner R.A., Banks J.L., Murphy R.B., Halgren T.A., Klicic J.J., Mainz D.T., Repasky M.P., Knoll E.H., Shelley M., Perry J.K. (2004). Glide: A New Approach for Rapid, Accurate Docking and Scoring. 1. Method and Assessment of Docking Accuracy. J. Med. Chem..

[56] Dunbar K., Jones R.A., Dingwell K., Macartney T.J., Smith J.C., Sapkota G.P. (2021). FAM83F regulates canonical Wnt signalling through an interaction with CK1α. Life Sci. Alliance.

