# Supplementary material for: Design and Synthesis of Novel Candidate CK1δ Proteolysis Targeting Chimeras (PROTACs)
Source: Molecules. 2025 Nov 18;30(22):4452. doi: 10.3390/molecules30224452 (PMC12655757; doi:10.3390/molecules30224452)
Supplement: Supplementary file 1 [file molecules-30-04452-s001.zip › molecules-3981189-supplementary.pdf]

Supporting Information

Design and Synthesis of Novel Candidate CK1δ Proteolysis Targeting Chimeras (PROTACs)

Malte Arnold[1], Temi Thompson[2], Lorraine Glennie[2], Mattes Hollnagel[1], Gopal Sapkota[2], Christian Peifer[1,*]

[1]Department of Pharmaceutical and Medicinal Chemistry, University of Kiel, Kiel, Germany

[2]MRC PPU, Sir James Black Centre, School of Life Sciences, University of Dundee, Dundee, Scotland, UK

*Corresponding author: Christian Peifer, Email: cpeifer@pharmazie.uni-kiel.de

Table of contents

Spectroscopic data 3
Copies of NMR spectra of synthesized PROTAC compounds 12
Compound P1a 12
Compound P1b 13
Compound P1c 14
Compound P1d 15
Compound P2a 16
Compound P2d 17
Compound P3a 18
Compound P3b 19
Compound P3c 20
Compound P3d 21
Compound P4a 22
Compound P4b 23
Compound P4c 24
Compound P4d 25
HPLC chromatograms and MS spectra of synthesized PROTAC compounds 26
Compound P1a 26
Compound P1b 27
Compound P1c 28
Compound P1d 29
Compound P2a 30
Compound P2d 31
Compound P3a 32
Compound P3b 33
Compound P3c 34
Compound P3d 35
Compound P4a 36
Compound P4b 37
Compound P4c 38
Compound P4d 39

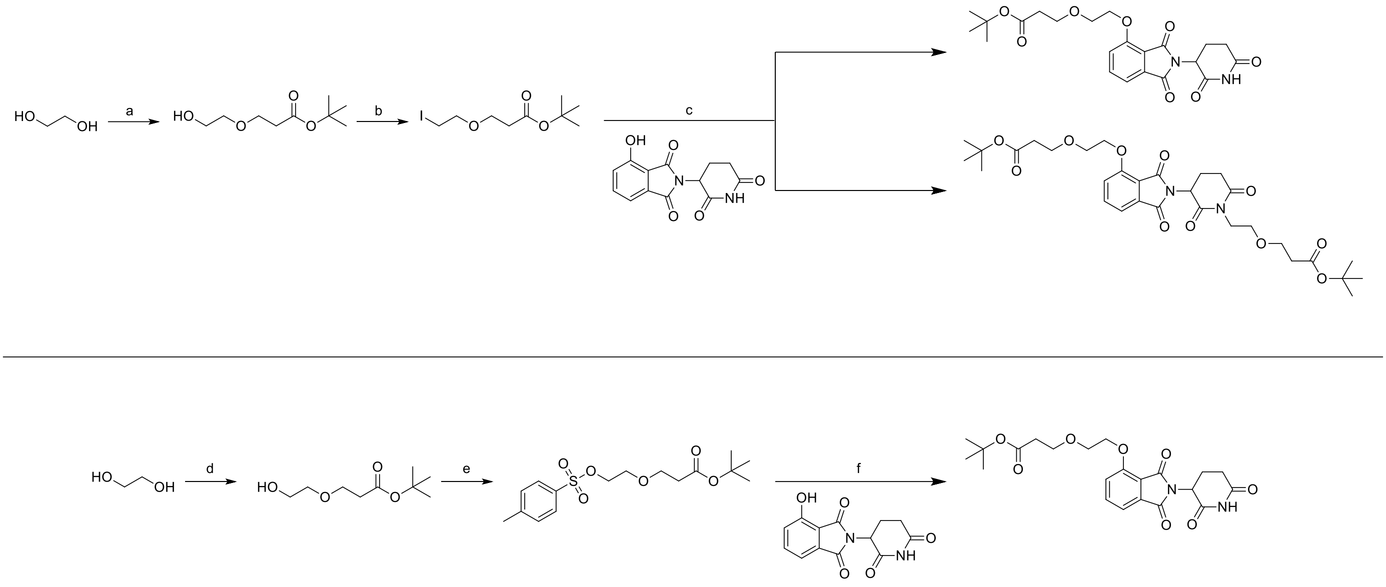

Scheme S1. Schematic synthesis routes resulting in the desired intermediate compounds containing a glycol linker attached to the thalidomide derivative. The route employing the iodinated linker intermediates lead to resulted in very low yields as well as dual linker substitution. The route employing the tosylated linker intermediates resulted in much better yields, while keeping dual substitution to a minimum. (**a**) 1.0 equiv. *tert*-butyl acrylate and 0.04 equiv. Triton B solution (40% in water), rt, 120 h; (**b**) 1.2 equiv. triphenylphosphine, 1.2 equiv. imidazole and 1.2 equiv. iodine in THF, rt. 2 h; (**c**) 3.0 equiv. caesium carbonate in DMF, rt, 16 h. (**d**) 1.0 equiv. *tert*-butyl acrylate and 0.04 equiv. Triton B solution (40% in water), rt, 120 h; (**e**) 1.5 equiv. tosyl chloride, 3.0 equiv. TEA and 0.1 equiv. DMAP in CH_2Cl_2, rt. 24 h; (**f**) 1.5 equiv. potassium bicarbonate and 0.1 equiv. sodium iodide in DMF, 80 °C, 16 h.

Spectroscopic data

2-Chloro-N-(6-(trifluoromethyl)benzo[d]thiazol-2-yl)acetamide (I3)

2-Amino-6-(trifluoromethyl) benzothiazole (**I2**, 1.00 equiv, 16.97 mmol. 3.70 g) and TEA (1.10 equiv, 18.66 mmol, 1.89 g) are dissolved in 5 mL dichloromethane. Chloroacetyl chloride (**I1**, 1.10 equiv, 18.66 mmol, 2.11 g) is dissolved in 5 mL dichloromethane and added to the reaction mixture dropwise over an hour. The reaction mixture is left stirring at RT overnight. The solvent is evaporated to afford the product, which is dissolved in ethyl acetate, washed with water and concentrated in vacuo again. The product is recrystallized (ethanol/water 1:1) to afford it as yellow needles (90.0 % yield; 1H NMR (300 MHz, DMSO-d6): δ = 12.96 (s, 1 H), 8.52 (s, 1 H), 7.94 (d, J = 8.5 Hz, 1 H), 7.75 (dd, J = 8.5 Hz, 1.6 Hz, 1 H), 4.50 (s, 2 H) ppm; 13C NMR (75.5 MHz, DMSO-d6): δ = 166.9, 161.3, 151.7, 132.5, 124.4 (d, *J* = 8.5 Hz), 123.6, 123.5, 121.7, 120.5, 43.0 ppm; HPLC: 8.9 min, start at 3 % B; MS calcd. m/z 293.98, found m/z 294.8 [M+H]+).

3-(4-Nitrobenzyl)pyrimidin-4(3H)-one (I6)

KOH (1.5 equiv, 32.44 mmol, 1.82 g) is ground, weighed in and left under vacuum for 45 min at RT. The system is flooded with argon afterwards. KOH is dissolved in 9.5 mL DMSO while stirring at 110 °C. 4(3H)-Pyrimidinone (**I4**, 1.00 equiv, 21.63 mmol, 2.08 g) and 4-Nitrobenzyl chloride (**I5**, 1.10 equiv, 23.79 mmol, 4.08 g) are dissolved in 10 mL DMSO and added to the reaction mixture via injection. Iron (nano) (0.10 equiv, 2.16 mmol, 121 mg) is suspended in 0.5 mL DMSO and added via injection, afterwards the reaction mixture is left stirring at 110 °C for 2 h. After cooling down, the reaction mixture is decanted on ice and, if needed, neutralized with a few drops of 1M HCl. The aqueous layer is extracted with DCM [3x 30 mL]. The organic layers are combined, washed with water [3x 30 mL] and dried over sodium sulfate. The solvent is evaporated and the crude product is purified via flash chromatography (silica gel, petroleum ether/ethyl acetate 80/20 – 0/100) to afford the product as an orange-brown solid (55.4 % yield; 1H NMR (300 MHz, DMSO-d6): δ = 8.71 (s, 1 H), 8.21 (dt, J = 8.8 Hz, 2.0 Hz, 2 H), 7.96 (d, J = 6.6 Hz,1 H), 7.56 (d, J = 8.9 Hz, 2 H), 6.45 (dd, J = 6.8 Hz, 0.9 Hz, 1 H), 5.23 (s, 2 H) ppm; 13C NMR (75.5 MHz, DMSO-d6): δ = 160.5, 154.4, 153.4, 147.4, 144.4, 129.2, 124.3, 115.8, 49.2 ppm; HPLC: 6.1 min, start at 3 % B; MS calcd. m/z 231.06, found m/z 231.8 [M+H]+).

3-(4-Nitrobenzyl)-2-thioxo-2,3-dihydropyrimidin-4(1H)-one (I7)

Intermediate I6 (1.00 equiv, 11.94 mmol, 2.76 g) and sodium bicarbonate (1.10 equiv, 13.13 mmol, 1.10 g) are dissolved in 30 mL EA/H2O 1:1. O-Phenyl chlorothionoformate (2.50 equiv, 29.84 mmol, 5.15 g) is added dropwise and the reaction mixture is left stirring overnight afterwards. The aqueous layer is extracted with EA (3x 15 mL). The organic layers are combined, washed with brine (3x 15 mL) and dried over sodium sulfate. The solvent is evaporated and the isolated intermediate stage is dissolved in 15 mL MeOH. TEA (6.00 equiv, 71.62 mmol, 7.25 g) is added and the reaction mixture is left stirring at 80 °C for 4h. The solvent is evaporated and the crude product is first purified via flash chromatography (dichloromethane/methyl alcohol). The crude is then suspended in ethyl acetate and filtered. The residue is dissolved in dichloromethane and the solvent is evaporated to afford the product as an orange solid (31.6 % yield; 1H NMR (300 MHz, DMSO-d6): δ = 12.76 (s, 1 H), 8.17 (d, J = 8.3 Hz, 2 H), 7.54 (d, J = 7.8 Hz, 1 H), 7.51 (d, J = 8.3 Hz, 2 H), 6.03 (d, J = 7.4 Hz, 1 H), 5.61 (s, 2 H) ppm; 13C NMR (75.5 MHz, DMSO-d6): δ = 177.5, 160.8, 147.0, 144.7, 141.8, 128.6, 124.0, 104.7, 48.4 ppm; HPLC: 7.5 min, start at 3 % B; MS calcd. m/z 263.04, found m/z 263.8 [M+H]+).

2-((1-(4-Nitrobenzyl)-6-oxo-1,6-dihydropyrimidin-2-yl)thio)-N-(6-(trifluoromethyl)benzo[d]thiazol-2-yl)acetamide (I8)

Intermediate I7 (1.00 equiv, 1.70 mmol, 500 mg) and intermediate I8 (1.00 equiv, 1.70 mmol, 447 mg) are dissolved in 10 mL DMF. TEA (3.00 equiv, 5.09 mmol, 515 mg) is added via injection and the reaction mixture is left stirring at 80 °C for 2h. After cooling down, the reaction is quenched with water and extracted with EA (3x 15 mL). The organic layers are combined, washed with brine (2x 15 mL) and water (1x 15 mL) and dried with sodium sulfate. The solvent is evaporated and the crude product purified via flash chromatography (silica gel, petroleum ether/ethyl acetate 80/20 – 0/100) to afford a yellow solid (91.3 % yield; 1H NMR (300 MHz, DMSO-d6): δ = 12.92 (s, 1 H), 8.49 (s, 1 H), 8.24 (dt, J = 8.8 Hz, 2.1 Hz, 2 H), 7.92 (d, J = 8.5 Hz, 1 H), 7.84 (d, J = 6.5 Hz, 1 H), 7.78-7.73 (m, 1 H), 7.54 (d, J = 8.8 Hz, 2 H), 6.30 (d, J = 6.5 Hz, 1 H), 5.42 (s, 2 H), 4.34 (s, 2 H) ppm; 13C NMR (75.5 MHz, DMSO-d6): δ = 167.6, 162.5, 161.5, 161.3, 152.8, 151.8, 147.4, 143.2, 132.5, 128.5, 124.4, 124.3 (d, *J* = 31.5 Hz), 123.7, 123.5, 121.8, 120.5, 110.8, 47.1, 36.4 ppm; HPLC: 9.6 min, start at 3 % B; MS calcd. m/z 521.04, found m/z 519.9 [M-H]-).

2-((1-(4-Aminobenzyl)-6-oxo-1,6-dihydropyrimidin-2-yl)thio)-N-(6-(trifluoromethyl)benzo[d]thiazol-2-yl)acetamide (I9)

Intermediate I8 (1061 mg, 2.0 mmol) is dissolved in 20 mL methyl alcohol. Palladium on activated carbon (10 %, 0.1 equiv) is added to the reaction mixture. The atmosphere is exchanged for hydrogen (3 bar). The reaction is stirred at room temperature for 48 h. Afterwards the catalyst is filtered off through a Celite pad, which is washed with methyl alcohol (3x 20 mL). The filtrate and washings are combined, the solvent is evaporated and the crude product is purified via flash chromatography (silica gel, dichloromethane/methyl alcohol 99/1 – 90/10) to afford a white solid (64.4 % yield; 1H NMR (300 MHz, DMSO-d6): δ = 12.93 (s, 1 H), 8.48 (s, 1 H), 7.92 (d, J = 8.5 Hz, 1 H), 7.77-7.73 (m, 2 H), 7.02 (d, J = 8.3 Hz, 2 H), 6.53 (d, J = 8.4 Hz, 2 H), 6.21 (d, J = 6.4 Hz, 1 H), 5.19 (s, 2 H), 5.07 (s, 2 H), 4.32 (s, 2 H) ppm; 13C NMR (75.5 MHz, DMSO-d6): δ = 167.8, 162.5, 161.4, 152.4, 151.8, 148.8, 132.6, 129.2, 124.3, 123.7, 123.5, 122.1, 121.6, 120.5, 114.3, 110.7, 47.3, 36.4 ppm; HPLC: 8.7 min, start at 3 % B; MS calcd. m/z 491.07, found m/z 489.9 [M-H]-).

*X = Signals were detected via COSY spectra
*X_2 = Signals were detected via HSQC and/or HMBC spectra

3-(((Benzyloxy)carbonyl)amino)propanoic acid (L2a)
Synthesis was done according to the described procedure using **β-Alanine** (1.00 equiv, 22.45 mmol, 2.00 g). The product was afforded as a white solid (65.6 % yield; ^{1}H NMR (300 MHz, DMSO-d6): δ = 12.20 (s, 1 H), 7.39-7.31 (m, 5 H), 7.28 (t, *J* = 5.5 Hz, 1 H), 5.00 (s, 2 H), 3.20 (q, ^{3}J = 5.9 Hz, 2 H), 2.39 (t, *J* = 7.0 Hz, 2 H) ppm; ^{13}C NMR (75.5 MHz, DMSO-d6): δ = 173.2, 156.5, 137.6, 128.8, 128.2, 65.7, 37.0, 34.6 ppm; HPLC: 6.1 min, start at 3 % B; MS calcd. *m/z* 223.08, found *m/z* 223.7 $[M+H]^+$).

4-(((Benzyloxy)carbonyl)amino)butanoic acid (L2b)
Synthesis was done according to the described procedure using **4-Aminobutanoic acid** (1.00 equiv. 21.08 mmol, 2.17 g). The product was afforded as a white solid (60.0 % yield; ^{1}H NMR (300 MHz, DMSO-d6): δ = 12.03 (s, 1 H), 7.39-7.29 (m, 5 H), 7.27 (t, J = 5.5 Hz, 1 H,), 5.00 (s, 2 H), 3.01 (q, J = 6.6 Hz, 2 H), 2.21 (t, J = 7.4 Hz, 2 H), 1.67-1.58 (m, 2 H) ppm; 13C NMR (75.5 MHz, DMSO-d6): δ = 174.6, 156.6, 137.8, 128.9, 128.2, 65.6, 40.2, 31.4, 25.3 ppm; HPLC: 6.7 min, start at 3 % B; MS calcd. *m/z* 237.10, found *m/z* 273.9 $[M+H]^+$).

5-(((Benzyloxy)carbonyl)amino)pentanoic acid (L2c)
Synthesis was done according to the described procedure using **5-Aminopentanoic acid** (1.00 equiv. 19.90 mmol, 3.06 g). The product was afforded as a white solid (56.0 % yield; 1H NMR (300 MHz, DMSO-d6): δ = 11.99 (s, 1 H), 7.39-7.29 (m, 5 H), 7.25 (t, J = 5.6 Hz, 1 H), 5.00 (s, 2 H), 2.98 (q, J = 6.4 Hz, 2 H), 2.20 (t, J = 7.2 Hz, 2 H), 1.52-1.44 (m, 2 H), 1.44-1.36 (m, 2 H) ppm; 13C NMR (75.5 MHz, DMSO-d6): δ = 174.8, 156.6, 137.8, 128.8, 128.2, 65.6, 40.4, 33.7, 29.4, 22.2 ppm; HPLC: 7.1 min, start at 3 % B; MS calcd. *m/z* 251.12, found *m/z* 251.9 $[M+H]^+$).

6-(((Benzyloxy)carbonyl)amino)hexanoic acid (L2d)
Synthesis was done according to the described procedure using **6-Aminohexanoic acid** (1.00 equiv. 18.86 mmol, 2.47 g). The product was afforded as a white solid (94.0 % yield; 1H NMR (300 MHz, DMSO-d6): δ = 11.94 (s, 1 H), 7.39-7.29 (m, 5 H), 7.22 (t, J = 5.6 Hz, 1 H), 5.00 (s, 2 H), 2.97 (q, J = 6.2 Hz, 2 H), 2.18 (t, J = 7.3 Hz, 2 H), 1.52-1.44 (m, 2 H), 1.43-1.35 (m, 2 H), 1.29-1.21 (m, 2) ppm; 13C NMR (75.5 MHz, DMSO-d6): δ = 174.9, 156.5, 137.8, 128.8, 128.2, 65.5, 40.6, 34.0, 29.6, 26.3, 24.7 ppm; HPLC: 7.5 min, start at 3 % B; MS calcd. m/z 265.13, found m/z 265.9 $[M+H]^+$).

Tert-butyl 3-(((benzyloxy)carbonyl)amino)propanoate (L3a)
Synthesis was done according to the described procedure using intermediate **L2a** (1.00 equiv. 14.69 mmol, 3.28 g). The product was afforded as a yellow oil (75.7 % yield; 1H NMR (300 MHz, DMSO-d6): δ = 7.40-7.26 (m, 5 H), 7.39-7.30 (t, *J* = 5.4 Hz, 1 H), 5.00 (s, 2 H), 3.20 (q, ^{3}J = 6.0 Hz, 2 H), 2.35 (t, ^{3}J = 6.9 Hz, 2 H), 1.38 (s, 9 H) ppm; 13C NMR (75.5 MHz, DMSO-d6): δ = 170.9, 156.5, 137.6, 128.8, 128.2, 80.3, 65.7, 37.1, 35.8, 28.2 ppm; HPLC: 7.6 min, start at 3 % B; MS calcd. m/z 279.15, found m/z 279.7 $[M+H]^+$).

Tert-butyl 4-(((benzyloxy)carbonyl)amino)butanoate (L3b)
Synthesis was done according to the described procedure using intermediate **L2b** (1.00 equiv. 10.96 mmol, 2.60 g). The product was afforded as a yellow oil (75.7 % yield; 1H NMR (300 MHz, DMSO-d6): δ = 7.38-7.30 (m, 5 H), 7.26 (t, J = 5.4 Hz, 1 H), 5.00 (s, 2 H), 2.99 (q, J = 6.5 Hz, 2 H), 2.19 (t, J = 7.5 Hz, 2 H), 1.66-1.57 (m, 2 H), 1.39 (s, 9 H) ppm; 13C NMR (75.5 MHz, DMSO-d6): δ = 172.4, 156.6, 137.7, 128.8, 128.2, 80.0, 65.6, 40.2, 31.4, 28.4, 25.3 ppm; HPLC: 9.2 min, start at 3 % B; MS calcd. m/z 293.16, found m/z 294.0 $[M+H]^+$).

Tert-butyl 5-(((benzyloxy)carbonyl)amino)pentanoate (L3c)
Synthesis was done according to the described procedure using intermediate **L2c** (1.00 equiv. 13.49 mmol, 3.39 g). The product was afforded as an orange oil (83.0 % yield; 1H NMR (300 MHz, DMSO-d6): δ = 7.39-7.29 (m, 5 H), 7.25 (t, J = 5.5 Hz, 1 H), 5.00 (s, 2 H), 2.98 (q, J = 6.0 Hz, 2 H), 2.18 (t, J = 6.9 Hz, 2 H), 1.51-1.41 (m, 4 H), 1.39 (s, 9 H) ppm; 13C NMR (75.5 MHz, DMSO-d6): δ = 172.8, 156.5, 137.7, 128.9, 128.2, 80.0, 65.2, 40.5, 33.8, 29.6, 28.1, 22.3 ppm; HPLC: 9.5 min, start at 3 % B; MS calcd. m/z 307.18, found m/z 308.0 $[M+H]^+$).

Tert-butyl 6-(((benzyloxy)carbonyl)amino)hexanoate (L3d)
Synthesis was done according to the described procedure using intermediate **L2d** (1.00 equiv. 9.34 mmol, 2.47 g). The product was afforded as an orange oil (87.00 % yield; 1H NMR (300 MHz, DMSO-d6): δ = 7.39-7.30 (m, 5 H), 7.22 (t, J = 5.4 Hz, 1 H), 5.00 (s, 2 H), 2.97 (q, J = 6.3 Hz, 2 H), 2.16 (t, J = 7.3 Hz, 2 H), 1.49-1.44 (m, 2 H), 1.43-1.34 (m, 11 H), 1.27-1.22 (m, 2) ppm; 13C NMR (75.5 MHz, DMSO-d6): δ = 172.7, 156.5, 137.7, 128.8, 128.1, 65.5, 40.5, 35.2, 28.3, 28.2, 26.1, 24.8 ppm; HPLC: 9.9 min, start at 3 % B; MS calcd. m/z 321.19, found m/z 322.0 $[M+H]^+$).

Tert-butyl 3-aminopropanoate (L4a)
Synthesis was done according to the described procedure using intermediate **L3a** (1.00 equiv. 11.29 mmol, 3.16 g). The product was afforded as a colourless oil (76.3 % yield; 1H NMR (300 MHz, DMSO-d6): δ = 2.86 (t, J = 6.5 Hz, 2 H), 2.42 (t, J = 6.5 Hz, 2 H), 1.45 (s, 9 H) ppm; 13C NMR (75.5 MHz, DMSO-d6): δ = 172.0, 80.5, 37.1, 36.8, 27.0 ppm; HPLC: 3.9 min, start at 3 % B; MS calcd. m/z 145.11, found m/z 145.8 $[M+H]^+$).

*X = Signals were detected via COSY spectra
*X_2 = Signals were detected via HSQC and/or HMBC spectra

Tert-butyl 4-aminobutanoate (L4b)
Synthesis was done according to the described procedure using intermediate **L3b** (1.00 equiv. 8.27 mmol, 2.45 g). The product was afforded as a colourless oil (81.2 % yield; 1H NMR (300 MHz, DMSO-d6): δ = 2.62 (t, J = 7.4 Hz, 2 H), 2.20 (t, J = 7.4 Hz, 2 H), 1.70-1.62 (m, 2 H), 1.45 (s, 9 H) ppm; 13C NMR (75.5 MHz, DMSO-d6): δ = 173.0, 80.3, 40.1, 32.3, 26.9, 26.5 ppm; HPLC: 4.2 min, start at 3 % B; MS calcd. m/z 159.13, found m/z 159.9 $[M+H]^+$).

Tert-butyl 5-aminopentanoate (L4c)
Synthesis was done according to the described procedure using intermediate **L3c** (1.00 equiv. 8.08 mmol, 2.48 g). The product was afforded as a yellow oil (80.0 % yield; 1H NMR (300 MHz, DMSO-d6): δ = 2.69 (t, J = 7.2 Hz, 2 H), 2.25 (t, J = 7.0 Hz, 2 H), 1.63-1.59 (m, 2 H), 1.55-1.48 (m, 2 H) 1.45 (s, 9 H) ppm; 13C NMR (75.5 MHz, DMSO-d6): δ = 173.3, 80.0, 40.2, 34.5, 26.9, 27.0 ppm; HPLC: 4.9 min, start at 3 % B; MS calcd. m/z 173.14, found m/z 173.9 $[M+H]^+$).

Tert-butyl 6-aminohexanoate (L4d)
Synthesis was done according to the described procedure using intermediate **L3d** (1.00 equiv. 8.12 mmol, 2.61 g). The product was afforded as a yellow oil (80.0 % yield; 1H NMR (300 MHz, DMSO-d6): δ = 2.72 (t, J = 7.2 Hz, 2 H), 2.24 (t, J = 7.0 Hz, 2 H), 1.63-1.57 (m, 4 H) 1.44 (s, 9 H), 1.40-1.36 (m, 2 H) ppm; 13C NMR (75.5 MHz, DMSO-d6): δ = 173.5, 80.0, 40.3, 34.9, 30.4, 27.1, 26.0, 24.6 ppm; HPLC: 5.1 min, start at 3 % B; MS calcd. m/z 187.16, found m/z 187.9 $[M+H]^+$).

Tert-butyl 3-((2-(2,6-dioxopiperidin-3-yl)-1,3-dioxoisoindolin-4-yl)amino)propanoate (L6a)
Synthesis was done according to the described procedure using intermediate **L4a** (1.30 equiv. 5.20 mmol, 755 mg). The product was afforded as a yellow solid (12.3 % yield; 1H NMR (300 MHz, DMSO-d6): δ = 11.09 (s, 1 H), 7.60 (dd, *J* = 8.9 Hz, 7.2 Hz, 1 H), 7.16 (d, *J* = 8.6 Hz, 1 H), 7.06 (d, *J* = 7.0 Hz, 1 H), 6.64 (t, *J* = 6.2 Hz, 1 H), 5.06 (dd, *J* = 12.9 Hz, 5.4 Hz, 1 H), 3.54 (q, *J* = 6.3 Hz, 2 H), 2.94-2.84 (m, 1 H), 2.63-2.56 (m, 2 H), 2.54 (t, *J* = 6.4 Hz, 2 H), 2.06-1.99 (m, 1 H), 1.40 (s, 9 H) ppm; 13C NMR (75.5 MHz, DMSO-d6): δ = 173.3, 171.2, 170.5, 169.3, 167.7, 146.5, 136.8, 132.7, 117.7, 111.2, 109.9, 80.7, 49.0, 38.5, 35.2, 31.4, 28.2, 22.6 ppm; HPLC: 7.4 min, start at 3 % B; MS calcd. m/z 401.16, found m/z 399.8 $[M-H]^-$).

Tert-butyl 4-((2-(2,6-dioxopiperidin-3-yl)-1,3-dioxoisoindolin-4-yl)amino)butanoate (L6b)
Synthesis was done according to the described procedure using intermediate **L4b** (1.30 equiv. 1.68 mmol, 268 mg). The product was afforded as a yellow solid (27.3 % yield; 1H NMR (300 MHz, DMSO-d6): δ = 11.09 (s, 1 H), 7.58 (dd, J = 8.3 Hz, 7.21 Hz, 1 H,), 7.11 (d, J = 8.7 Hz, 1 H), 7.02 (d, J = 7.0 Hz, 1 H), 6.64 (t, J = 6.2 Hz, 1 H), 5.05 (dd, J = 12.9 Hz, 5.4 Hz, 1 H), 3.31 (*X), 2.94-2.83 (m, 1 H), 2.64-2.52 (m, 2 H), 2.28 (t, J = 7.3 Hz, 2 H), 2.10-2.00 (m, 1 H), 1.83-1.73 (m, 2 H), 1.39 (s, 9 H) ppm; 13C NMR (75.5 MHz, DMSO-d6): δ = 173.3, 172.5, 170.6, 169.3, 167.8, 146.8, 136.8, 132.9, 117.6, 111.0, 109.8, 80.4, 49.0, 41.6, 32.5, 31.5, 28.3, 24.7, 22.5 ppm; HPLC: 8.9 min, start at 3 % B; MS calcd. m/z 415.17, found m/z 414.0 $[M-H]^-$).

Tert-butyl 5-((2-(2,6-dioxopiperidin-3-yl)-1,3-dioxoisoindolin-4-yl)amino)pentanoate (L6c)
Synthesis was done according to the described procedure using intermediate **L4c** (1.30 equiv. 8.39 mmol, 1.45 g). The product was afforded as a green solid (18.8 % yield; 1H NMR (300 MHz, DMSO-d6): δ = 11.09 (s, 1 H), 7.57 (dd, J = 8.7 Hz, 7.2 Hz, 1 H), 7.09 (d, J = 8.5 Hz, 1 H), 7.02 (d, J = 6.9 Hz, 1 H), 6.57 (t, J = 5.9 Hz, 1 H), 5.05 (dd, J = 13.2 Hz, 5.3 Hz, 1 H), 3.32-3.27 (m, 2 H), 2.93-2.83 (m, 1 H), 2.63-2.52 (m, 2 H), 2.26-2.22 (m, 2 H), 2.05-2.00 (m, 1 H), 1.59-1.54 (m, 4 H), 1.37 (s, 9 H) ppm; 13C NMR (75.5 MHz, DMSO-d6): δ = 173.3, 172.6, 170.6, 169.4, 167,8, 146.9, 136.7, 132.7, 117.7, 110.9, 109.6, 80.1, 49.0, 41.9, 34.9, 31.5, 28.5, 28.2, 22.6, 22.4 ppm; HPLC: 9.3 min, start at 3 % B; MS calcd. m/z 429.19, found m/z 428.0 $[M-H]^-$).

Tert-butyl 6-((2-(2,6-dioxopiperidin-3-yl)-1,3-dioxoisoindolin-4-yl)amino)hexanoate (L6d)
Synthesis was done according to the described procedure using intermediate **L4d** (1.30 equiv. 2.93 mmol, 549 mg). The product was afforded as a green solid (23.0 % yield; 1H NMR (300 MHz, DMSO-d6): δ = 11.09 (s, 1 H), 7.57 (dd, J = 8.9 Hz, 7.3 Hz, 1 H), 7.09 (d, J = 8.7 Hz, 1 H), 7.01 (d, J = 7.0 Hz, 1 H), 6.53 (t, J = 5.9 Hz, 1 H), 5.05 (dd, J = 13.0 Hz, 5.3 Hz, 1 H), 3.28 (q, J = 6.5 Hz, 2 H), 2.93-2.83 (m, 1 H), 2.63-2.52 (m, 2 H), 2.19 (t, J = 7.3 Hz, 2 H), 2.06-2.00 (m, 1 H), 1.61-1.48 (m, 4 H), 1.37 (s, 9 H), 1.35-1.31 (m, 2 H) ppm; 13C NMR (75.5 MHz, DMSO-d6): δ = 173.3, 172.7, 170.5, 169.4, 167,8, 146.9, 136.7, 132.7, 117.7, 110.8, 109.5, 79.9, 49.0, 42.1, 35.1, 31.4, 28.8, 28.2, 26.2, 24.9 ppm; HPLC: 9.7 min, start at 3 % B; MS calcd. m/z 443.21, found m/z 442.1 $[M-H]^-$).

Tert-butyl 3-((2-(2,6-dioxopiperidin-3-yl)-1,3-dioxoisoindolin-4-yl)amino)propanoate (L8a)
Synthesis was done according to the described procedure using intermediate **L4a** (1.30 equiv. 2.45 mmol, 355 mg). The product was afforded as a yellow solid (8.2 % yield; 1H NMR (300 MHz, DMSO-d6): δ = 11.05 (s, 1 H), 7.57 (d, *J* = 8.3 Hz, 1 H), 7.14 (t, *J* = 5.7 Hz, 1 H), 6.97 (d,^3J = 1.9 Hz, 1 H), 6.87 (dd, *J* = 8.4 Hz, 2.1 Hz, 1 H), 5.03 (dd, *J* = 12.9 Hz, 5.4 Hz, 1 H), 3.40 (q, *J* = 6.3 Hz, 2 H), 2.93-2.82 (m, 1 H), 2.61-2.52 (m, 2 H), 2.52 (*X), 2.03-1.96 (m, 1 H), 1.40 (s, 9 H) ppm; 13C NMR (75.5 MHz, DMSO-d6): δ = 173.3, 171.0, 170.6, 168.1, 167.6, 154.6, 134.7, 125.6, 116.8, 116.0, 105.8, 80.6, 49.1, 38.9, 34.9, 31.5, 28.2, 22.7 ppm; HPLC: 6.3 min, start at 3 % B; MS calcd. m/z 401.16, found m/z 400,2 $[M-H]^-$).

Tert-butyl 4-((2-(2,6-dioxopiperidin-3-yl)-1,3-dioxoisoindolin-5-yl)amino)butanoate (L8b)
Synthesis was done according to the described procedure using intermediate **L4b** (1.30 equiv. 3.76 mmol, 598 mg). The product was afforded as a green solid (10.8 % yield; 1H NMR (300 MHz, DMSO-d6): δ = 11.05 (s, 1 H), 7.56 (d, J = 8.4 Hz, 1 H), 7.14 (t,

*X = Signals were detected via COSY spectra
*X_2 = Signals were detected via HSQC and/or HMBC spectra

J = 5.3 Hz, 1 H), 6.94 (d, J = 2.0 Hz, 1 H), 6.84 (dd, J = 8.4 Hz, 2.1 Hz, 1 H), 5.05 (dd, J = 12.8 Hz, 5.3 Hz, 1 H), 3.18 (q, J = 6.2 Hz, 2 H), 2.92-2.82 (m, 1 H), 2.61-2.52 (m, 2 H), 2.32 (t, J = 7.4 Hz, 2 H), 2.03-1.95 (m, 1 H), 1.81-1.73 (m, 2 H), 1.40 (s, 9 H) ppm; 13C NMR (75.5 MHz, DMSO-d6): δ = 173.3, 172.4, 170.6, 168.1, 167.6, 154.8, 134.5, 125.5, 116.5, 116.0, 105.8, 80.2, 49.1, 42.1, 32.6, 31.5, 28.2, 24.3, 22.7 ppm; HPLC: 8.5 min, start at 3 % B; MS calcd. m/z 415.17, found m/z 414.1 $[M-H]^-$).

Tert-butyl 5-((2-(2,6-dioxopiperidin-3-yl)-1,3-dioxoisoindolin-5-yl)amino)pentanoate (L8c)
Synthesis was done according to the described procedure using intermediate **L4c** (1.30 equiv. 5.56 mmol, 964 mg). The product was afforded as a green solid (11.9 % yield; 1H NMR (300 MHz, DMSO-d6): δ = 11.05 (s, 1 H), 7.55 (d, J = 8.4 Hz, 1 H), 7.12 (t, J = 5.3 Hz, 1 H), 6.94 (d, J = 1.9 Hz, 1 H), 6.84 (dd, J = 8.4 Hz, 4J = 2.04 Hz, 1 H), 5.05 (dd, J = 13.1 Hz, 5.5 Hz, 1 H), 3.16 (q, J = 5.5 Hz, 2 H), 2.92-2.82 (m, 1 H), 2.61-2.52 (m, 2 H), 2.24 (t, J = 6.8 Hz, 2 H), 2.03-1.95 (m, 1 H), 1.61-1.55 (m, 4 H), 1.38 (s, 9 H) ppm; 13C NMR (75.5 MHz, DMSO-d6): δ = 173.3, 172.7, 170.6, 168.2, 167.6, 154.9, 134.7, 125.6, 116.3, 115.7, 105.7, 79.9, 49.1, 42.8, 35,2, 31.5 , 28.3, 28.2, 24.8, 22.7 ppm; HPLC: 8.8 min, start at 3 % B; MS calcd. m/z 429.19, found m/z 428.1 $[M-H]^-$).

Tert-butyl 6-((2-(2,6-dioxopiperidin-3-yl)-1,3-dioxoisoindolin-5-yl)amino)hexanoate (L8d)
Synthesis was done according to the described procedure using intermediate **L4d** (1.30 equiv. 2.40 mmol, 450 mg). The product was afforded as a green solid (9.8 % yield; 1H NMR (300 MHz, DMSO-d6): δ = 11.05 (s, 1 H), 7.55 (d, J = 8.3 Hz, 1 H), 7.09 (t, J = 5.2 Hz, 1 H), 6.94 (d, J = 1.9 Hz, 1 H), 6.84 (dd, J = 8.4 Hz, 2.0 Hz, 1 H), 5.05 (dd, J = 13.1 Hz, 5.34 Hz, 1 H), 3.15 (q, J = 6.8 Hz, 2 H), 2.93-2.82 (m, 1 H), 2.60-2.52 (m, 2 H), 2.19 (t, J = 7.3 Hz, 2 H), 2.03-1.95 (m, 1 H), 1.61-1.49 (m, 4 H), 1.38 (s, 9 H), 1.37-1.34 (m, 2 H) ppm; 13C NMR (75.5 MHz, DMSO-d6): δ = 173.3, 172.7, 170.6, 168.2, 167.6, 154.9, 134.7, 125.5, 116.3, 115.8, 105.6, 79.9, 49.1, 42.8, 35.2, 31.5, 28.4, 28.2, 26.6, 24.9, 22.7 ppm; HPLC: 9.2 min, start at 3 % B; MS calcd. m/z 443.21, found m/z 442.1 $[M-H]^-$).

Tert-butyl-3-(2-hydroxyethoxy)propanoate (L10a)
Synthesis was done according to the described procedure using **Ethylene glycol** (5.00 equiv. 60.0 mmol, 3.72 g). The product was afforded as a colourless oil (30.0 % yield; 1H NMR (300 MHz, DMSO-d6): δ = 4.55 (t, J = 5.4 Hz, 1 H, 3.59 (t, J = 6.3 Hz, 2 H), 3.49-3.38 (m, 4 H, H-1), 2.42 (t, J = 6.3 Hz, 2 H), 1.40 (s, 9 H) ppm; 13C NMR (75.5 MHz, DMSO-d6): δ = 170.9, 80.2, 72.6, 66.7, 60.6, 36.3, 28.3 ppm; HPLC: 6.3 min, start at 3 % B; MS calcd. m/z 190.12, found m/z 212.9 $[M+Na]^+$).

Tert-butyl-3-(2-(2-hydroxyethoxy)ethoxy)propanoate (L10b)
Synthesis was done according to the described procedure using **Diethylene glycol** (5.00 equiv. 47.5 mmol, 5.04 g). The product was afforded as a colourless oil (72.0 % yield; 1H NMR (300 MHz, DMSO-d6): δ = 4.56 (t, J = 5.6 Hz, 1 H), 3.59 (t, J = 6.3 Hz, 2 H), 3.48 (m, 4 H), 3.41 (m, 4 H, H-2), 2.42 (t, J = 6.3 Hz, 2 H), 1.40 (s, 9 H) ppm; 13C NMR (75.5 MHz, DMSO-d6): δ = 170.9, 80.1, 72.8, 70, 66.7, 60.7, 36.3, 28.23 ppm; HPLC: 6.3 min, start at 3 % B; MS calcd. m/z 190.12, found m/z 212.9 $[M+Na]^+$).

Tert-butyl-3-(2-(2-(2-hydroxyethoxy)ethoxy)ethoxy-)propanoate (L10c)
Synthesis was done according to the described procedure using **Triethylene glycol** (5.00 equiv. 20.8 mmol, 3.13 g). The product was afforded as a colourless oil (64.0 % yield; 1H NMR (300 MHz, DMSO-d6): δ = 4.56 (t, J = 5.6 Hz, 1 H), 3.59 (t, J = 6.3 Hz, 2 H), 3.49 (m, 10 H), 3.41 (m, 2 H), 2.42 (t, J = 6.3 Hz, 2 H), 1.40 (s, 9H) ppm; 13C NMR (75.5 MHz, DMSO-d6): δ = 170.9, 80.2, 72.8, 70.2, 66.7, 60.7, 36.3, 28.2 ppm; HPLC: 6.7 min, start at 3 % B; MS calcd. m/z 234.15, found m/z 257.0 $[M+Na]^+$).

Tert-butyl-3-(3-hydroxypropoxy)propanoate (L10d)
Synthesis was done according to the described procedure using **1,3-Propanediol** (5.00 equiv. 60.0 mmol, 4.57 g). The product was afforded as a colourless oil (70.0 % yield; 1H NMR (300 MHz, DMSO-d6): δ = 4.35 (t, J = 5.2 Hz, 1 H), 3.54 (t, J = 6.2 Hz, 2 H), 3.43 (m, 4 H), 2.40 (t, J = 6.2 Hz, 2 H), 1.62 (m, 2 H), 1.40 (s, 9 H) ppm; 13C NMR (75.5 MHz, DMSO-d6): δ = 171.0, 80.1, 67.8, 66.4, 58.3, 36.4, 33.1, 28.2 ppm; HPLC: 6.6 min, start at 3 % B; MS calcd. m/z 204.14, found m/z 226.9 $[M+Na]^+$).

Tert-butyl 3-(2-(tosyloxy)ethoxy)propanoate (L11a)
Synthesis was done according to the described procedure intermediate **L10a** (1.00 equiv. 1.16 mmol, 221 mg). The product was afforded as a colourless oil (57.7 % yield; 1H NMR (300 MHz, DMSO-d6): δ = = 7.80-7.76 (m, 2 H), 7.50-7.46 (m, 2 H), 4.11-4.08 (m, 2 H), 3.56-3.54 (m, 2 H), 3.51 (t, J = 6.2 Hz, 2 H), 2.42 (s, 3 H), 2.35 (t, J = 6.1 Hz, 2 H), 1.38 (s, 9 H) ppm; 13C NMR (75.5 MHz, DMSO-d6): δ = 170.7, 145.5, 145.4, 130.6, 128.1, 80.4, 70.3, 68.3, 66.6, 36.2, 28.2, 21.5 ppm; HPLC: 7.6 min, start at 3 % B; MS calcd. m/z 344.13, found m/z 366.8 $[M+Na]^+$).

Tert-butyl 3-(2-(2-(tosyloxy)ethoxy)ethoxy)propanoate (L11b)
Synthesis was done according to the described procedure using intermediate **L10b** (1.00 equiv. 1.03 mmol, 241 mg). The product was afforded as a colourless oil (57.7 % yield; 1H NMR (300 MHz, DMSO-d6): δ = 7.80-7.76 (m, 2 H), 7.50-7.46 (m, 2 H), 4.11-4.08 (m, 2 H), 3.57-3.56 (m, 2 H), 3.54 (t, J = 6.3 Hz, 2 H), 3.43-3.42 (m, 4 H), 2.42 (s, 3 H), 2.39 (t, J = 6.27 Hz, 2 H), 1.38 (s, 9 H) ppm; 13C NMR (75.5 MHz, DMSO-d6): δ = 170.8, 145.5, 145.4, 130.6, 128.1, 80.4, 70.5, 70.1, 68.3, 66.7, 36.3, 28.3, 21.5 ppm; HPLC: 9.6 min, start at 3 % B; MS calcd. m/z 388.16, found m/z 411.0 $[M+Na]^+$).

*X = Signals were detected via COSY spectra
*X_2 = Signals were detected via HSQC and/or HMBC spectra

Tert-butyl 3-(2-(2-(2-(tosyloxy)ethoxy)ethoxy)ethoxy)propanoate (L11c)

Synthesis was done according to the described procedure using intermediate **L10c** (1.00 equiv. 2.31 mmol, 644 mg). The product was afforded as a colourless oil (93.6 % yield; 1H NMR (300 MHz, DMSO-d6): δ = 7.80-7.76 (m, 2 H), 7.50-7.45 (m, 2 H), 4.12-4.08 (m, 2 H, *H*-8), 3.59-3.55 (m, 4 H), 3.46 (s, 4 H), 3.44 (s, 4 H), 2.42 (s, 3 H), 2.40 (t, J = 6.3 Hz, 2 H), 1.38 (s, 9 H) ppm; 13C NMR (75.5 MHz, DMSO-d6): δ = 170.9, 145.5, 145.4, 130.6, 128.1, 80.3, 70.5, 70.2, 70.1, 68.4, 66.7, 36.4, 28.3, 21.5 ppm; HPLC: 9.6 min, start at 3 % B; MS calcd. m/z 432.18, found m/z 455.1 $[M+Na]^+$).

Tert-butyl 3-(3-(tosyloxy)propoxy)propanoate (L11d)

Synthesis was done according to the described procedure using intermediate **L10d** (1.00 equiv. 1.12 mmol, 228 mg). The product was afforded as a colourless oil (75.8 % yield; 1H NMR (300 MHz, DMSO-d6): δ = 7.80-7.75 (m, 2 H), 7.50-7.46 (m, 2 H), 4.04 (t, J = 6.4 Hz, 2 H), 3.44 (t, J = 6.2 Hz, 2 H), 3.33 (t, J = 6.1 Hz, 2 H), 2.43 (s, 3 H), 2.33 (t, J = 6.3 Hz, 2 H), 1.81-1.73 (m, 2 H), 1.38 (s, 9 H) ppm; 13C NMR (75.5 MHz, DMSO-d6): δ = 170.8, 145.5, 145.4, 130.6, 128.0, 80.2, 68.6, 66.4, 66.1, 36.2, 29.0, 28.2, 21.6 ppm; HPLC: 10.0 min, start at 3 % B; MS calcd. m/z 358.15, found m/z 381.0 $[M+Na]^+$).

Tert-butyl 3-(2-((2-(2,6-dioxopiperidin-3-yl)-1,3-dioxoisoindolin-4-yl)oxy)ethoxy)propanoate (L13a)

Synthesis was done according to the described procedure using intermediate **L11a** (1.20 equiv. 1.08 mmol, 373 mg). The product was afforded as a colourless oil (44.2 % yield; 1H NMR (300 MHz, DMSO-d6): δ = 11.10 (s, 1 H), 7.81 (dd, J = 8.8 Hz, 7.4 Hz, 1 H), 7.52 (d, J = 8.4 Hz, 1 H), 7.46 (d, J = 7.1 Hz, 1 H), 5.08 (dd, J = 12.9 Hz, 5.4 Hz, 1 H), 4.35-4.31 (m, 2 H), 3.79-3.76 (m, 2 H), 3.71 (t, J = 6.2 Hz, 2 H), 2.93-2.83 (m, 1 H), 2.62-2.52 (m, 2 H), 2.43 (t, J = 6.2 Hz, 2 H), 2.06-1.99 (m, 1 H), 1.36 (s, 9 H) ppm; 13C NMR (75.5 MHz, DMSO-d6): δ = 173.2, 170.8, 170.4, 167,3, 165.7, 156.3, 137.5, 133.7, 120.5, 116.8, 115.9, 80.3, 69.3, 69.0, 67.1, 49.2, 36.4, 31.4, 28.2, 22.5 ppm; HPLC: 8.3 min, start at 3 % B; MS calcd. m/z 446.17, found m/z 445.1 $[M-H]^-$).

Tert-butyl 3-(2-(2-((2-(2,6-dioxopiperidin-3-yl)-1,3-dioxoisoindolin-4-yl)oxy)ethoxy)ethoxy)-propanoate (L13b)

Synthesis was done according to the described procedure using intermediate **L11b** (1.20 equiv. 0.98 mmol, 380 mg). The product was afforded as a colourless oil (48.5 % yield; 1H NMR (300 MHz, DMSO-d6): δ = 11.10 (s, 1 H), 7.81 (dd, J = 8.5 Hz, 7.3 Hz, 1 H), 7.53 (d, J = 8.45 Hz, 1 H), 7.46 (d, J = 7.2 Hz, 1 H), 5.08 (dd, J = 13.0 Hz, 5.44 Hz, 1 H), 4.35-4.31 (m, 2 H), 3.81-3.78 (m, 2 H), 3.64-3.61 (m, 2 H), 3.58 (t, J = 6.3 Hz), 3.52-3.49 (m, 2 H), 2.94-2.83 (m, 1 H), 2.62-2.52 (m, 2 H), 2.39 (t, J = 6.27 Hz, 2 H), 2.06-1.98 (m, 1 H), 1.37 (s, 9 H) ppm; 13C NMR (75.5 MHz, DMSO-d6): δ = 173.2, 170.9, 170.4, 167,3, 165.7, 156.3, 137.4, 133.7, 120.5, 116.8, 115.9, 80.2, 70.6, 70.2, 69.4, 69.2, 66.7, 49.2, 36.4, 31.5, 28.2, 22.5 ppm; HPLC: 8.1 min, start at 3 % B; MS calcd. m/z 490.20, found m/z 489.1 $[M-H]^-$).

Tert-butyl 3-(2-(2-(2-((2-(2,6-dioxopiperidin-3-yl)-1,3-dioxoisoindolin-4-yl)oxy)ethoxy)ethoxy)-ethoxy)propanoate (L13c)

Synthesis was done according to the described procedure using intermediate **L11c** (1.20 equiv. 1.12 mmol, 485 mg). The product was afforded as a colourless oil (80.0 % yield; 1H NMR (300 MHz, DMSO-d6): δ = 11.10 (s, 1 H), 7.81 (dd, J = 8.6 Hz, 7.3 Hz, 1 H), 7.53 (d, J = 8.4 Hz, 1 H), 7.46 (d, J = 7.2 Hz, 1 H), 5.08 (dd, J = 13.0 Hz, 5.4 Hz, 1 H), 4.36-4.32 (m, 2 H), 3.82-3.78 (m, 2 H), 3.65-3.62 (m, 2 H), 3.57 (t, J = 6.3 Hz, 2 H), 3.54-3.51 (m, 2 H), 3.49-3.46 (m, 4 H), 2.94-2.83 (m, 1 H), 2.63-2.52 (m, 2 H), 2.39 (t, J = 6.3 Hz, 2 H), 2.06-1.99 (m, 1 H), 1.38 (s, 9 H) ppm; 13C NMR (75.5 MHz, DMSO-d6): δ = 173.2, 170.9, 170.4, 167,3, 165.7, 156.3, 137.5, 133.7, 120.5, 116.8, 115.9, 80.2, 70.6, 70.3, 70.2, 70.1, 69.3, 69.2, 66.7, 49.2, 36.3, 31.4, 28.2, 22.5 ppm; HPLC: 8.1 min, start at 3 % B; MS calcd. m/z 534.22, found m/z 533.2 $[M-H]^-$).

Tert-butyl 3-(3-((2-(2,6-dioxopiperidin-3-yl)-1,3-dioxoisoindolin-4-yl)oxy)propoxy)propanoate (L13d)

Synthesis was done according to the described procedure using intermediate **L11d** (1.20 equiv. 0.80 mmol, 288 mg). The product was afforded as a colourless oil (47.1 % yield; 1H NMR (300 MHz, DMSO-d6): δ = 11.10 (s, 1 H), 7.81 (dd, J = 9.5 Hz, 7.3 Hz, 1 H), 7.50 (d, J = 8.4 Hz, 1 H), 7.45 (d, J = 7.2 Hz, 1 H), 5.08 (dd, J = 13.0 Hz, 5.5 Hz, 1 H), 4.24 (t, J = 6.7 Hz, 2 H), 3.57 (t, J = 6.0 Hz, 4 H), 2.93-2.83 (m, 1 H), 2.62-2.52 (m, 2 H), 2.41 (t, J = 6.1 Hz, 2 H), 2.05-2.00 (m, 1 H), 2.00-1.94 (m, 2 H), 1.35 (s, 9 H) ppm; 13C NMR (75.5 MHz, DMSO-d6): δ = 173.3, 170.9, 170.4, 167,3, 165.8, 156.4, 137.5, 133.7, 120.2, 116.8, 115.7, 80.3, 66.7, 66.4, 66.2, 49.2, 36.2, 31.5, 29.2, 28.2, 22.5 ppm; HPLC: 8.4 min, start at 3 % B; MS calcd. m/z 460.18, found m/z 459.1 $[M-H]^-$).

Tert-butyl 3-(2-((2-(2,6-dioxopiperidin-3-yl)-1,3-dioxoisoindolin-5-yl)oxy)ethoxy)propanoate (L15a)

Synthesis was done according to the described procedure using intermediate **L11a** (1.20 equiv. 1.45 mmol, 500 mg). The product was afforded as a colourless oil (55.7 % yield; 1H NMR (300 MHz, DMSO-d6): δ = 11.10 (s, 1 H), 7.83 (d, *J* = 8.3 Hz, 1 H), 7.43 (d, *J* = 2.2 Hz, 1 H), 7.34 (dd, *J* = 8.3 Hz, 2.3 Hz, 1 H), 5.11 (dd, *J* = 12.9 Hz, 5.4 Hz, 1 H), 4.31-4.27 (m, 2 H, *H*-4), 3.77-3.74 (m, 2 H, *H*-3), 3.67 (t, *J* = 6.2 Hz, 2 H), 2.94-2.84 (m, 1 H), 2.64-2.52 (m, 2 H), 2.44 (t, *J* = 6.2 Hz, 2 H), 2.09-2.01 (m, 1 H), 1.38 (s, 9 H) ppm; 13C NMR (75.5 MHz, DMSO-d6): δ = 173.2, 170.8, 170.4, 167.3, 164.4, 134.4, 125.7, 123.5, 121.3, 109.3, 80.2, 68.9, 68.8, 66.8, 49.4, 36.3, 31.4, 28.2, 22.5 ppm; HPLC: 6.4 min, start at 3 % B; MS calcd. m/z 446.17, found m/z 445.2 $[M-H]^-$).

Tert-butyl 3-(2-(2-((2-(2,6-dioxopiperidin-3-yl)-1,3-dioxoisoindolin-5-yl)oxy)ethoxy)ethoxy)propanoate (L15b)

Synthesis was done according to the described procedure using intermediate **L11b** (1.20 equiv. 0.90 mmol, 349 mg). The product was afforded as a colourless oil (66.5 % yield; 1H NMR (300 MHz, DMSO-d6): δ = 11.10 (s, 1 H), 7.83 (d, *J* = 8.3 Hz, 1 H), 7.45 (d, *J* = 2.2 Hz, 1 H), 7.36 (dd, *J* = 8.3 Hz, 2.3 Hz, 1 H), 5.12 (dd, *J* = 12.9 Hz, 5.4 Hz, 1 H), 4.32-4.28 (m, 2 H), 3.80-3.75 (m, 2 H), 3.61-3.56 (m, 4 H), 3.54-3.50 (m, 2 H), 2.94-2.83 (m, 1 H), 2.63-2.51 (m, 2 H), 2.41 (t, ^3J = 6.2 Hz, 2 H), 2.08-2.00 (m, 1 H), 1.38

*X = Signals were detected via COSY spectra

*X_2 = Signals were detected via HSQC and/or HMBC spectra

(s, 9 H); 13C NMR (75.5 MHz, DMSO-d6): δ = 173.2, 170.9, 170.4, 167.3, 164.4, 134.4, 125.7, 123.5, 121.3, 109.4, 80.2, 70.3, 70.2, 69.1, 68.9, 66.7, 49.4, 36.3, 31.4, 28.2, 22.5 ppm; HPLC: 6.4 min, start at 3 % B; MS calcd. m/z 490.20, found m/z 489.2 $[M-H]^-$).

Tert-butyl 3-(2-(2-(2-((2-(2,6-dioxopiperidin-3-yl)-1,3-dioxoisoindolin-5-yl)oxy)ethoxy)ethoxy)ethoxy)propanoate (L15c)
Synthesis was done according to the described procedure using intermediate **L11c** (1.20 equiv. 1.16 mmol, 500 mg). The product was afforded as a colourless oil (78.5 % yield; 1H NMR (300 MHz, DMSO-d6): δ = 11.11 (s, 1 H), 7.84 (d, *J* = 8.3 Hz, 1 H), 7.46 (d, *J* = 2.2 Hz, 1 H), 7.37 (dd, *J* = 8.3 Hz, 2.3 Hz, 1 H), 5.12 (dd, *J* = 12.9 Hz, 5.4 Hz, 1 H), 4.34-4.29 (m, 2 H), 3.81-3.77 (m, 2 H), 3.61-3.56 (m, 4 H), 3.56-3.51 (m, 2 H), 3.51-3.46 (m, 4 H), 2.95-2.84 (m, 1 H), 2.64-2.52 (m, 2 H), 2.41 (t, *J* = 6.2 Hz, 2 H), 2.08-2.01 (m, 1 H), 1.39 (s, 9 H) ppm; 13C NMR (75.5 MHz, DMSO-d6): δ = 173.2, 170.9, 170.4, 167.3, 164.4, 134.4, 125.7, 123.5, 121.3, 109.4, 80.2, 70.3, 70.2, 70.1, 69.1, 68.9, 66.7, 49.4, 36.3, 31.4, 28.2, 22.5 ppm; HPLC: 6.2 min, start at 3 % B; MS calcd. m/z 534.22, found m/z 533.2 $[M-H]^-$).

Tert-butyl 3-(3-((2-(2,6-dioxopiperidin-3-yl)-1,3-dioxoisoindolin-5-yl)oxy)propoxy)propanoate (L15d)
Synthesis was done according to the described procedure using intermediate **L11d** (1.20 equiv. 1.39 mmol, 499 mg). The product was afforded as a colourless oil (76.4 % yield; 1H NMR (300 MHz, DMSO-d6): δ = 11.11 (s, 1 H), 7.84 (d, *J* = 8.3 Hz, 1 H), 7.41 (d, *J* = 2.2 Hz, 1 H), 7.35 (dd, *J* = 8.3 Hz, 2.3 Hz, 1 H), 5.12 (dd, *J* = 12.9 Hz, 5.4 Hz, 1 H), 4.22 (t, *J* = 6.3 Hz, 2 H), 3.59 (t, *J* = 6.1 Hz, 2 H), 3.54 (t, *J* = 6.2 Hz, 2 H), 2.95-2.84 (m, 1 H), 2.64-2.52 (m, 2 H), 2.43 (t, *J* = 6.1 Hz, 2 H), 2.08-2.02 (m, 1 H), 2.02-1.95 (m, 2 H), 1.37 (s, 9 H) ppm; 13C NMR (75.5 MHz, DMSO-d6): δ = 173.2, 170.9, 170.4, 167.3, 164.5, 134.4, 125.8, 123.4, 121.2, 109.3, 80.2, 66.8, 66.4, 66.3, 49.4, 36.3, 31.4, 29.2, 28.2, 22.5 ppm; HPLC: 6.6 min, start at 3 % B; MS calcd. m/z 460.18, found m/z 459.2 $[M-H]^-$).

3-((2-(2,6-Dioxopiperidin-3-yl)-1,3-dioxoisoindolin-4-yl)amino)-N-(4-((6-oxo-2-((2-oxo-2-((6-(trifluoromethyl)benzo[d]thiazol-2-yl)amino)ethyl)thio)pyrimidin-1(6H)-yl)methyl)phenyl)propanamide (P1a)
Synthesis was done according to the described procedure using intermediate **L6a** (1.20 equiv. 0.28 mmol, 98 mg). The product was afforded as a yellow solid (6.4 % yield; 1H NMR (300 MHz, DMSO-d6): δ = 12.91 (s, 1 H), 11.08 (s, 1 H), 10.09 (s, 1 H), 8.48 (s, 1 H), 7.92 (d, J = 8.4 Hz, 1 H), 7.79 (d, J = 6.5 Hz, 1 H), 7.74 (dd, J = 8.4 Hz, 1.3 Hz, 1 H), 7.63-7.55 (m, 3 H), 7.23 (d, J = 8.4 Hz, 2 H), 7.19 (d, J = 8.7 Hz, 1 H), 7.04 (d, J = 7.0 Hz, 1 H), 6.75 (t, J = 5.9 Hz, 1 H), 6.25 (d, J = 6.46 Hz, 1 H), 5.21 (s, 2 H), 5.04 (dd, J = 13.0 Hz, 5.2 Hz, 1 H), 4.31 (s, 2 H), 3.63 (q, J = 13.0 Hz, 2 H), 2.92-2.81 (m, 1 H), 2.66 (t, J = 6.2 Hz, 2 H), 2.61-2.52 (m, 2 H), 2.05-1.97 (m, 1 H) ppm; 13C NMR (75.5 MHz, DMSO-d6): δ = 173.3, 170.6, 170.1, 169.3, 167.8, 162.6, 161.6, 161.4, 152.5, 151.8, 146.6, 139.0, 136.8, 132.7, 132.5, 130.0, 128.1, 124.3 (d, *J* = 31.9 Hz), 123.7, 123.5, 121.5, 120.4 (m), 119.8, 117.7, 111.1, 110.7, 109.9, 49.0, 47.1, 38.8, 36.4, 31.5, 22.6 ppm; HPLC: 16.44 min, start at 0.1 % B; MS calcd. m/z 818.1556, found m/z 819.1626 $[M+H]^+$).

4-((2-(2,6-Dioxopiperidin-3-yl)-1,3-dioxoisoindolin-4-yl)amino)-N-(4-((6-oxo-2-((2-oxo-2-((6-(trifluoromethyl)benzo[d]thiazol-2-yl)amino)ethyl)thio)pyrimidin-1(6H)-yl)methyl)phenyl)butanamide (P1b)
Synthesis was done according to the described procedure using intermediate **L6b** (1.20 equiv. 0.11 mmol, 39 mg). The product was afforded as a yellow solid (22.2 % yield; 1H NMR (300 MHz, DMSO-d6): δ = 12.93 (s, 1 H), 11.09 (s, 1 H), 9.99 (s, 1 H), 8.46 (s, 1 H), 7.90 (d, J = 8.5 Hz, 1 H), 7.79 (d, J = 6.4 Hz, 1 H), 7.73 (dd, J = 8.4 Hz, 1.3 Hz, 1 H), 7.61-7.55 (m, 3 H), 7.22 (d, J = 8.2 Hz, 2 H), 7.14 (d, J = 8.6 Hz, 1 H), 7.02 (d, J = 6.9 Hz, 1 H), 6.75 (t, J = 6.0 Hz, 1 H, 6.25 (d, J = 6.4 Hz, 1 H), 5.21 (s, 2 H), 5.04 (dd, J = 12.6 Hz, 5.20 Hz, 1 H), 4.30 (s, 2 H), 3.40-3.35 (m, 2 H), 2.94-2.84 (m, 1 H), 2.63-2.53 (m, 2 H), 2.41 (t, J = 7.4 Hz, 2 H), 2.06-1.98 (m, 1 H), 1.93-1.84 (m, 2 H) ppm; 13C NMR (75.5 MHz, DMSO-d6): δ = 173.3, 171.2, 170.6, 169.3, 167.7, 162.7, 161.4, 152.5, 151.8 (*X_2), 146.8, 139.2, 136.7, 132.7, 132.5, 129.8, 128.1, 124.5 (*X_2), 123.7 (*X_2), 123.5 (*X_2), 121.4 (*X_2), 120.4 (*X_2), 119.7, 117.7, 110.9, 110.6, 109.6, 49.0, 47.1 (*X_2), 41.9, 36.6 (*X_2), 33.9, 31.4, 24.9, 22.6 ppm; HPLC: 16.97 min, start at 0.1 % B; MS calcd. m/z 832.1709, found m/z 833.1780 $[M+H]^+$).

5-((2-(2,6-Dioxopiperidin-3-yl)-1,3-dioxoisoindolin-4-yl)amino)-N-(4-((6-oxo-2-((2-oxo-2-((6-(trifluoromethyl)benzo[d]thiazol-2-yl)amino)ethyl)thio)pyrimidin-1(6H)-yl)methyl)phenyl)pentanamide (P1c)
Synthesis was done according to the described procedure using intermediate **L6c** (1.00 equiv. 0.11 mmol, 39 mg). The product was afforded as a yellow solid (24.2 % yield; 1H NMR (300 MHz, DMSO-d6): δ = 12.91 (s, 1 H), 11.08 (s, 1 H), 9.95 (s, 1 H), 8.48 (s, 1 H), 7.91 (d, J = 8.5 Hz, 1 H), 7.79 (d, J = 6.5 Hz, 1 H), 7.74 (dd, J = 8.6 Hz, 1.5 Hz, 1 H), 7.59-7.53 (m, 3 H), 7.22 (d, J = 8.6 Hz, 2 H), 7.10 (d, J = 8.6 Hz, 1 H), 7.01 (d, J = 7.0 Hz, 1 H), 6.60 (t, J = 6.0 Hz, 1 H, 6.25 (d, J = 6.5 Hz, 1 H), 5.21 (s, 2 H), 5.04 (dd, J = 12.9 Hz, 5.4 Hz, 1 H), 4.31 (s, 2 H), 3.35 (*X), 2.93-2.82 (m, 1 H), 2.63-2.55 (m, 2 H), 2.35 (t, J = 5.80 Hz, 2 H), 2.06-1.97 (m, 1 H,), 1.64 (m, 4 H) ppm; 13C NMR (75.5 MHz, DMSO-d6): δ = 173.3, 171.6, 170.6, 169.4, 167.8, 162.6, 161.4, 152.5, 151.9 (*X_2), 146.8, 139.2, 136.7, 132.7, 132.5, 129.8, 128.1, 124.3 (*X_2), 123.7 (*X_2), 123.5, 121.5, 120.3, 119.7, 117.6, 110.9, 110.7, 109.5, 49.0, 47.1, 42.0, 36.5, 31.4, 28.8, 22.9, 22.6 ppm; HPLC: 17.47 min, start at 0.1 % B; MS calcd. m/z 846.1866, found m/z 847.1939 $[M+H]^+$).

6-((2-(2,6-Dioxopiperidin-3-yl)-1,3-dioxoisoindolin-4-yl)amino)-N-(4-((6-oxo-2-((2-oxo-2-((6-(trifluoromethyl)benzo[d]thiazol-2-yl)amino)ethyl)thio)pyrimidin-1(6H)-yl)methyl)phenyl)hexanamide (P1d)
Synthesis was done according to the described procedure using intermediate **L6d** (1.00 equiv. 0.16 mmol, 63 mg). The product was afforded as a yellow solid (7.1 % yield; 1H NMR (300 MHz, DMSO-d6): δ = 12.93 (s, 1 H), 11.10 (s, 1 H), 9.93 (s, 1 H), 8.44

*X = Signals were detected via COSY spectra
*X_2 = Signals were detected via HSQC and/or HMBC spectra

(s, 1 H), 7.88 (d, J = 8.5 Hz, 1 H), 7.79 (d, J = 6.5 Hz, 1 H), 7.72 (dd, J = 8.2 Hz, 1.3 Hz, 1 H), 7.59-7.53 (m, 3 H), 7.21 (d, J = 8.6 Hz, 2 H), 7.09 (d, J = 8.6 Hz, 1 H), 7.01 (d, J = 7.0 Hz, 1 H), 6.55 (t, J = 6.0 Hz, 1 H), 6.25 (d, J = 6.5 Hz, 1 H), 5.21 (s, 2 H), 5.05 (dd, J = 12.8 Hz, 5.5 Hz, 1 H), 4.29 (s, 2 H), 3.31 (*X), 2.93-2.82 (m, 1 H), 2.62-2.53 (m, 2 H), 2.31 (t, J = 7.6 Hz, 2 H), 2.06-1.98 (m, 1 H), 1.667-1.55 (m, 4 H), 1.42-1.33 (m, 2 H) ppm; 13C NMR (75.5 MHz, DMSO-d6): δ = 173.3, 171.7, 170.6, 169.4, 167.8, 162.8, 161.4, 152.6, 152.0 (*X_2), 146.9, 139.2, 136.8, 132.7, 129.7, 128.1, 124.4 (*X_2), 123.7 (*X_2), 123.3, 121.2, 120.2, 119.6, 117.7, 110.8, 110.6, 109.5, 49.0, 47.1, 42.2, 36.8, 31.4, 29.0, 26.4, 25.3, 22.6 ppm; HPLC: 17.95 min, start at 0.1 % B; MS calcd. m/z 860.2022, found m/z 861.2085 $[M+H]^+$).

3-((2-(2,6-Dioxopiperidin-3-yl)-1,3-dioxoisoindolin-5-yl)amino)-N-(4-((6-oxo-2-((2-oxo-2-((6-(trifluoromethyl)benzo[d]thiazol-2-yl)amino)ethyl)thio)pyrimidin-1(6H)-yl)methyl)phenyl)propanamide (P2a)
Synthesis was done according to the described procedure using intermediate **L8a** (1.00 equiv. 0.19 mmol, 65 mg). The product was afforded as a yellow solid (3.4 % yield; 1H NMR (300 MHz, DMSO-d6): δ = 12.91 (s, 1 H), 11.06 (s, 1 H), 10.04 (s, 1 H), 8.49 (s, 1 H), 7.92 (d, J = 8.5 Hz, 1 H), 7.79 (d, J = 6.5 Hz, 1 H), 7.75 (dd, J = 8.6 Hz, 1.3 Hz, 1 H), 7.61-7.56 (m, 3 H), 7.26-7.20 (m, 3 H), 7.01 (d, J = 1.8 Hz, 1 H), 6.90 (dd, J = 8.4 Hz, 2.0 Hz, 1 H), 6.25 (d, J = 6.5 Hz, 1 H), 5.22 (s, 2 H), 5.03 (dd, J = 12.8 Hz, 5.4 Hz, 1 H), 4.31 (s, 2 H), 3.51 (q, J = 6.0 Hz, 2 H), 2.93-2.82 (m, 1 H), 2.63 (t, J = 6.5 Hz, 1 H), 2.60-2.52 (m, 2 H), 2.04-1.95 (m, 1 H) ppm; 13C NMR (75.5 MHz, DMSO-d6): δ = 173.3, 170.6, 169.8, 168.2, 167.8, 167.6, 162.6, 161.6, 161.4, 154.7, 152.5, 151.8, 139.1, 134.7, 132.5, 129.9, 128.1, 125.6, 124.3 (d, *J* = 32.1 Hz), 123.7, 123.5, 121.5, 120.4, 119.7, 116.7, 116.1, 110.7, 105.7, 49.1, 47.1, 39.1, 36.4, 36.0, 31.5, 22.7 ppm; HPLC: 16.33 min, start at 0.1 % B; MS calcd. m/z 818.16, found m/z 817.1396 $[M-H]^-$).

6-((2-(2,6-Dioxopiperidin-3-yl)-1,3-dioxoisoindolin-5-yl)amino)-N-(4-((6-oxo-2-((2-oxo-2-((6-(trifluoromethyl)benzo[d]thiazol-2-yl)amino)ethyl)thio)pyrimidin-1(6H)-yl)methyl)phenyl)hexanamide (P2d)
Synthesis was done according to the described procedure using intermediate **L8d** (1.00 equiv. 0.17 mmol, 68 mg). The product was afforded as a yellow solid (11.3 % yield; 1H NMR (300 MHz, DMSO-d6): δ = 12.93 (s, 1 H), 11.06 (s, 1 H), 9.93 (s, 1 H), 8.48 (s, 1 H), 7.92 (d, J = 8.5 Hz, 1 H), 7.79 (d, J = 6.5 Hz, 1 H), 7.74 (dd, J = 8.6 Hz, 1.6 Hz, 1 H), 7.60-7.54 (m, 3 H), 7.23 (d, J = 8.6 Hz, 2 H), 7.12 (t, J = 5.3 Hz, 1 H), 6.95 (d, J = 1.7 Hz, 1 H), 6.85 (dd, J = 8.4 Hz, 1.9 Hz, 1 H), 6.26 (d, J = 6.5 Hz, 1 H), 5.22 (s, 2 H), 5.05 (dd, J = 12.9 Hz, 5.4 Hz, 1 H), 4.32 (s, 2 H), 3.17 (q, J = 5.7 Hz, 2 H), 2.93-2.82 (m, 1 H), 2.64-2.55 (m, 2 H), 2.32 (t, J = 7.3 Hz, 2 H), 2.04-1.95 (m, 1 H), 1.69-1.56 (m, 4 H), 1.46-1.37 (m, 2 H) ppm; 13C NMR (75.5 MHz, DMSO-d6): δ = 173.3, 171.7, 170.7, 168.2, 167.9, 167.6, 162.6, 161.8, 161.4, 154.9, 152.5, 151.8, 139.3, 134.7, 132.5, 129.7, 128.1, 125.6, 124.2 (d, *J* = 32.2 Hz), 123.7, 123.4, 121.4, 120.4, 119.6, 116.3, 115.7, 110.7, 108.5, 49.1, 47.1, 42.8, 36.8, 36.5, 31.5, 28.5, 26.7, 25.3, 22.7 ppm; HPLC: 17.26 min, start at 0.1 % B; MS calcd. m/z 860.2022, found m/z 861.2086 $[M+H]^+$).

3-(2-(2-(2-((2-(2,6-Dioxopiperidin-3-yl)-1,3-dioxoisoindolin-4-yl)oxy)ethoxy)ethoxy)ethoxy)-N-(4-((6-oxo-2-((2-oxo-2-((6-(trifluoromethyl)benzo[d]thiazol-2-yl)amino)ethyl)thio)pyrimidin-1(6H)-yl)methyl)phenyl)propanamide (P3a)
Synthesis was done according to the described procedure using intermediate **L13a** (1.00 equiv. 0.28 mmol, 111 mg). The product was afforded as a white solid (4.5 % yield; 1H NMR (300 MHz, DMSO-d6): δ = 12.91 (s, 1 H), 11.10 (s, 1 H), 9.98 (s, 1 H), 8.48 (s, 1 H), 7.92 (d, J = 8.5 Hz, 1 H), 7.79 (d, J = 6.5 Hz, 1 H), 7.76-7.72 (m, 2 H), 7.55 (d, J = 8.6 Hz, 2 H), 7.51 (d, J = 8.5 Hz, 1 H) 7.43 (d, J = 7.2 Hz, 1 H) 7.22 (d, J = 8.6 Hz, 2 H), 6.25 (d, J = 6.5 Hz, 1 H), 5.21 (s, 2 H), 5.07 (dd, J = 12.7 Hz, 5.4 Hz, 1 H), 4.36-4.32 (m, 2 H), 4.31 (s, 2 H), 3.84-3.76 (m, 4 H), 2.93-2.82 (m, 1 H), 2.63-2.53 (m, 4 H), 2.05-1.97 (m, 1 H) ppm; 13C NMR (75.5 MHz, DMSO-d6): δ = 173.3, 170.4, 169.6, 167.8, 167.3, 165.7, 162.6, 161.4, 156.3, 152.5, 151.8 (*X_2), 139.1, 137.4, 133.7, 132.5, 129.8, 128.1, 124.2 (*X_2), 123.7 (*X_2), 123.5 (*X_2), 121.5, 120.6, 120.4, 119.6, 116.8, 115.9, 110.7, 69.2, 69.1, 67.5, 49.2, 47.1, 37.6, 36.4, 31.4, 22.5 ppm; HPLC: 15.73 min, start at 0.1 % B; MS calcd. m/z 863.1655, found m/z 864.1719 $[M+H]^+$).

3-(2-(2-((2-(2,6-Dioxopiperidin-3-yl)-1,3-dioxoisoindolin-4-yl)oxy)ethoxy)ethoxy)-N-(4-((6-oxo-2-((2-oxo-2-((6-(trifluoromethyl)benzo[d]thiazol-2-yl)amino)ethyl)thio)pyrimidin-1(6H)-yl)methyl)phenyl)propanamide (P3b)
Synthesis was done according to the described procedure using intermediate **L13b** (1.00 equiv. 0.37 mmol, 159 mg). The product was afforded as a white solid (10.2 % yield; 1H NMR (300 MHz, DMSO-d6): δ = 12.90 (s, 1 H), 11.09 (s, 1 H), 9.97 (s, 1 H), 8.48 (s, 1 H), 7.92 (d, J = 8.4 Hz, 1 H), 7.80-7.73 (m, 3 H), 7.57 (d, J = 8.5 Hz, 2 H), 7.48 (d, J = 8.5 Hz, 1 H) 7.44 (d, J = 7.2 Hz, 1 H) 7.22 (d, J = 8.6 Hz, 2 H), 6.25 (d, J = 6.5 Hz, 1 H), 5.20 (s, 2 H), 5.07 (dd, J = 12.8 Hz 5.3 Hz, 1 H), 4.31 (s, 2 H), 4.30-4.27 (m, 2 H), 3.80-3.72 (m, 2 H), 3.69 (t, J = 6.2 Hz, 2 H), 3.65-3.62 (m, 2 H), 3.56-3.52 (m, 2 H), 2.92-2.82 (m, 1 H), 2.57-2.52 (m, 2 H), 2.51 (*X) 2.05-1.97 (m, 1 H) ppm; 13C NMR (75.5 MHz, DMSO-d6): δ = 173.3, 170.4, 169.7, 167.7, 167.3, 165.7, 162.5, 161.5, 161.5, 156.3, 152.5, 151.8, 139.1, 137.4, 133.7, 132.5, 129.8, 128.1, 124.3 (*X_2), 123.5, 121.5, 120.5, 119.6, 116.8, 115.9, 110.7, 70.5, 70.1, 69.3, 69.1, 67.1, 49.2, 47.1, 37.6, 36.3, 31.4, 22.5 ppm; HPLC: 15.91 min, start at 0.1 % B; MS calcd. m/z 907.1917, found m/z 908.1981 $[M+H]^+$).

3-(2-(2-(2-((2-(2,6-Dioxopiperidin-3-yl)-1,3-dioxoisoindolin-4-yl)oxy)ethoxy)ethoxy)ethoxy)-N-(4-((6-oxo-2-((2-oxo-2-((6-(trifluoromethyl)benzo[d]thiazol-2-yl)amino)ethyl)thio)pyrimidin-1(6H)-yl)methyl)phenyl)propanamide (P3c)
Synthesis was done according to the described procedure using intermediate **L13c** (1.00 equiv. 0.30 mmol, 141 mg). The product was afforded as a white solid (7.5 % yield; 1H NMR (300 MHz, DMSO-d6): δ = 12.82 (s, 1 H), 11.09 (s, 1 H), 9.98 (s, 1 H), 8.38 (s, 1 H), 7.84 (d, J = 6.5 Hz, 1 H), 7.81-7.76 (m, 2 H), 7.69 (dd, J = 8.3 Hz, 1.27 Hz, 1 H), 7.57 (d, J = 8.4 Hz, 2 H), 7.50 (d, J = 8.5 Hz, 1 H) 7.44 (d, J = 7.2 Hz, 1 H) 7.22 (d, J = 8.4 Hz, 2 H), 6.24 (d, J = 6.5 Hz, 1 H), 5.21 (s, 2 H), 5.08 (dd, J = 12.8 Hz, 5.4 Hz, 1 H), 4.34-4.29 (m, 2 H), 4.26 (s, 2 H), 3.79-3.74 (m, 2 H), 3.67 (t, J = 6.2 Hz, 2 H), 3.63-3.58 (m, 2 H), 3.53-3.51 (m, 2 H), 3.51-3.48 (m, 4 H), 2.93-2.82 (m, 1 H), 2.63-2.54 (m, 2 H), 2.51 (*X) 2.06-1.98 (m, 1 H) ppm; 13C NMR (75.5 MHz, DMSO-d6): δ

*X = Signals were detected via COSY spectra
*X_2 = Signals were detected via HSQC and/or HMBC spectra

= 173.3, 170.4, 169.7, 167.7, 167.3, 165.7, 162.9, 161.4, 156.3, 152.6, 152.2, 139.1, 137.4, 133.7, 132.7, 129.9, 128.1, 124.3 (*X_2), 123.7 (*X_2), 123.1, 120.9, 120.5, 120.0 119.6, 116.8, 115.8, 110.5, 70.6, 70.3, 70.1, 69.3, 69.1, 67.1, 49.2, 47.0, 37.6, 36.3, 31.4, 22.5 ppm; HPLC: 15.86 min, start at 0.1 % B; MS calcd. m/z 951.2179, found m/z 950.2100 $[M-H]^-$).

3-(3-((2-(2,6-Dioxopiperidin-3-yl)-1,3-dioxoisoindolin-4-yl)oxy)propoxy)-N-(4-((6-oxo-2-((2-oxo-2-((6-(trifluoromethyl)benzo[d]thiazol-2-yl)amino)ethyl)thio)pyrimidin-1(6H)-yl)methyl)phenyl)propanamide (P3d)
Synthesis was done according to the described procedure using intermediate **L13d** (1.00 equiv. 0.30 mmol, 119 mg). The product was afforded as a white solid (3.5 % yield; 1H NMR (300 MHz, DMSO-d6): δ = 12.92 (s, 1 H), 11.10 (s, 1 H), 9.98 (s, 1 H), 8.49 (s, 1 H), 7.93 (d, J = 8.5 Hz, 1 H), 7.80 (d, J = 6.5 Hz, 1 H), 7.75 (dd, J = 8.5 Hz, 1.4 Hz, 1 H), 7.72 (dd, J = 8.7 Hz, 7.5 Hz, 1 H), 7.56 (d, J = 8.5 Hz, 2 H), 7.42 (d, J = 2.5 Hz, 1 H) 7.40 (d, J = 3.7 Hz, 1 H) 7.22 (d, J = 8.6 Hz, 2 H), 6.26 (d, J = 6.5 Hz, 1 H), 5.22 (s, 2 H), 5.08 (dd, J = 12.8 Hz, 5.4 Hz, 1 H), 4.33 (s, 2 H), 4.23 (t, J = 6.2 Hz, 2 H), 3.70 (t, J = 6.1 Hz, 2 H), 3.61 (t, J = 6.1 Hz, 2 H), 2.93-2.82 (m, 1 H), 2.62-2.52 (m, 4 H), 2.05-1.96 (m, 3 H) ppm; 13C NMR (75.5 MHz, DMSO-d6): δ = 173.2, 170.4, 169.8, 167.7, 167.3, 165.8, 162.5, 161.5, 161.3, 156.3, 152.5, 151.8, 139.1, 137.4, 133.7, 132.5, 129.9, 128.1, 124.3 (d, *J* = 32.0 Hz), 123.7, 123.5, 121.5, 120.4, 120.1, 119.6, 116.8, 115.7, 110.7, 66.8, 66.7, 66.3, 49.2, 47.1, 37.6, 36.3, 31.4, 29.2, 22.5 ppm; HPLC: 16.10 min, start at 0.1 % B; MS calcd. m/z 877.1812, found m/z 878.1884 $[M+H]^+$).

3-(2-((2-(2,6-Dioxopiperidin-3-yl)-1,3-dioxoisoindolin-5-yl)oxy)ethoxy)-N-(4-((6-oxo-2-((2-oxo-2-((6-(trifluoromethyl)benzo[d]thiazol-2-yl)amino)ethyl)thio)pyrimidin-1(6H)-yl)methyl)phenyl)propenamide (P4a)
Synthesis was done according to the described procedure using intermediate **L15a** (1.00 equiv. 0.20 mmol, 90 mg). The product was afforded as a white solid (11.0 % yield; 1H NMR (300 MHz, DMSO-d6): δ = 12.89 (s, 1 H), 11.10 (s, 1 H), 10.0 (s, 1 H), 8.48 (s, 1 H), 7.92 (d, *J* = 8.5 Hz, 1 H), 7.80-7.77 (m, 2 H), 7.74 (dd, , *J* = 8.6 Hz, 1.6 Hz, 2 H), 7.56 (d, *J* = 8.6 Hz, 2 H), 7.43 (d, *J* = 2.2 Hz, 1 H), 7.32 (dd, *J* = 8.3 Hz, 2.3 Hz, 1 H), 7.22 (d, *J* = 8.6 Hz, 2 H), 6.25 (d, *J* = 6.5 Hz, 1 H), 5.21 (s, 2 H), 5.11 (dd, *J* = 12.9 Hz, 5.4 Hz, 1 H), 4.33-4.28 (m, 4 H), 3.81-3.76 (m, 4 H), 2.94-2.83 (m, 1 H), 2.66-2.52 (m, 4 H), 2.09-2.00 (m, 1 H) ppm; 13C NMR (75.5 MHz, DMSO-d6): δ = 173.2, 170.4, 169.6, 167.7, 167.3, 164.4, 162.6, 161.6, 161.4, 152.5, 151.8, 139.1, 134.4, 132.5, 129.9, 128.1, 125.7, 124.3 (d, *J* = 31.9 Hz), 123.7, 123.5, 121.5, 121.3, 120.4, 119.6, 110.7, 109.4, 68.9, 68.8, 67.2, 49.4, 47.1, 37.5, 36.3, 31.4, 22.5 ppm; HPLC: 15.95 min, start at 0.1 % B; MS calcd. m/z 863.1655, found m/z 862.1549 $[M-H]^-$)

3-(2-(2-((2-(2,6-Dioxopiperidin-3-yl)-1,3-dioxoisoindolin-5-yl)oxy)ethoxy)ethoxy)-N-(4-((6-oxo-2-((2-oxo-2-((6-(trifluoromethyl)benzo[d]thiazol-2-yl)amino)ethyl)thio)pyrimidin-1(6H)-yl)methyl)phenyl)propenamide (P4b)
Synthesis was done according to the described procedure using intermediate **L15b** (1.00 equiv. 0.20 mmol, 99 mg). The product was afforded as a white solid (9.5 % yield; 1H NMR (300 MHz, DMSO-d6): δ = 12.90 (s, 1 H), 11.11 (s, 1 H), 9.98 (s, 1 H), 8.48 (s, 1 H), 7.92 (d, ^3J = 8.5 Hz, 1 H), 7.81 (d, *J* = 8.3 Hz, 1 H), 7.78 (d, *J* = 6.8 Hz, 1 H), 7.74 (dd, *J* = 8.6 Hz, 1.6 Hz, 1 H), 7.58 (d, *J* = 8.6 Hz, 2 H), 7.42 (d, *J* = 2.2 Hz, 1 H), 7.32 (dd, *J* = 8.3 Hz, 2.3 Hz, 1 H), 7.22 (d, *J* = 8.6 Hz, 2 H), 6.24 (d, *J* = 6.5 Hz, 1 H), 5.20 (s, 2 H), 5.11 (dd, *J* = 12.9 Hz, 5.4 Hz, 1 H), 4.31 (s, 2 H), 4.28-4.23 (m, 2 H), 3.78-3.74 (m, 2 H), 3.70 (t, *J* = 6.2 Hz, 2 H), 3.61-3.57 (m, 2 H), 3.56-3.52 (m, 2 H), 2.93-2.83 (m, 1 H), 2.63-2.52 (m, 4 H), 2.08-2.00 (m, 1 H) ppm; 13C NMR (75.5 MHz, DMSO-d6): δ = 173.2, 170.4, 169.7, 167.7, 167.3, 164.4, 162.5, 161.5, 161.3, 152.5, 151.8, 139.1, 134.4, 132.5, 129.8, 128.1, 125.7, 124.3 (d, *J* = 31.9 Hz), 123.7, 123.5, 121.5, 121.3, 120.4, 119.6, 110.7, 109.3, 70.3, 70.1, 69.1, 68.9, 67.1, 49.4, 47.1, 37.6, 36.3, 31.4, 22.5 ppm; HPLC: 16.03 min, start at 0.1 % B; MS calcd. m/z 907.1917, found m/z 906.1833 $[M-H]^-$).

3-(2-(2-(2-((2-(2,6-Dioxopiperidin-3-yl)-1,3-dioxoisoindolin-5-yl)oxy)ethoxy)ethoxy)ethoxy)-N-(4-((6-oxo-2-((2-oxo-2-((6-(trifluoromethyl)benzo[d]thiazol-2-yl)amino)ethyl)thio)pyrimidin-1(6H)-yl)methyl)phenyl)propenamide (P4c)
Synthesis was done according to the described procedure using intermediate **L15c** (1.00 equiv. 0.20 mmol, 108 mg). The product was afforded as a white solid (16.5 % yield; 1H NMR (300 MHz, DMSO-d6): δ = 12.90 (s, 1 H), 11.11 (s, 1 H), 9.97 (s, 1 H), 8.48 (s, 1 H), 7.92 (d, *J* = 6.5 Hz, 1 H), 7.81 (d, *J* = 8.3 Hz, 1 H), 7.78 (d, *J* = 6.5 Hz, 1 H), 7.74 (dd, *J* = 8.6 Hz, 1.6 Hz, 1 H), 7.57 (d, *J* = 8.6 Hz, 2 H), 7.43 (d, *J* = 2.2 Hz, 1 H), 7.34 (dd, *J* = 8.3 Hz, 2.3 Hz, 1 H), 7.22 (d, *J* = 8.6 Hz, 2 H), 6.24 (d, *J* = 6.5 Hz, 1 H), 5.21 (s, 2 H), 5.11 (dd, *J* = 12.9 Hz, 5.4 Hz, 1 H), 4.31 (s, 2 H), 4.29-4.25 (m, 2 H), 3.76-3.73 (m, 2 H), 3.68 (t, *J* = 6.2 Hz, 2 H), 3.57-3.54 (m, 2 H), 3.54-3.51 (m, 2 H), 3.51-3.48 (m, 4 H), 2.94-2.83 (m, 1 H), 2.63-2.51 (m, 4 H), 2.09-2.00 (m, 1 H) ppm; 13C NMR (75.5 MHz, DMSO-d6): δ = 173.2, 170.4, 169.7, 167.7, 167.3, 164.4, 162.5, 161.5, 161.3, 152.5, 151.8, 139.1, 134.4, 132.5, 129.8, 128.1, 125.7, 124.3 (d, *J* = 32.0 Hz), 123.7, 123.5, 121.5, 121.3, 120.4, 119.6, 110.7, 109.3, 70.4, 70.2, 69.1, 68.9, 67.1, 49.4, 47.1, 37.6, 36.3, 31.4, 22.5 ppm; HPLC: 16.52 min, start at 0.1 % B; MS calcd. m/z 951.2179, found m/z 950.2089 $[M-H]^-$).

3-(3-((2-(2,6-Dioxopiperidin-3-yl)-1,3-dioxoisoindolin-5-yl)oxy)propoxy)-N-(4-((6-oxo-2-((2-oxo-2-((6-(trifluoromethyl)benzo[d]thiazol-2-yl)amino)ethyl)thio)pyrimidin-1(6H)-yl)methyl)phenyl)propenamide (P4d)
Synthesis was done according to the described procedure using intermediate **L15d** (1.00 equiv. 0.20 mmol, 93 mg). The product was afforded as a white solid (18.3 % yield; 1H NMR (300 MHz, DMSO-d6): δ = 12.90 (s, 1 H), 11.11 (s, 1 H), 9.98 (s, 1 H), 8.49 (s, 1 H), 7.92 (d, ^3J = 6.5 Hz, 1 H), 7.81-7.77 (m, 2 H), 7.75 (dd, *J* = 8.6 Hz, 1.5 Hz, 1 H), 7.56 (d, *J* = 8.6 Hz, 2 H), 7.39 (d, *J* = 2.2 Hz, 1 H), 7.30 (dd, *J* = 8.3 Hz, 2.3 Hz, 1 H), 7.21 (d, *J* = 8.6 Hz, 2 H), 6.25 (d, *J* = 6.5 Hz, 1 H), 5.20 (s, 2 H), 5.12 (dd, *J* = 12.9 Hz, 5.4 Hz, 1 H), 4.31 (s, 2 H), 4.20 (t, *J* = 6.3 Hz, 2 H), 3.69 (t, *J* = 6.1 Hz, 2 H), 3.56 (t, *J* = 6.1 Hz, 2 H), 2.94-2.83 (m, 1 H), 2.63-2.52 (m, 4 H), 2.09-1.93 (m, 3 H) ppm; 13C NMR (75.5 MHz, DMSO-d6): δ = 173.2, 170.4, 169.7, 167.7, 167.3, 164.4, 162.5, 161.5, 161.3, 152.5, 151.8, 139.1, 134.4, 132.5, 129.8, 128.1, 125.8, 124.3 (d, *J* = 31.7 Hz), 123.8, 123.4, 121.5, 121.0, 120.4, 119.6, 110.7, 109.4, 66.9, 66.8, 66.4, 49.4, 47.1, 37.6, 36.3, 31.4, 29.2, 22.5 ppm; HPLC: 16.02 min, start at 0.1 % B; MS calcd. m/z 877.1812, found m/z 876.1743 $[M-H]^-$).

*X = Signals were detected via COSY spectra
*X_2 = Signals were detected via HSQC and/or HMBC spectra

Copies of NMR spectra of synthesized PROTAC compounds

Compound P1a

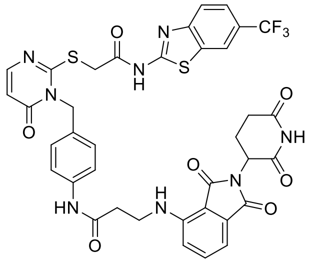

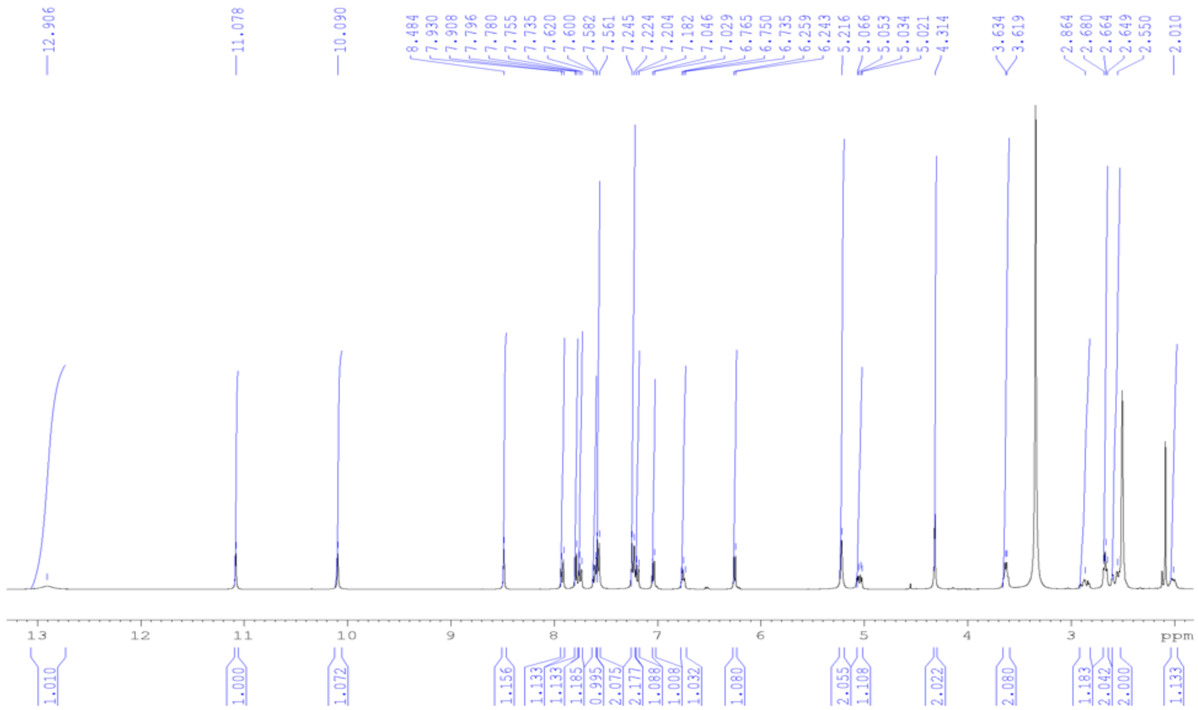

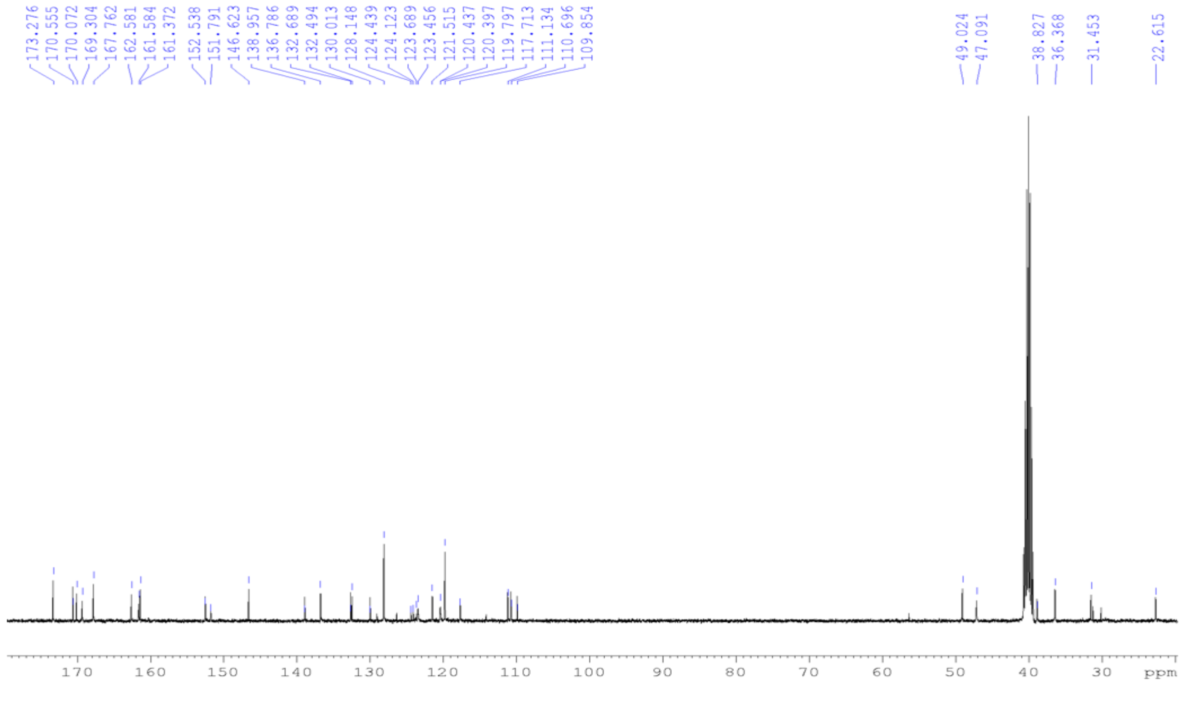

Figure S1 ^{1}H- and ^{13}C-NMR spectra of compound P1a

Compound P1b

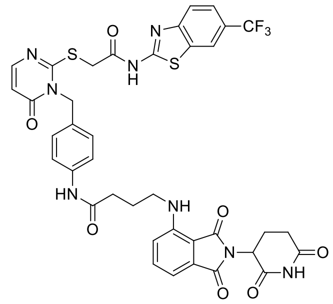

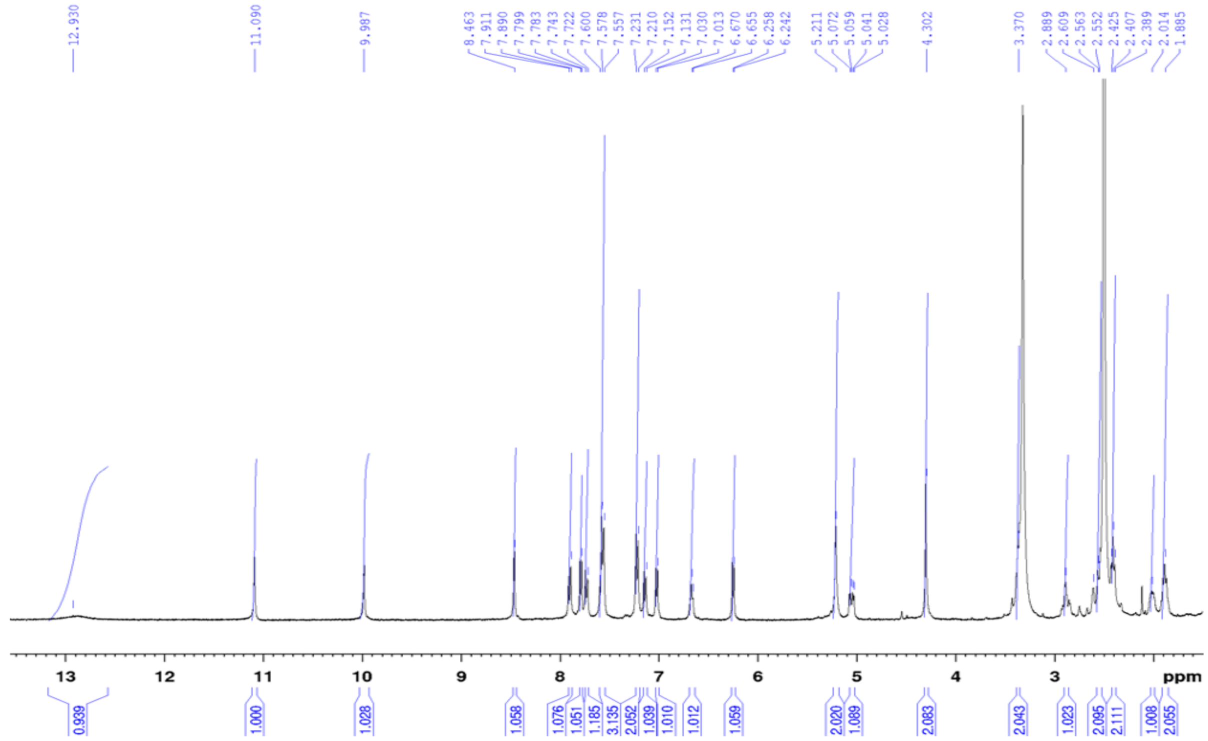

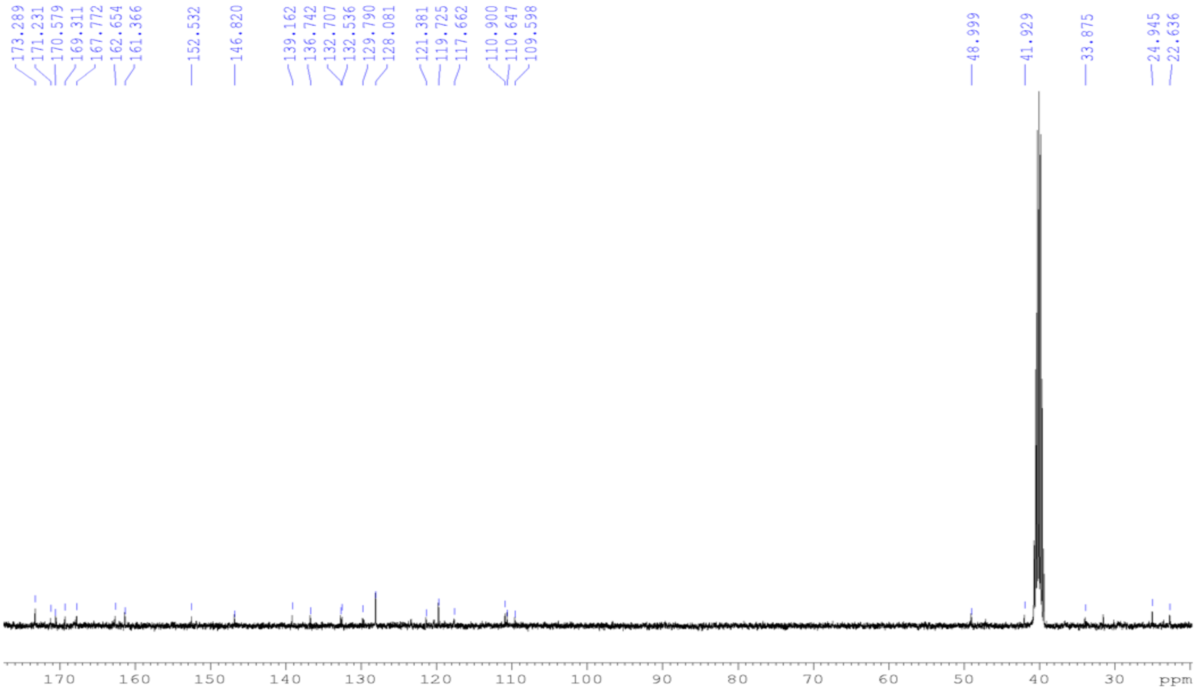

Figure S2 ^{1}H- and ^{13}C-NMR spectra of compound P1b

Compound P1c

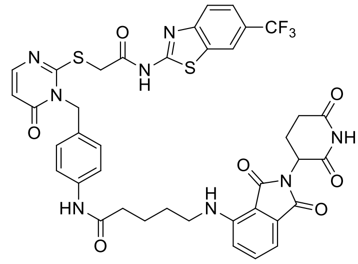

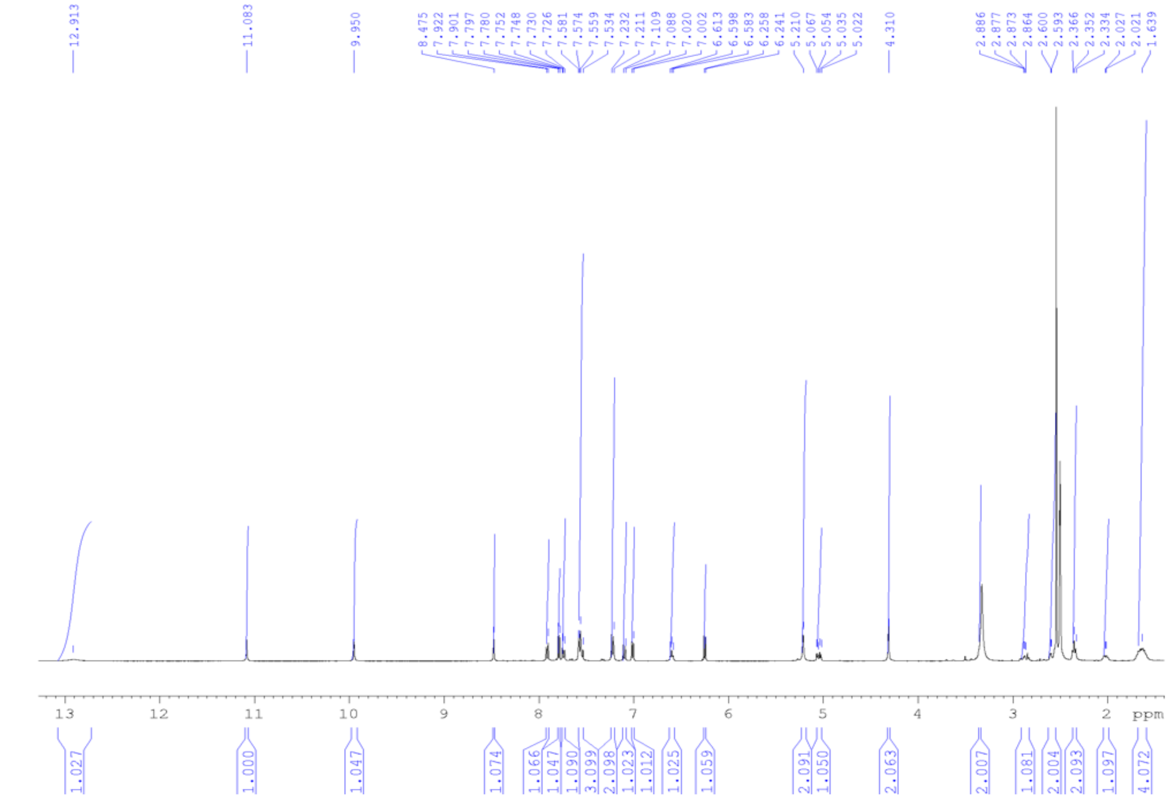

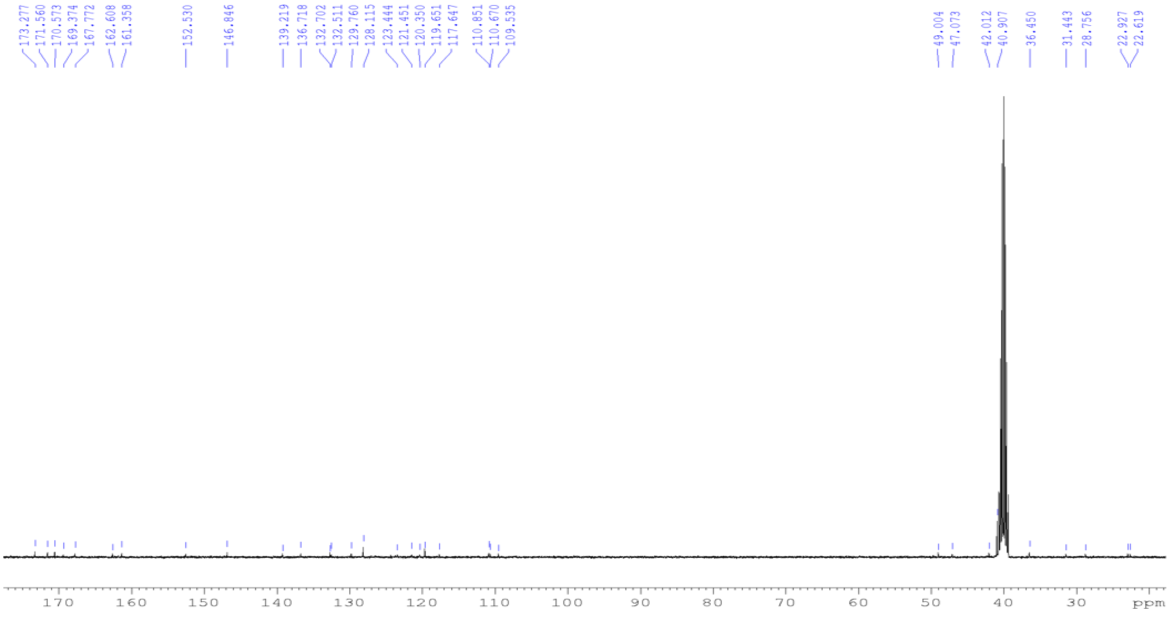

Figure S3 ^{1}H- and ^{13}C-NMR spectra of compound P1c

Compound P1d

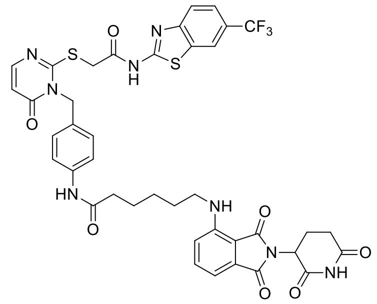

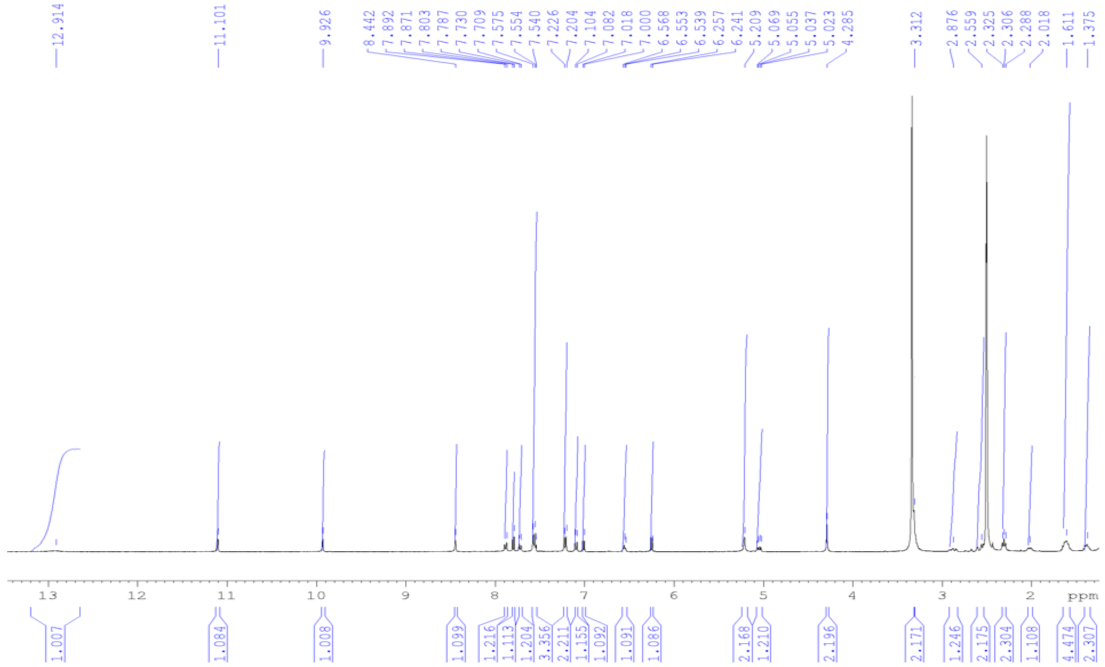

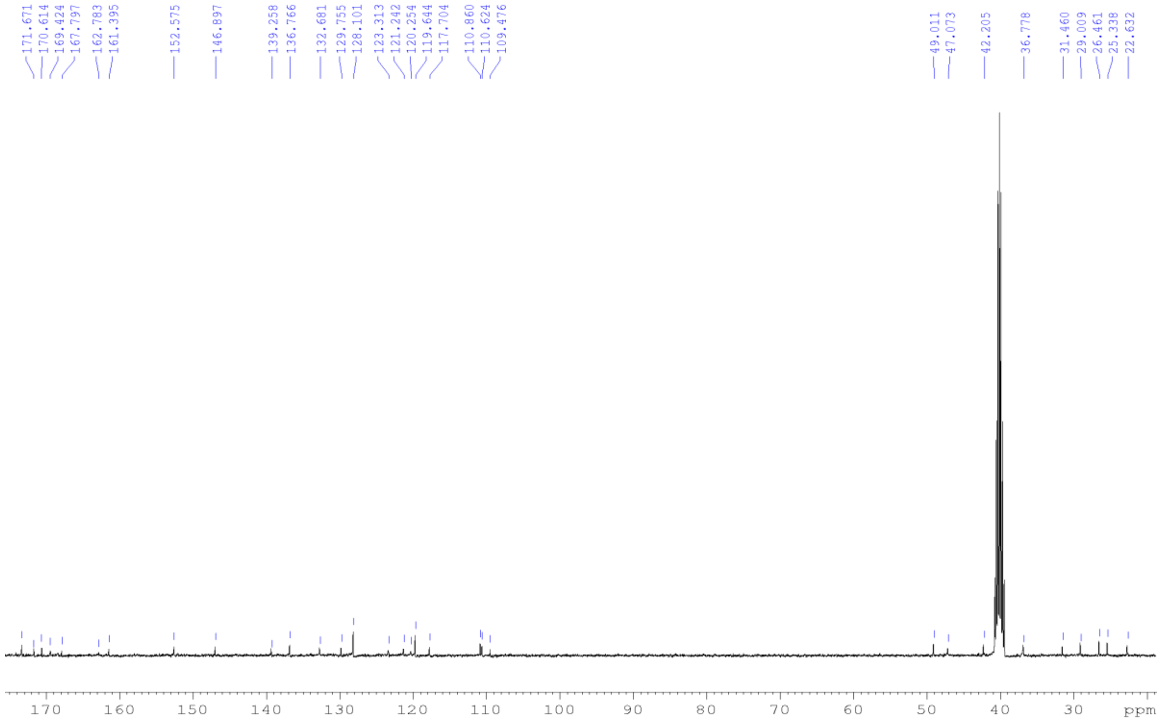

Figure S4 ^{1}H- and ^{13}C-NMR spectra of compound P1d

Compound P2a

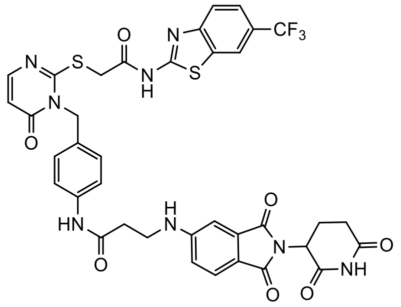

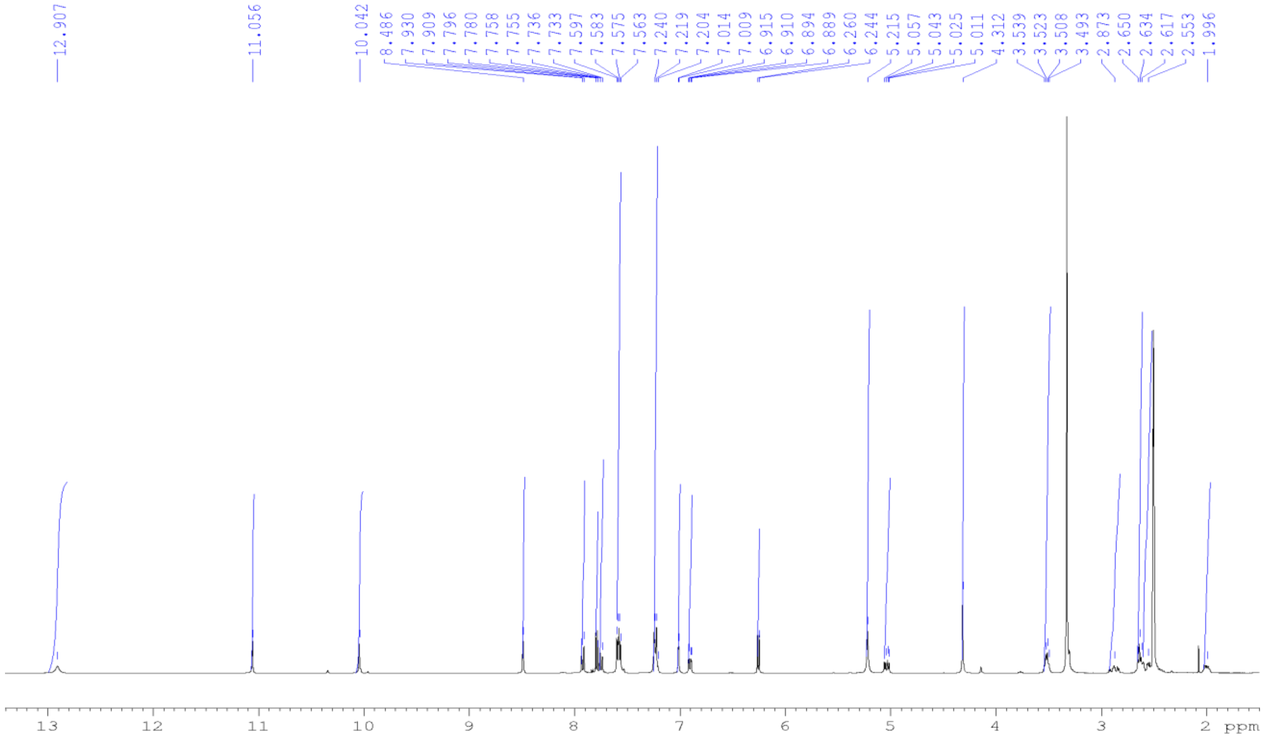

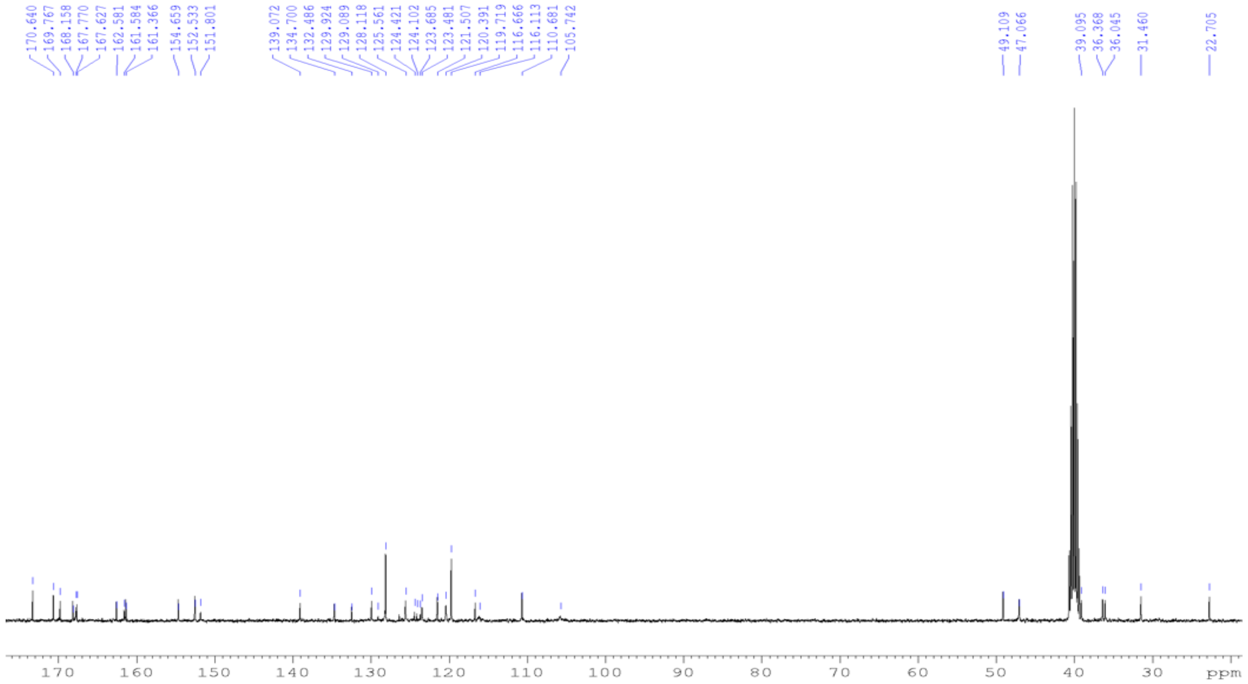

Figure S5 ^{1}H- and ^{13}C-NMR spectra of compound P2a

Compound P2d

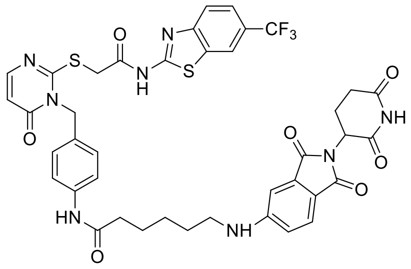

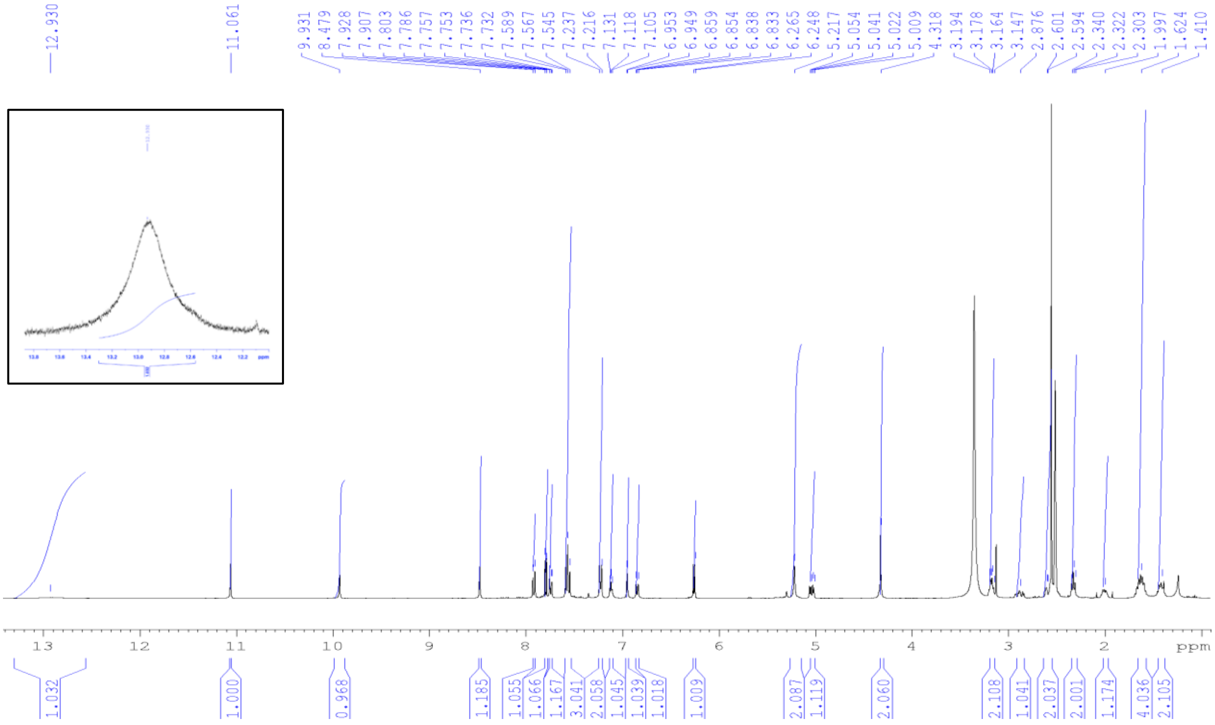

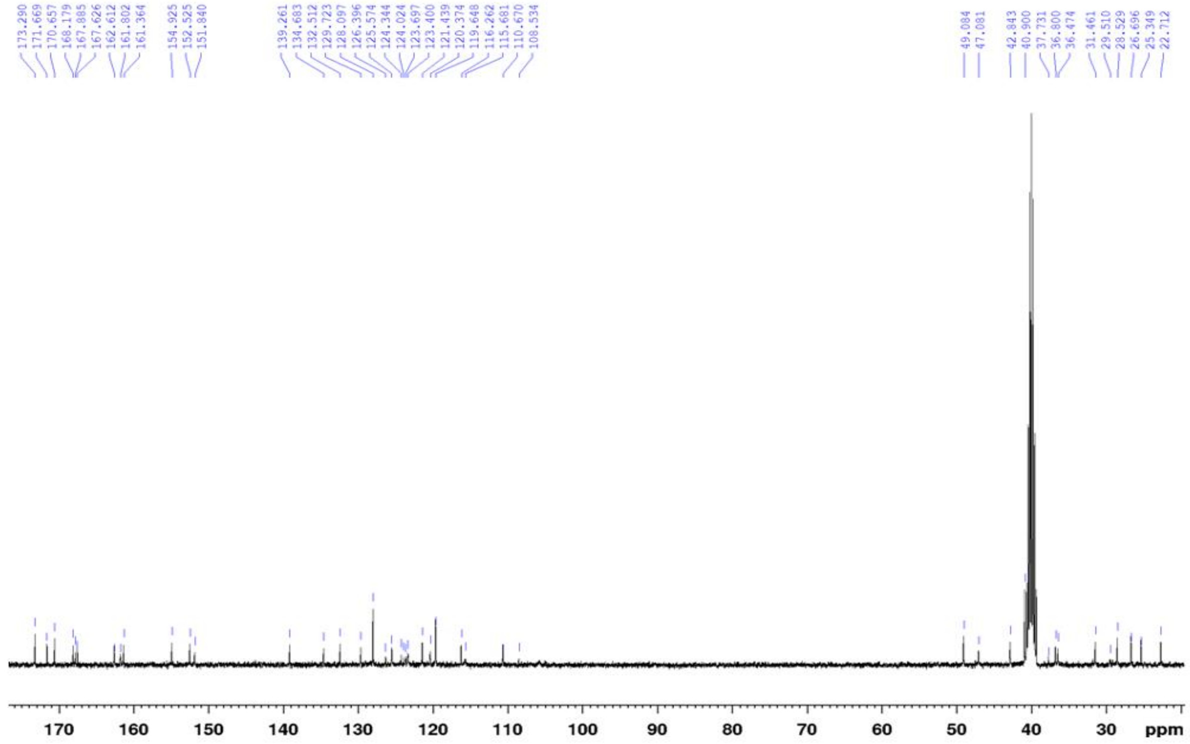

Figure S6 ^{1}H- and ^{13}C-NMR spectra of compound P2d

Compound P3a

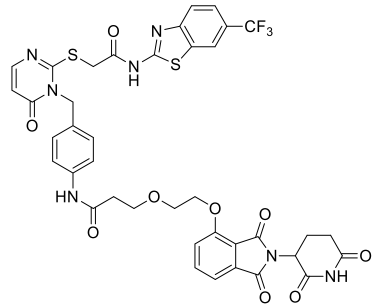

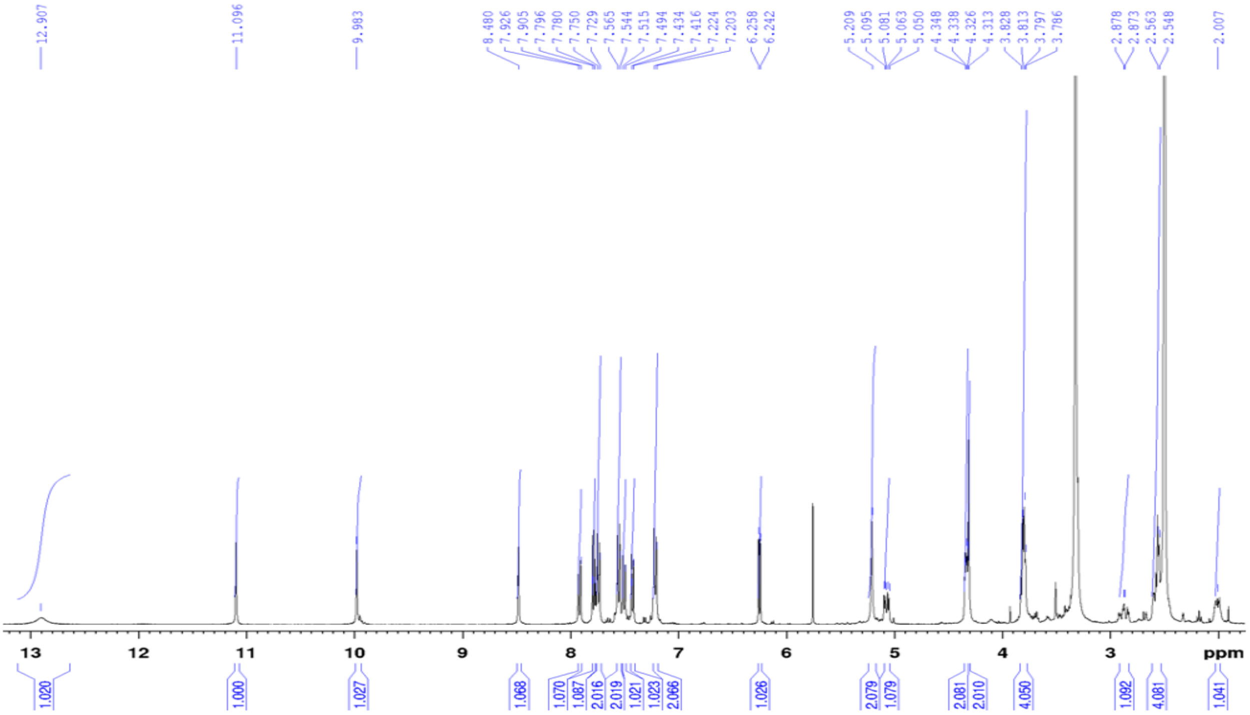

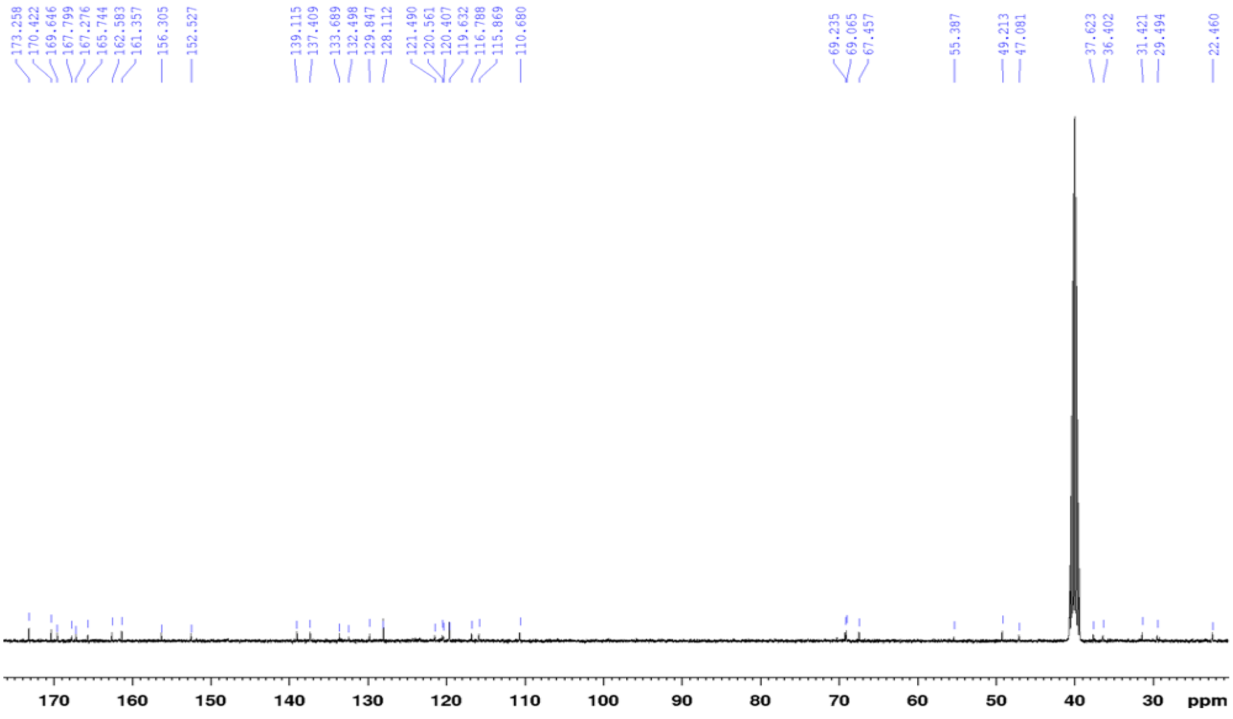

Figure S7 ¹H- and ¹³C-NMR spectra of compound P3a

Compound P3b

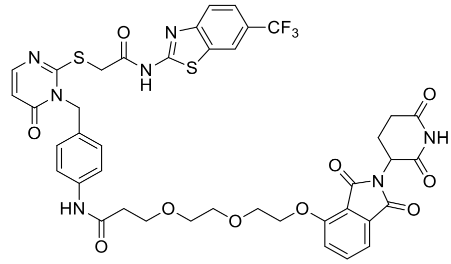

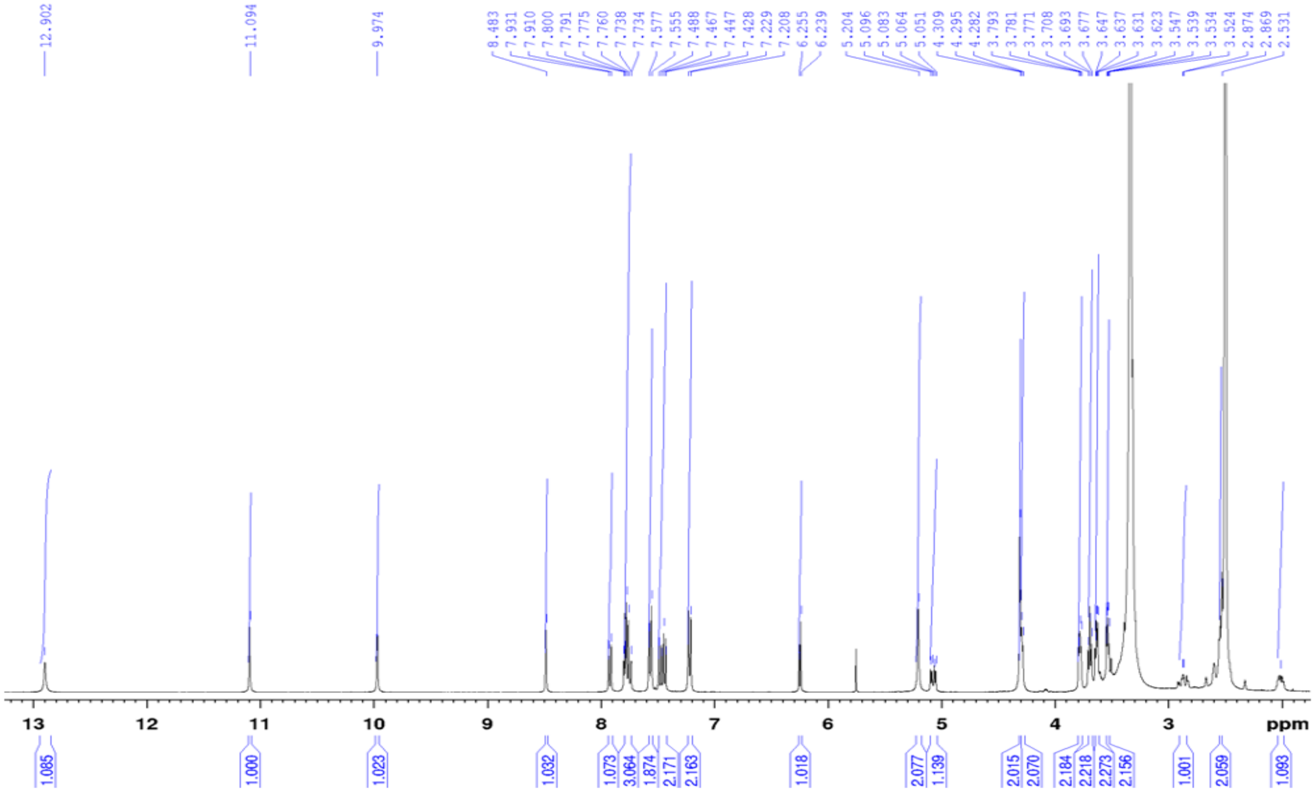

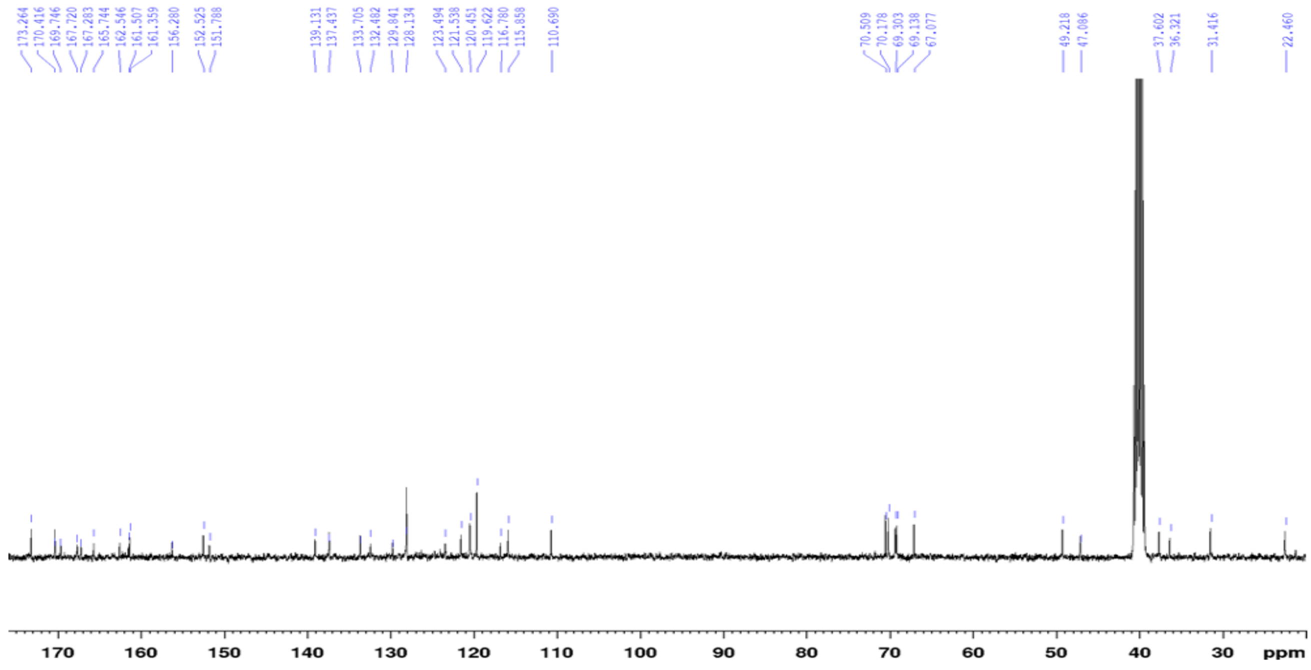

Figure S8 ^{1}H- and ^{13}C-NMR spectra of compound P3b

Compound P3c

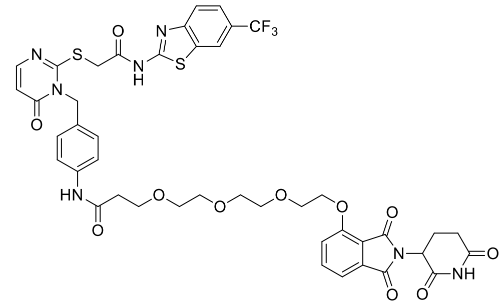

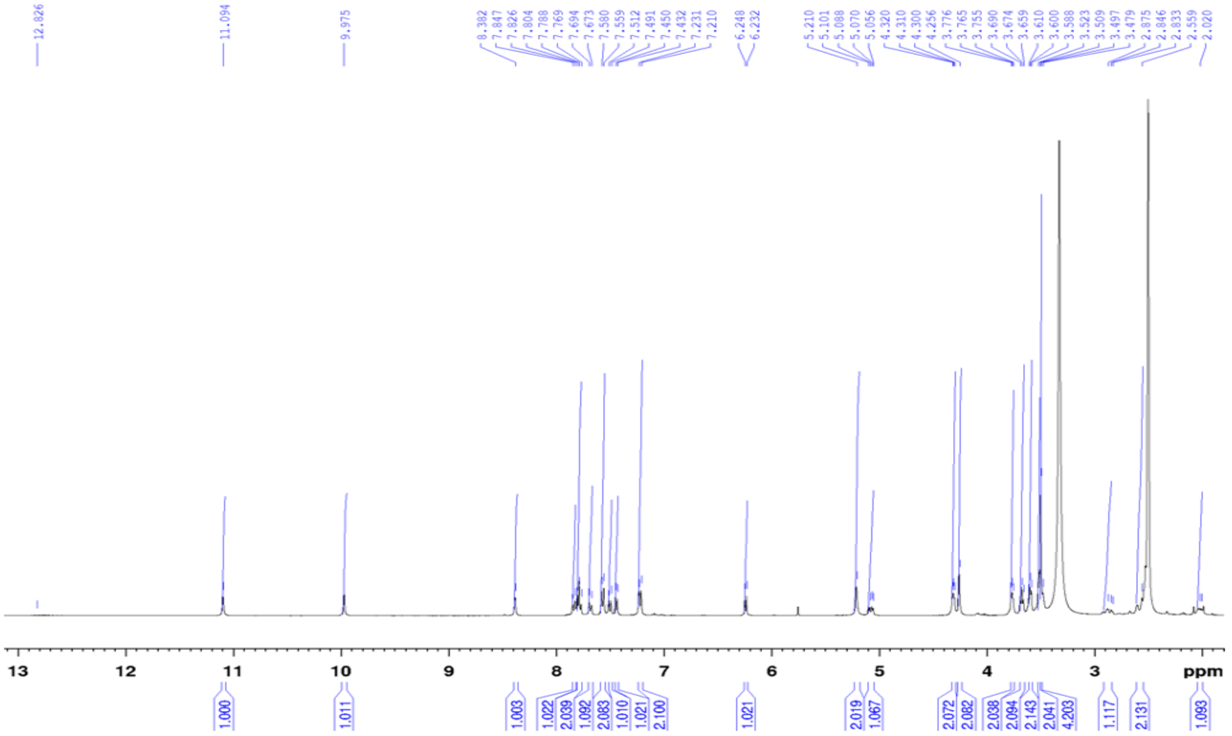

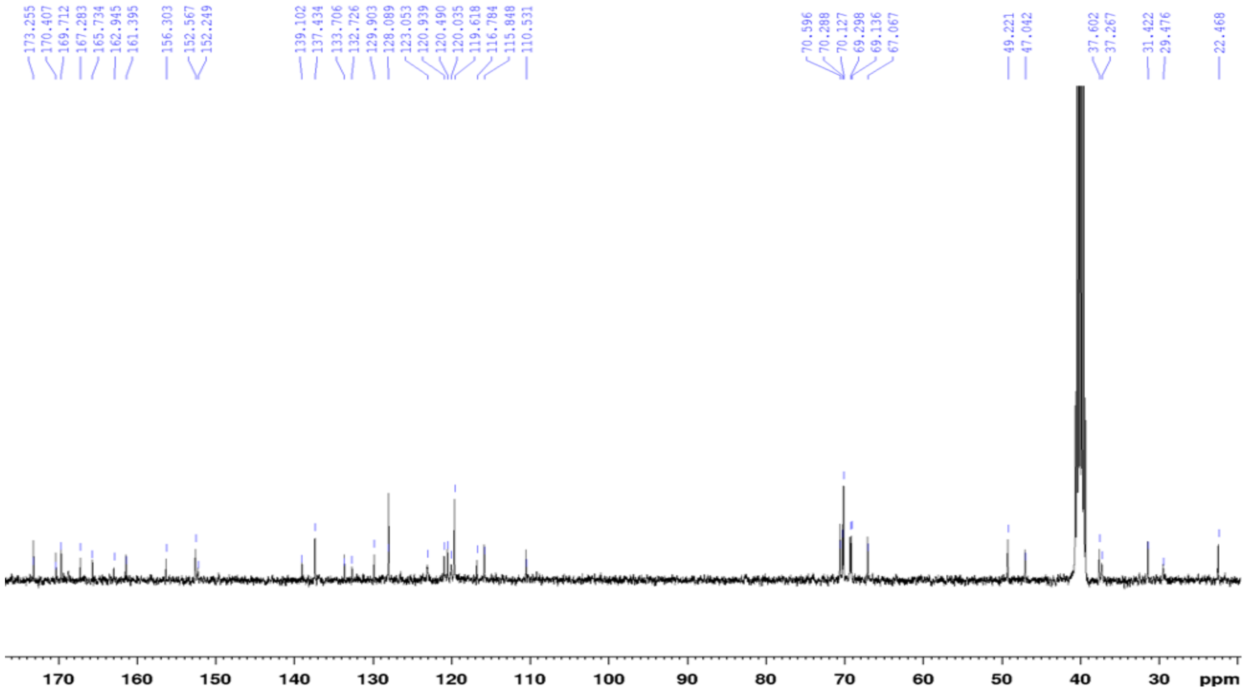

Figure S9 ^{1}H- and ^{13}C-NMR spectra of compound P3c

Compound P3d

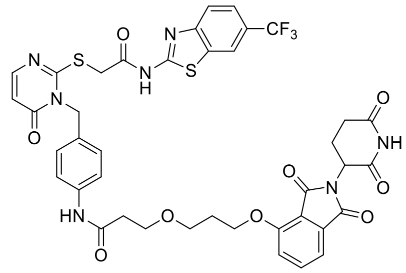

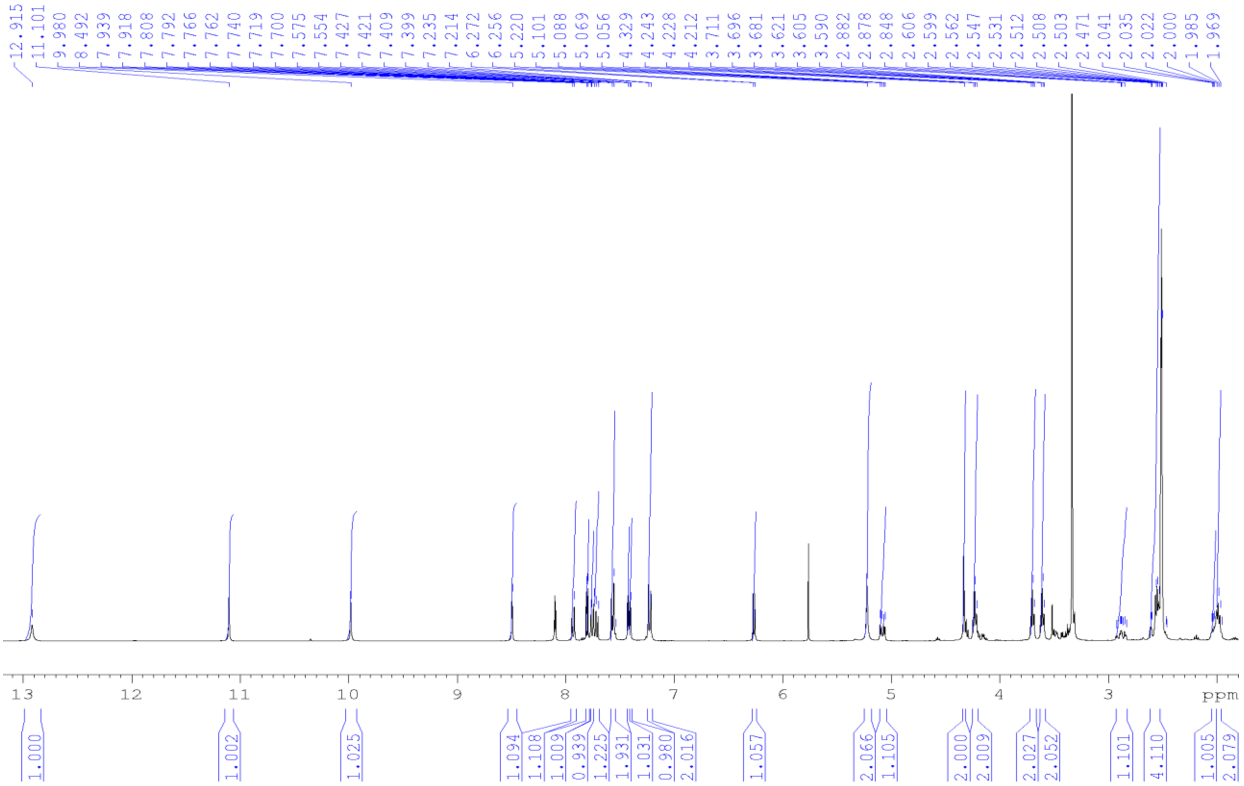

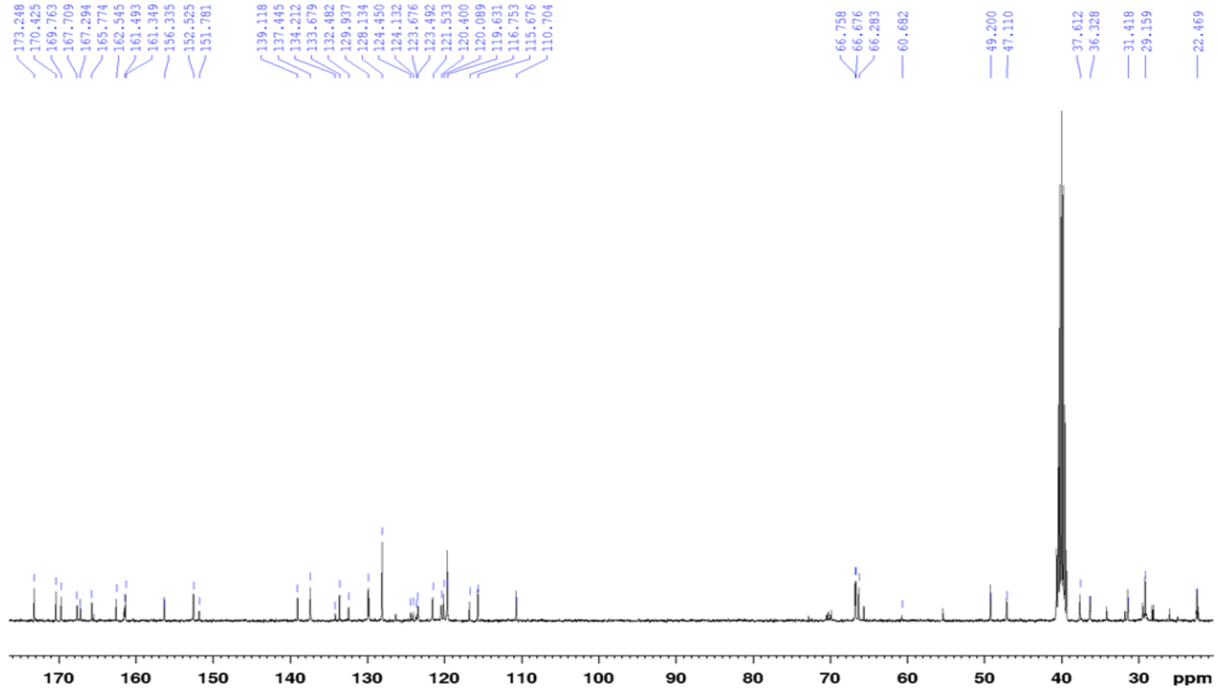

Figure S10 ^{1}H- and ^{13}C-NMR spectra of compound P3d

Compound P4a

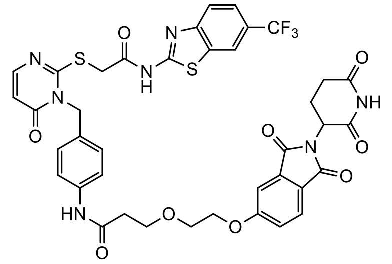

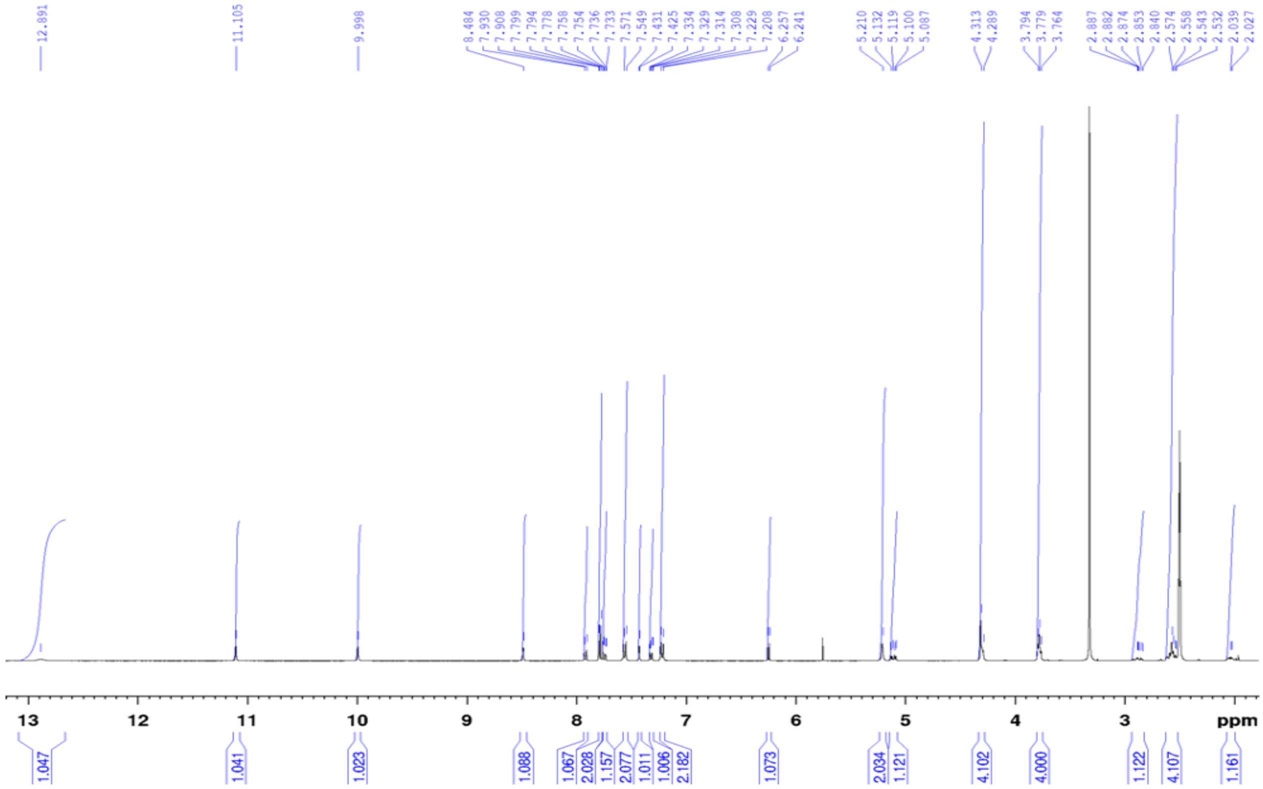

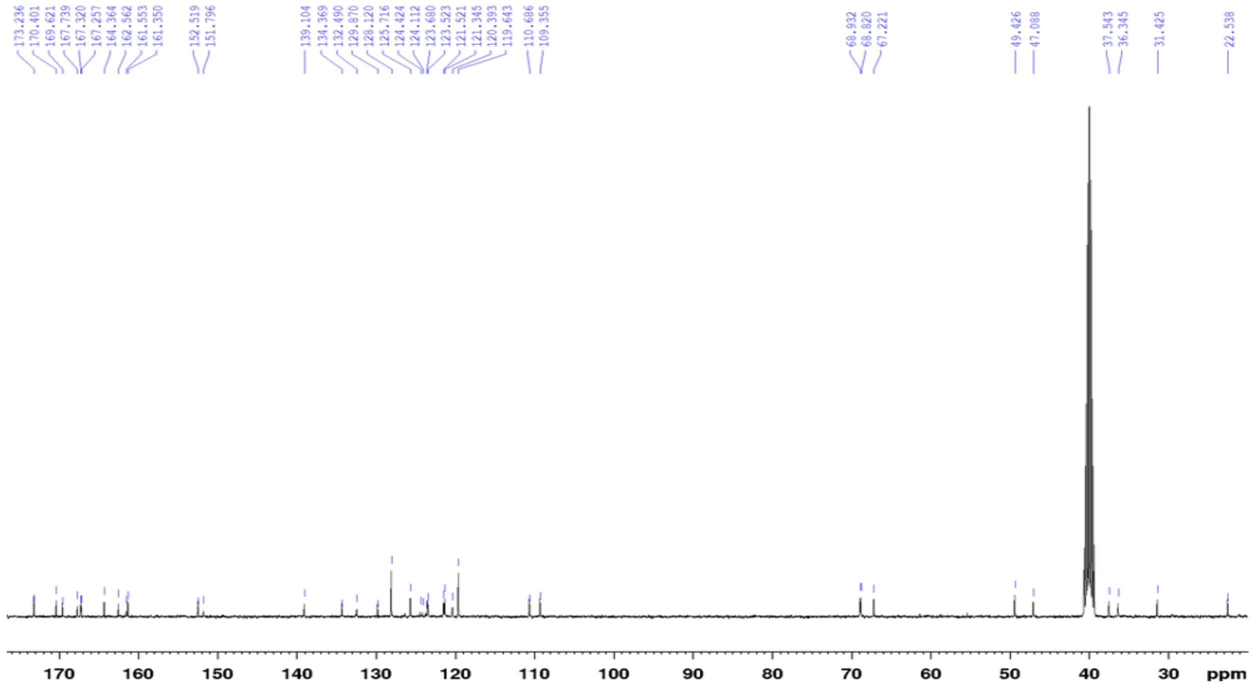

Figure S11 ^{1}H- and ^{13}C-NMR spectra of compound P4a

Compound P4b

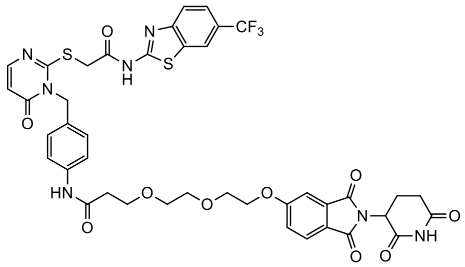

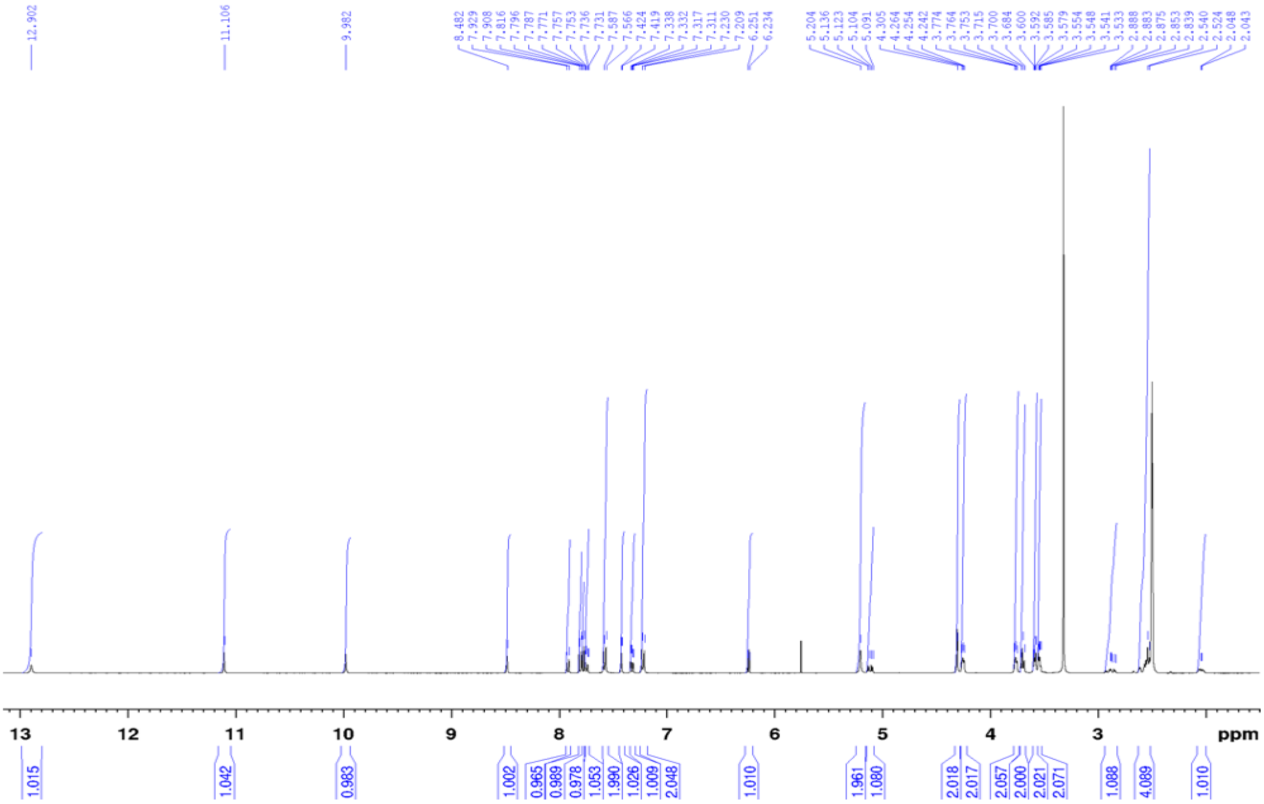

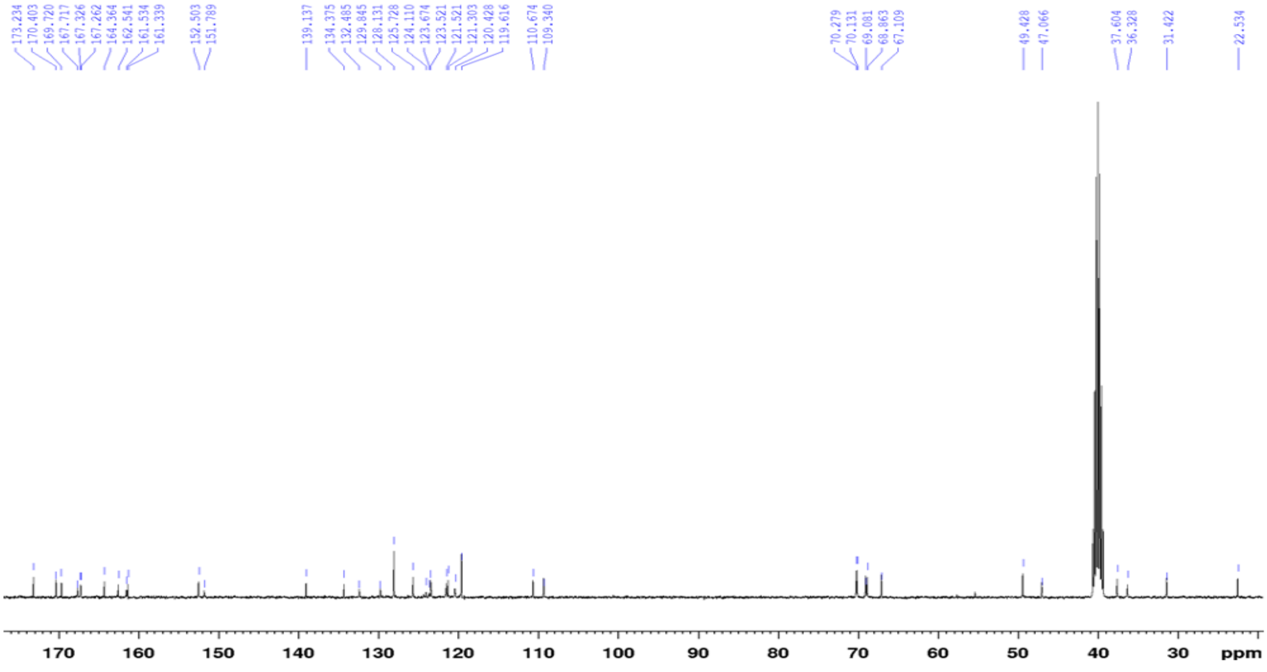

Figure S12 ^{1}H- and ^{13}C-NMR spectra of compound P4b

Compound P4c

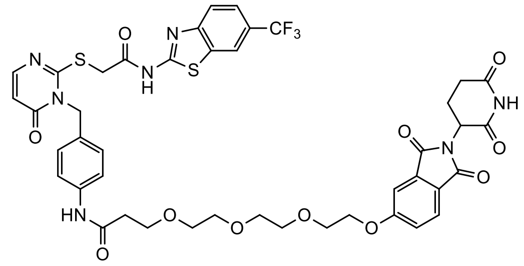

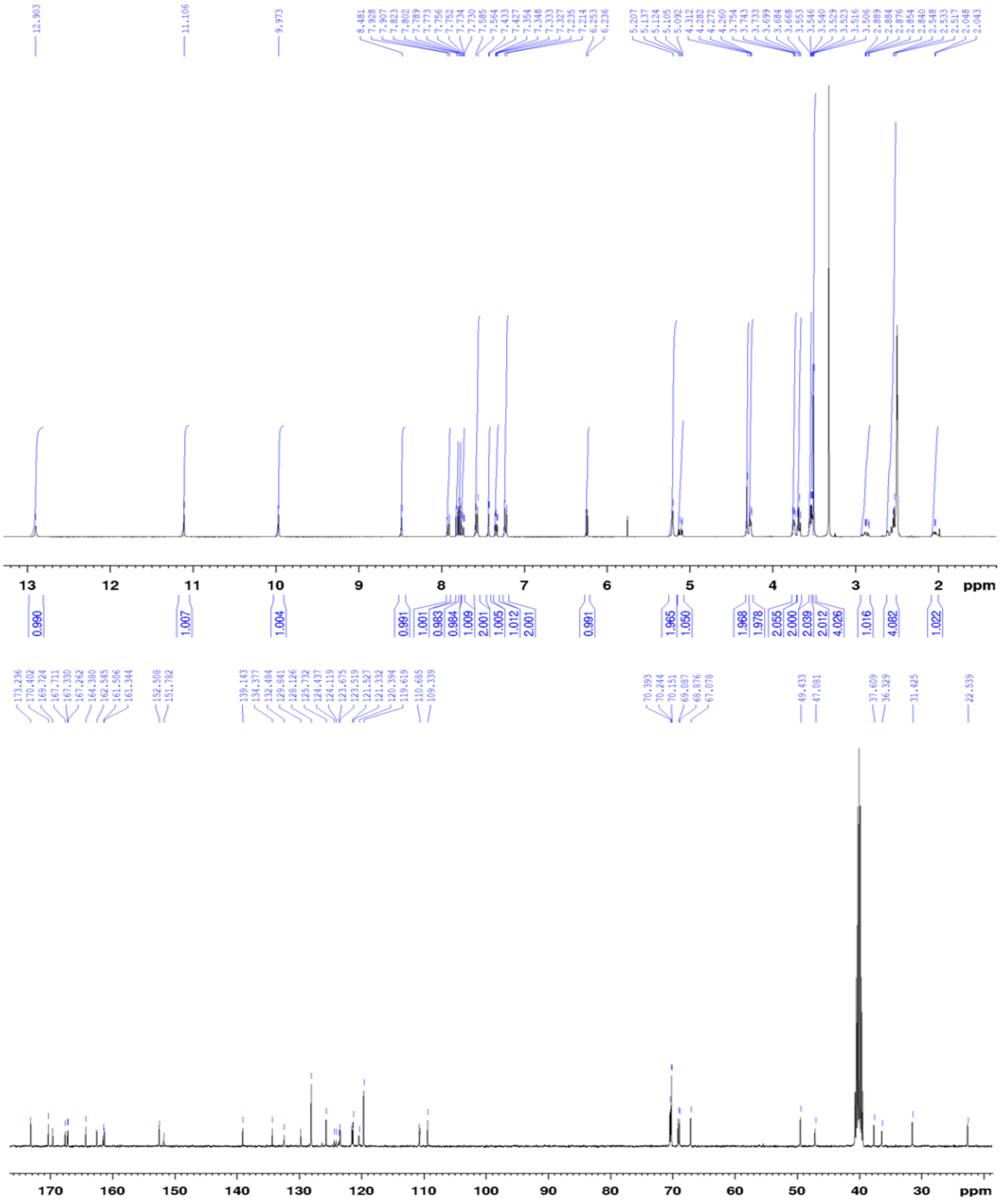

Figure S13 ^{1}H- and ^{13}C-NMR spectra of compound P4c

Compound P4d

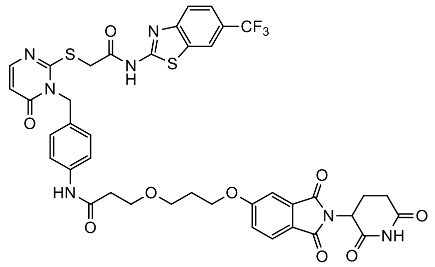

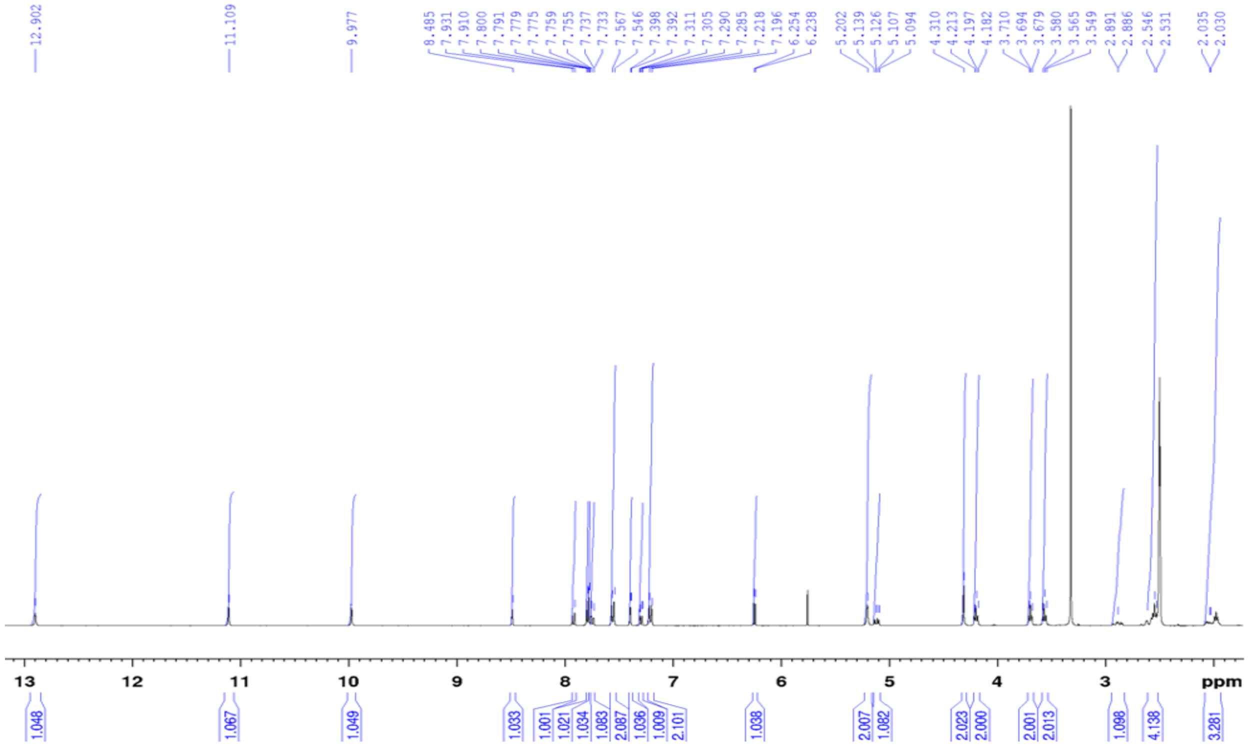

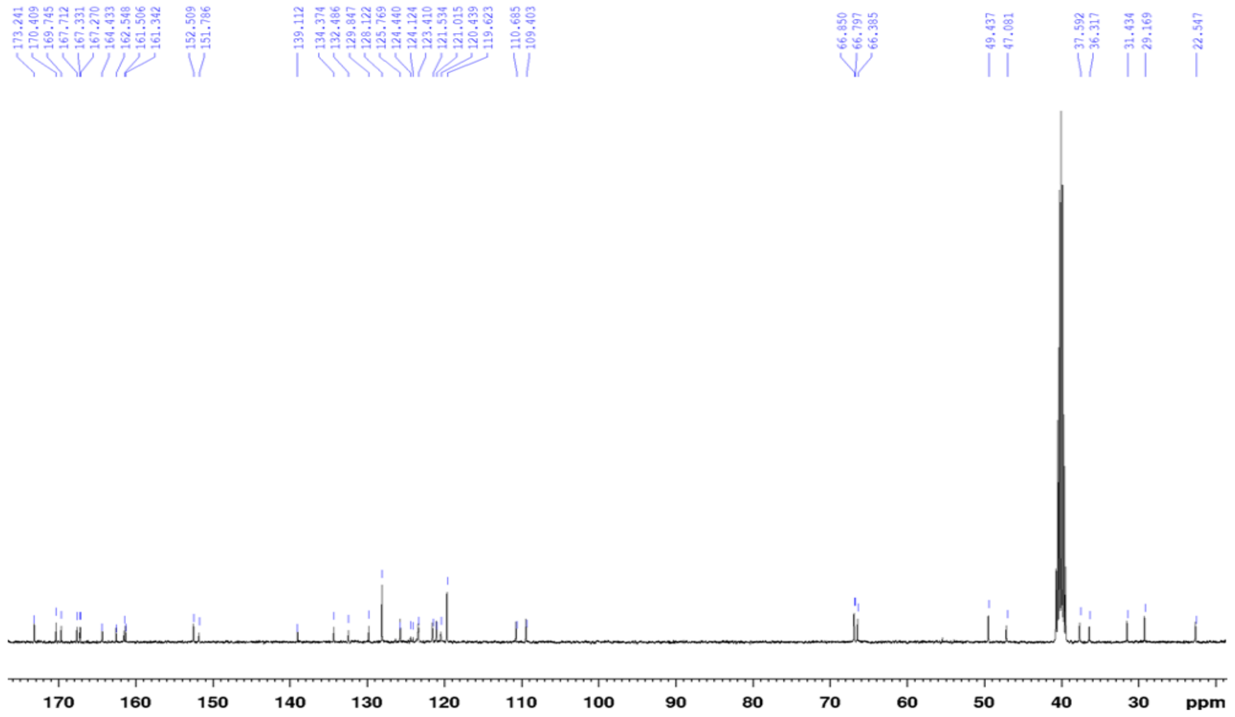

Figure S14 ^{1}H- and ^{13}C-NMR spectra of compound P4d

HPLC chromatograms and MS spectra of synthesized PROTAC compounds

Compound P1a

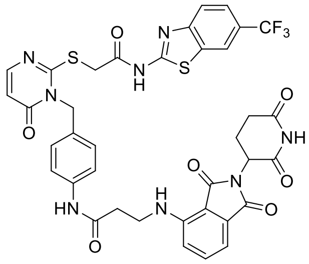

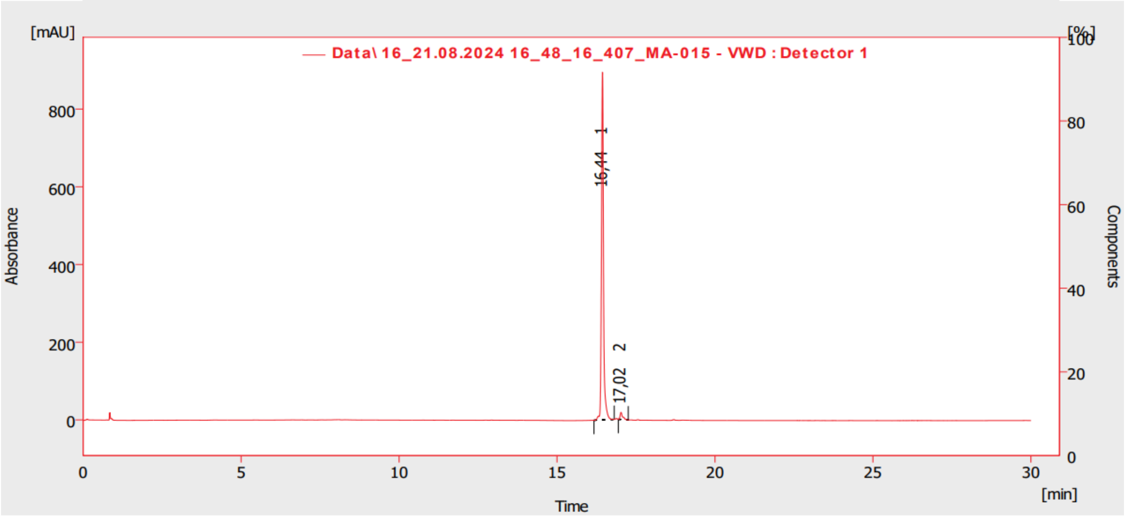

Result Table (Uncal - Data\16_21.08.2024 16_48_16_407_MA-015 - VWD : Detector 1)

	Reten. Time [min]	Area [mAU.s]	Height [mAU]	Area [%]	Height [%]	W05 [min]
1	16,437	4011,137	894,910	97,6	98,0	0,06
2	17,023	99,653	18,472	2,4	2,0	0,07
	Total	4110,790	913,382	100,0	100,0	

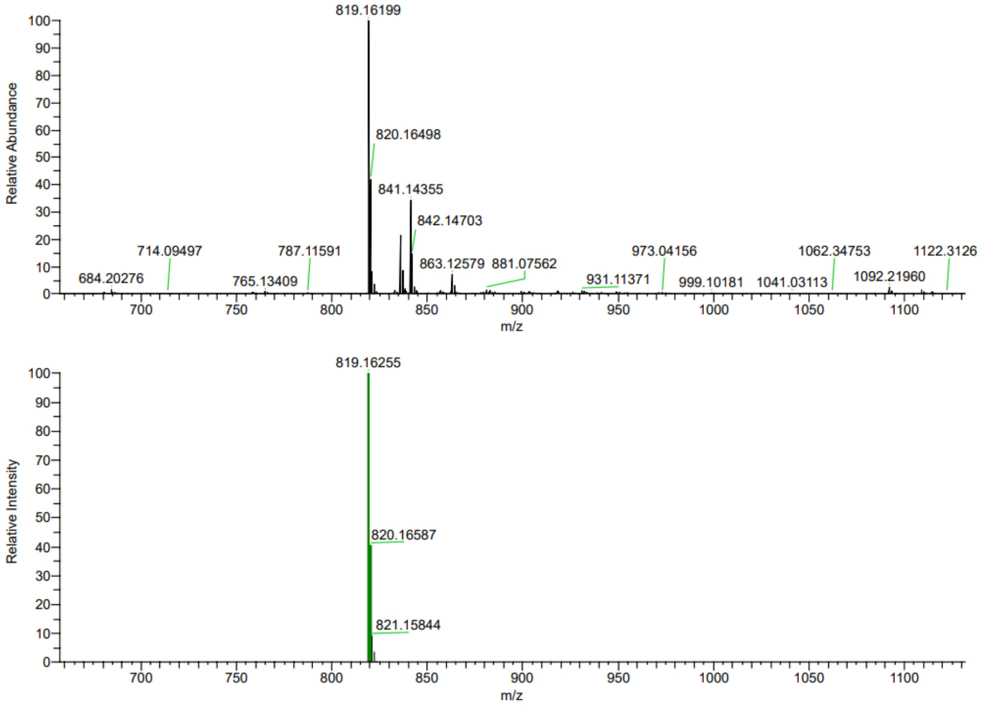

Figure S15 HPLC chromatogram and MS spectrum of compound P1a

Compound P1b

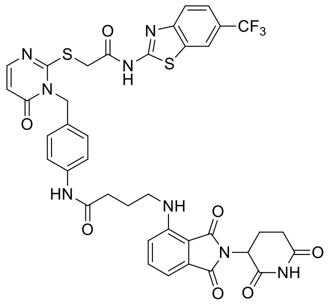

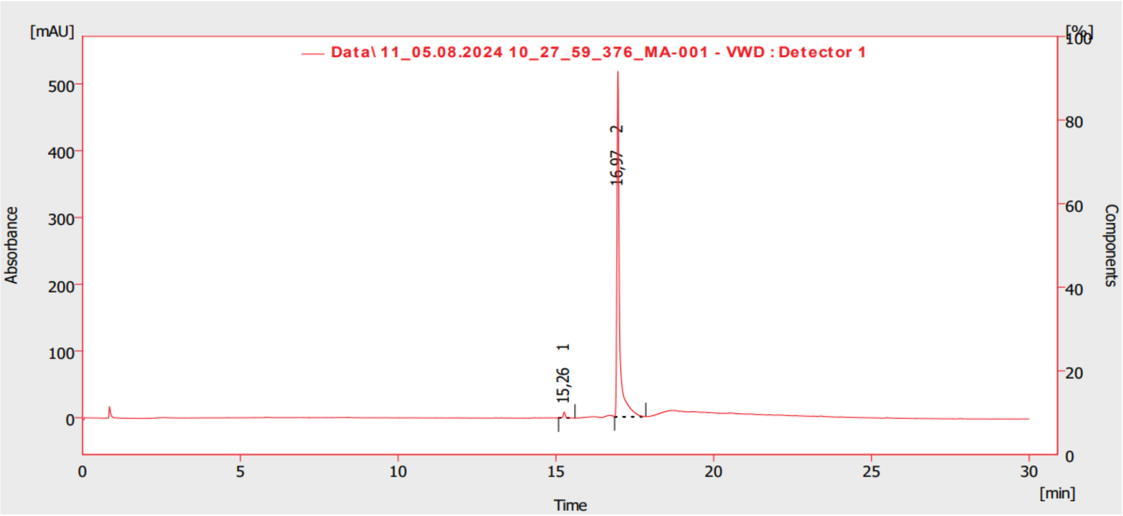

Result Table (Uncal - Data\11_05.08.2024 10_27_59_376_MA-001 - VWD : Detector 1)

	Reten. Time [min]	Area [mAU.s]	Height [mAU]	Area [%]	Height [%]	W05 [min]
1	15,264	44,438	8,811	1,5	1,7	0,07
2	16,966	2950,794	517,463	98,5	98,3	0,07
	Total	2995,231	526,274	100,0	100,0	

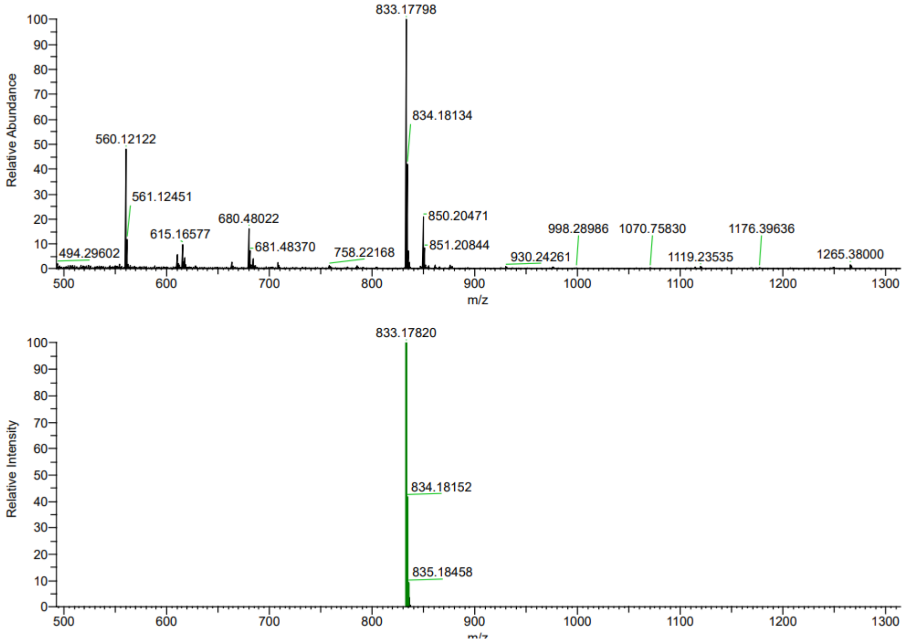

Figure S16 HPLC chromatogram and MS spectrum of compound P1b

Compound P1c

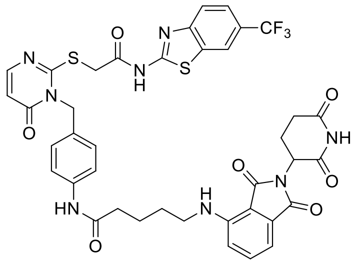

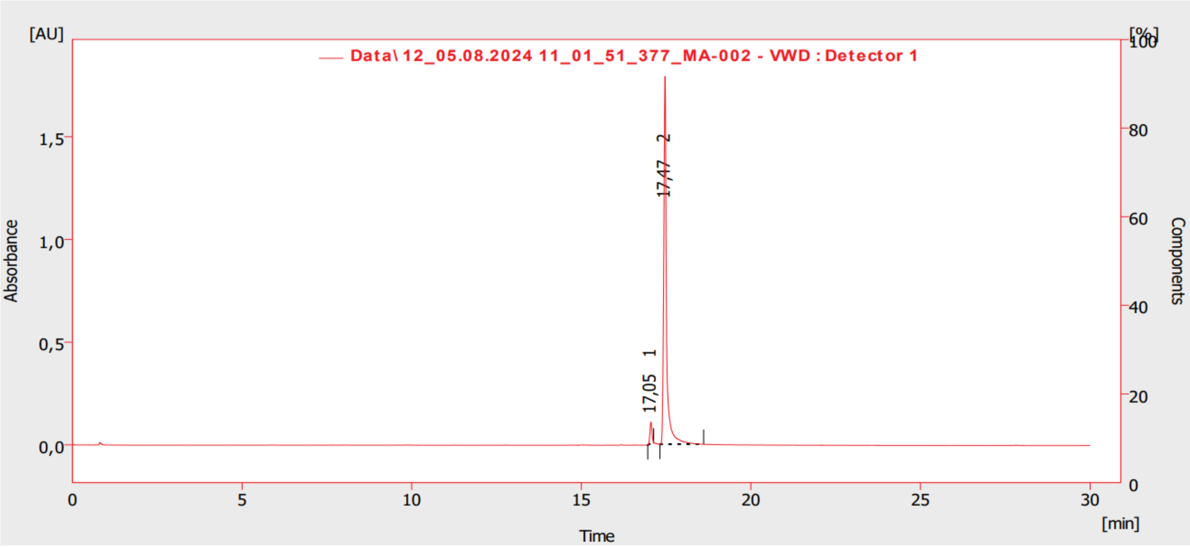

Result Table (Uncal - Data\12_05.08.2024 11_01_51_377_MA-002 - VWD : Detector 1)

	Reten. Time [min]	Area [mAU.s]	Height [mAU]	Area [%]	Height [%]	W05 [min]
1	17,052	437,173	105,974	4,1	5,6	0,07
2	17,470	10354,379	1790,879	95,9	94,4	0,07
	Total	10791,553	1896,853	100,0	100,0	

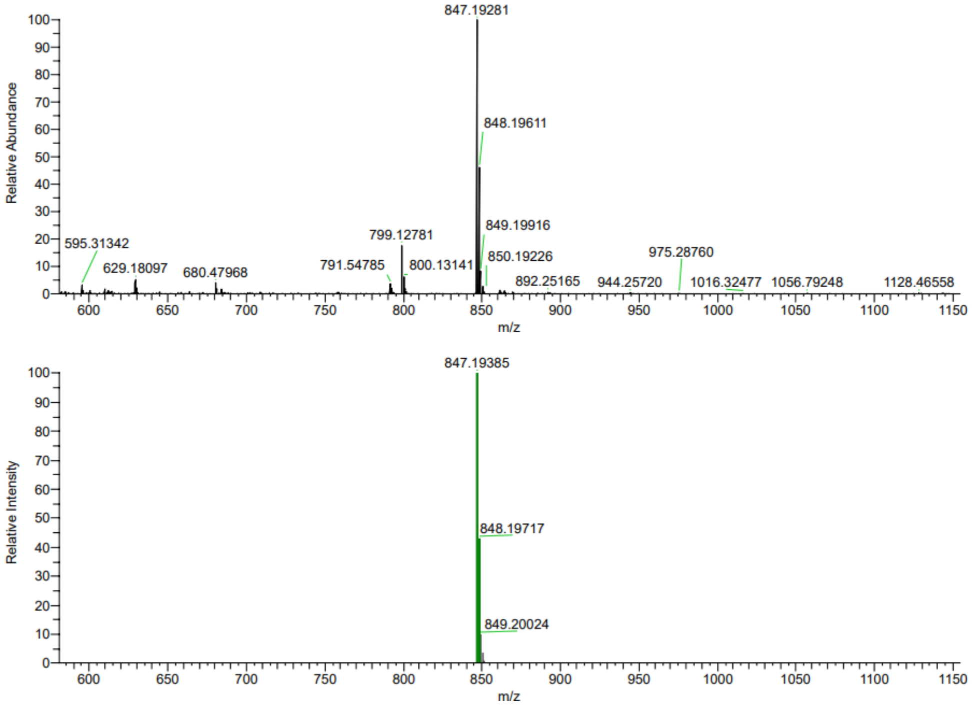

Figure S17 HPLC chromatogram and MS spectrum of compound P1c

Compound P1d

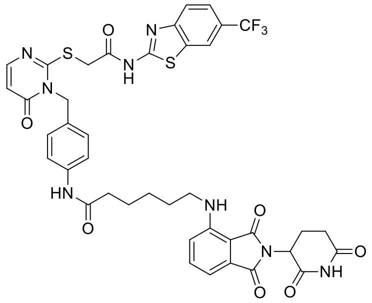

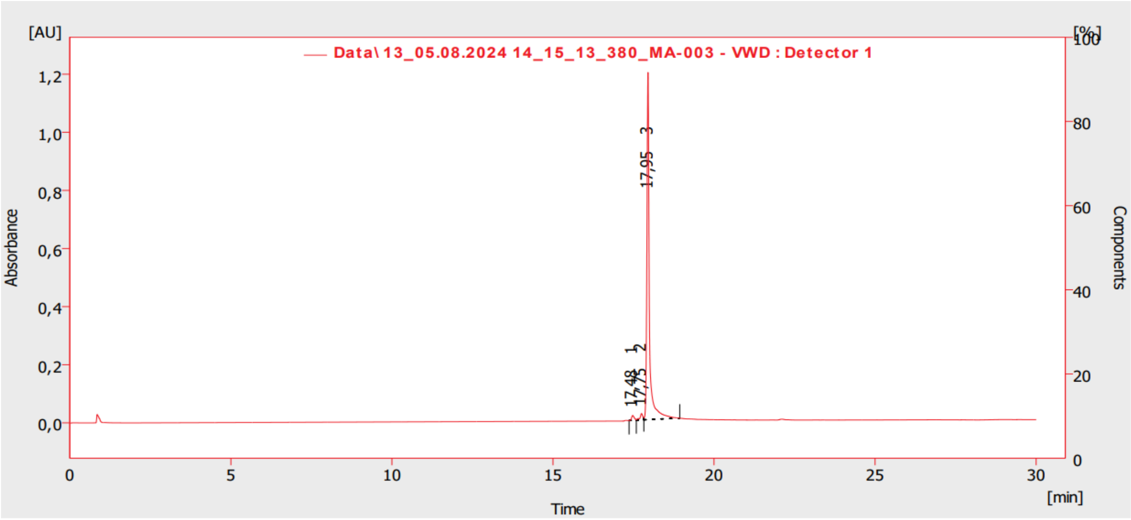

Result Table (Uncal - Data\13_05.08.2024 14_15_13_380_MA-003 - VWD : Detector 1)

	Reten. Time [min]	Area [mAU.s]	Height [mAU]	Area [%]	Height [%]	W05 [min]
1	17,476	93,851	16,730	1,4	1,4	0,09
2	17,750	132,844	21,894	2,0	1,8	0,08
3	17,950	6493,620	1194,914	96,6	96,9	0,07
	Total	6720,315	1233,538	100,0	100,0	

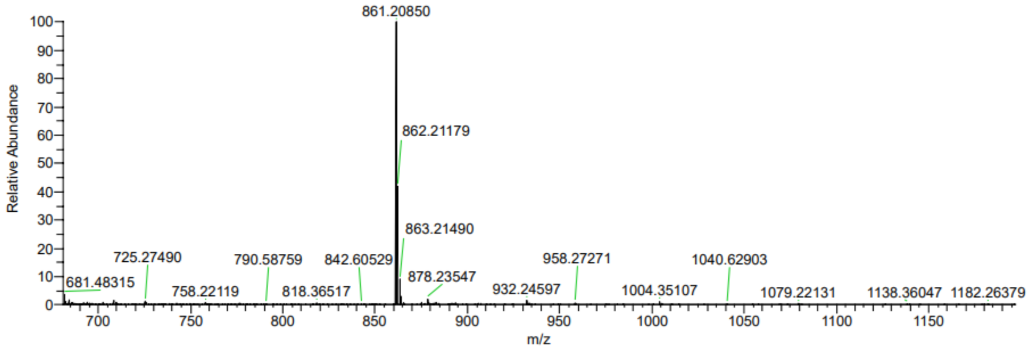

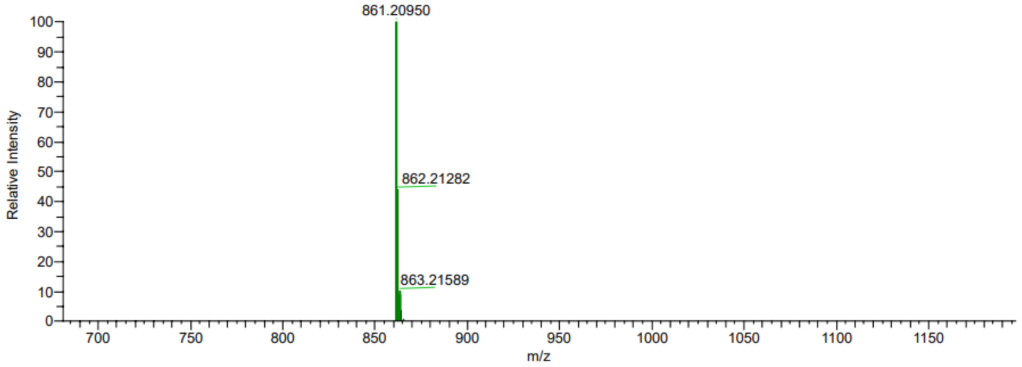

Figure S18 HPLC chromatogram and MS spectrum of compound P1d

Compound P2a

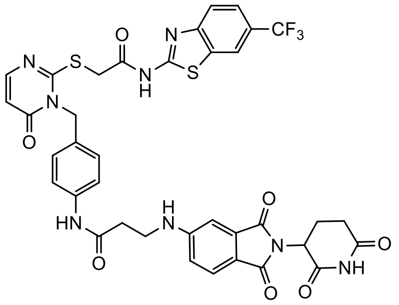

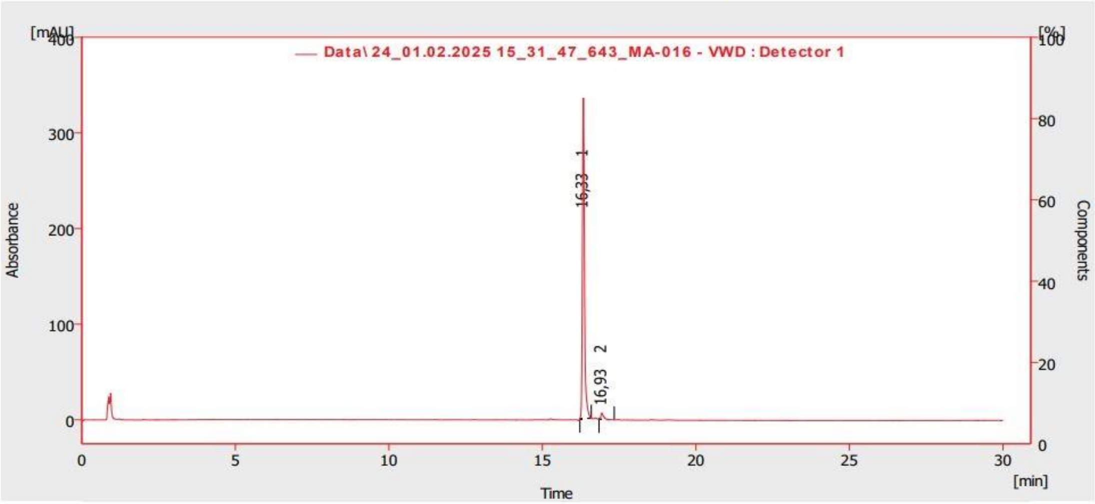

Result Table (Uncal - Data\24_01.02.2025 15_31_47_643_MA-016 - VWD : Detector 1)

	Reten. Time [min]	Area [mAU.s]	Height [mAU]	Area [%]	Height [%]	W05 [min]
1	16,334	1554,830	335,507	97,5	98,1	0,07
2	16,935	39,195	6,619	2,5	1,9	0,08
	Total	1594,025	342,125	100,0	100,0	

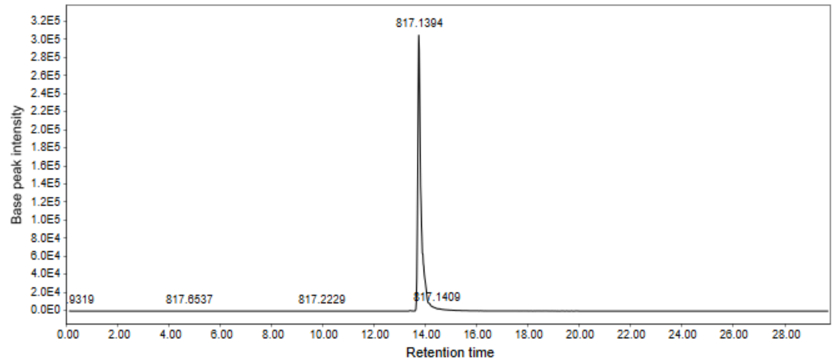

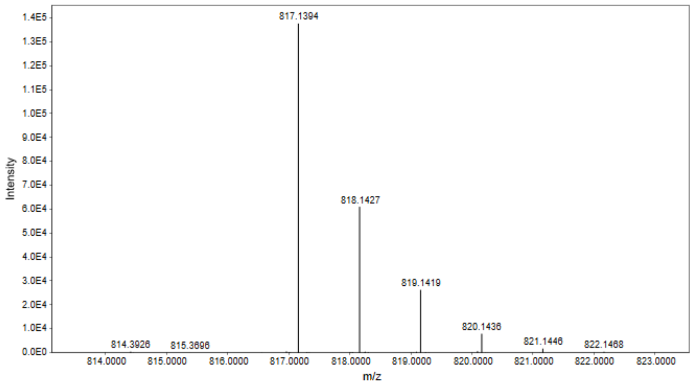

Figure S19 HPLC chromatogram and MS spectrum of compound P2a

Compound P2d

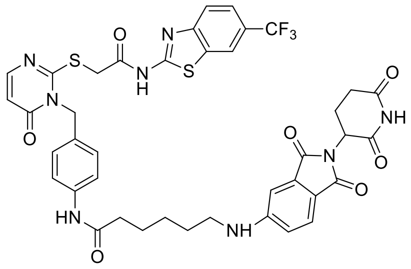

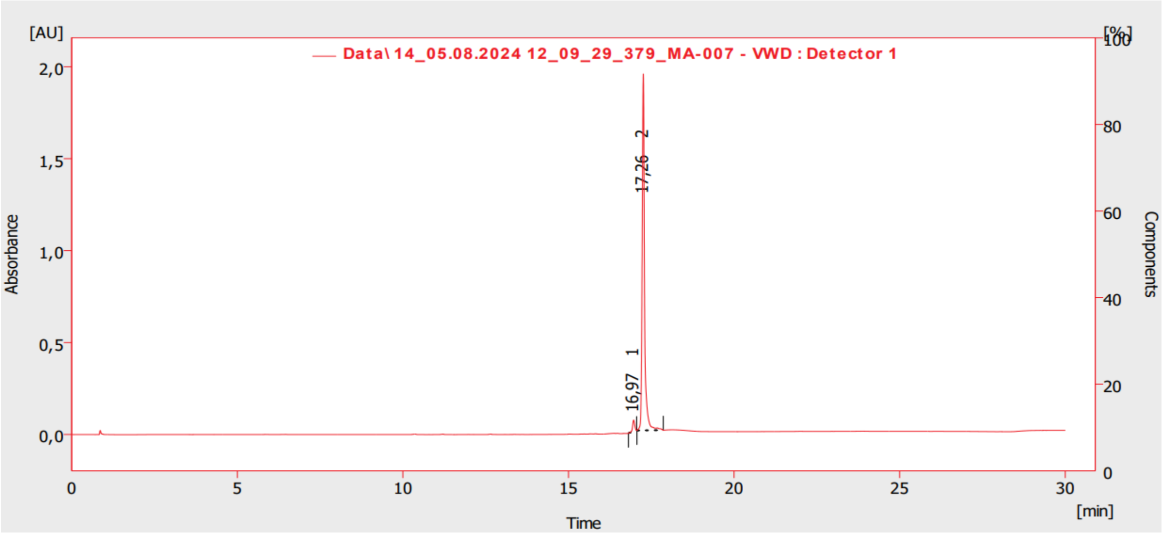

Result Table (Uncal - Data\14_05.08.2024 12_09_29_379_MA-007 - VWD : Detector 1)

	Reten. Time [min]	Area [mAU.s]	Height [mAU]	Area [%]	Height [%]	W05 [min]
1	16,967	280,164	62,336	2,8	3,1	0,07
2	17,260	9619,508	1938,442	97,2	96,9	0,07
	Total	9899,672	2000,778	100,0	100,0	

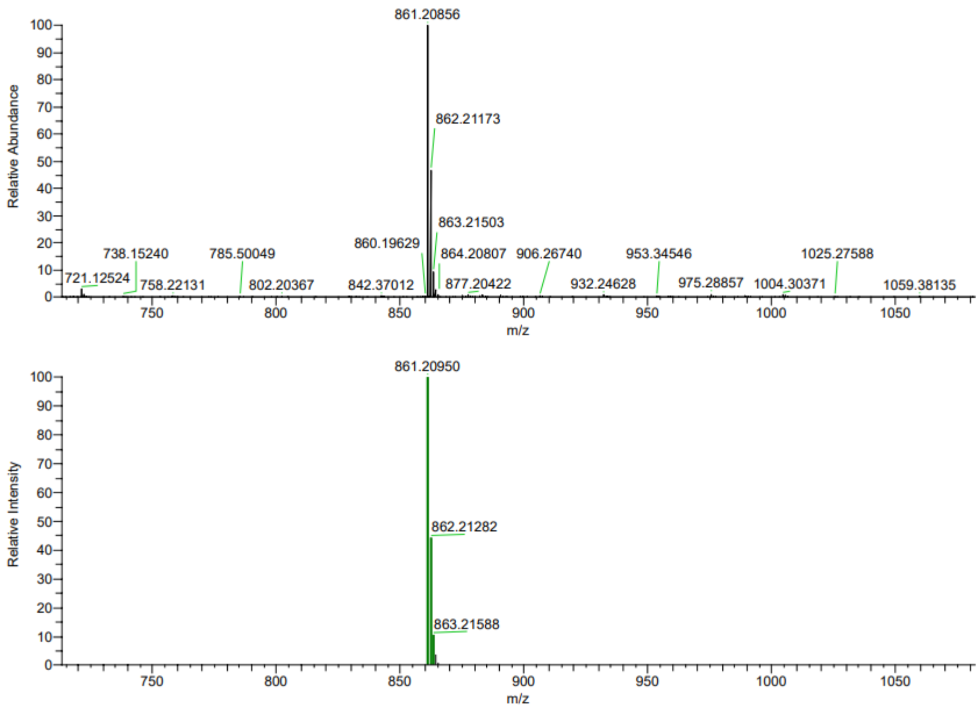

Figure S20 HPLC chromatogram and MS spectrum of compound P2d

Compound P3a

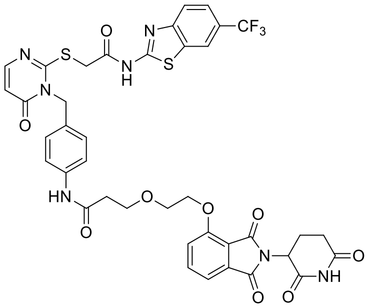

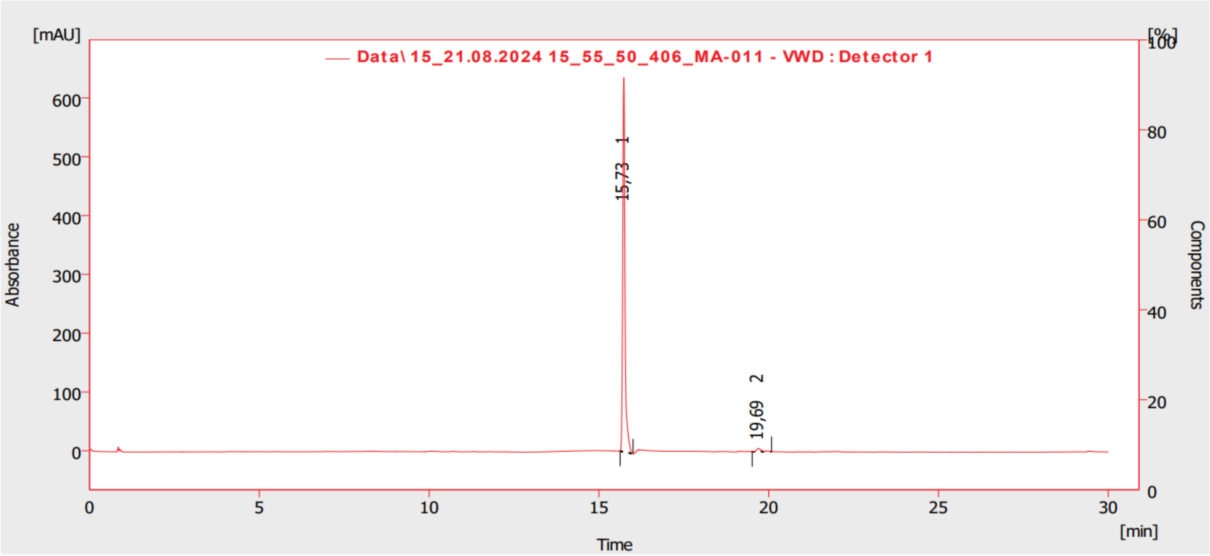

Result Table (Uncal - Data\15_21.08.2024 15_55_50_406_MA-011 - VWD : Detector 1)

	Reten. Time [min]	Area [mAU.s]	Height [mAU]	Area [%]	Height [%]	W05 [min]
1	15,731	2908,443	636,532	98,2	99,2	0,07
2	19,692	52,889	5,401	1,8	0,8	0,14
	Total	2961,333	641,933	100,0	100,0	

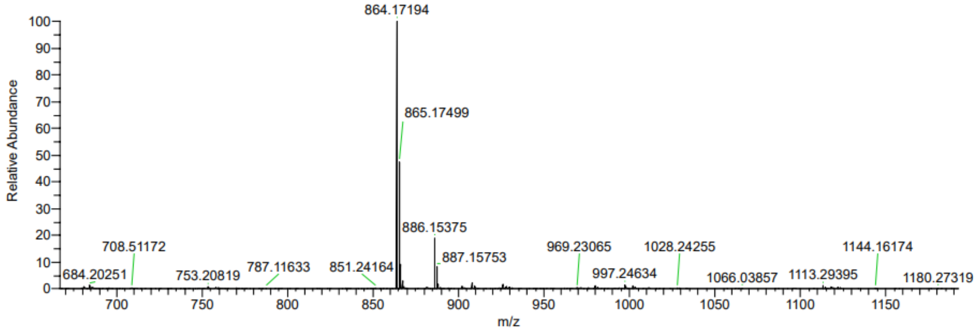

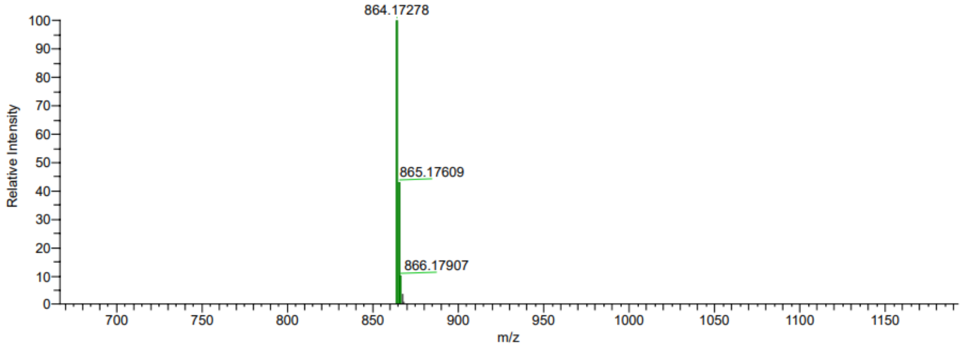

Figure S21 HPLC chromatogram and MS spectrum of compound P3a

Compound P3b

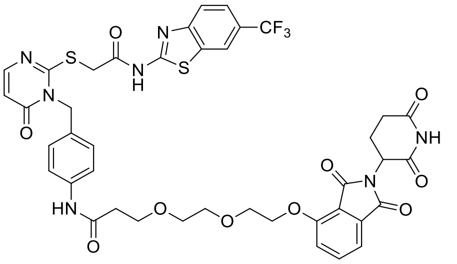

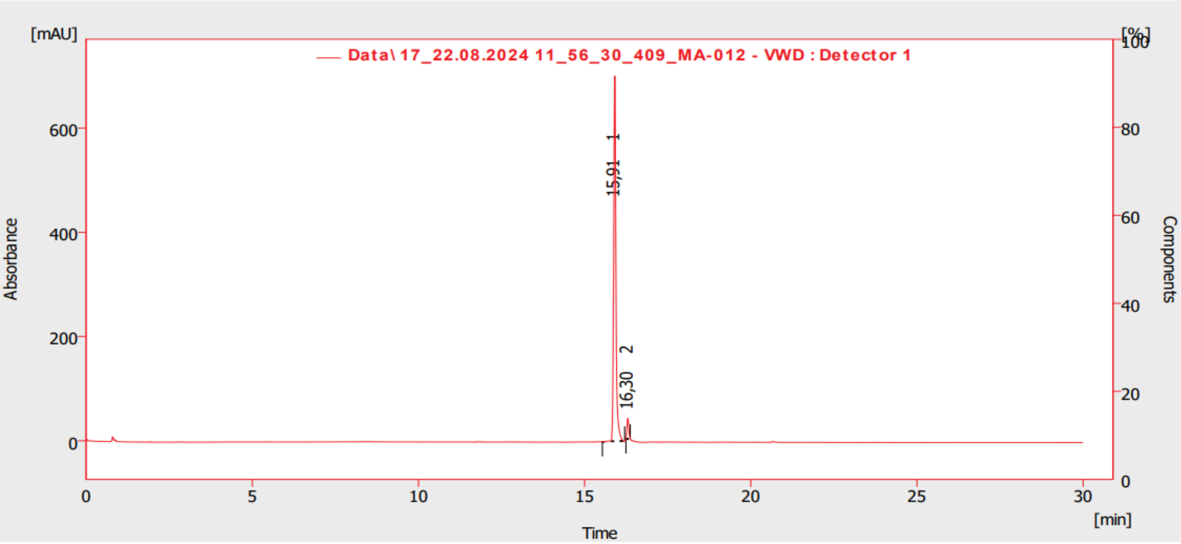

Result Table (Uncal - Data\17_22.08.2024 11_56_30_409_MA-012 - VWD : Detector 1)

	Reten. Time [min]	Area [mAU.s]	Height [mAU]	Area [%]	Height [%]	W05 [min]
1	15,907	3298,140	700,910	95,7	94,7	0,07
2	16,300	149,551	39,401	4,3	5,3	0,06
	Total	3447,691	740,311	100,0	100,0	

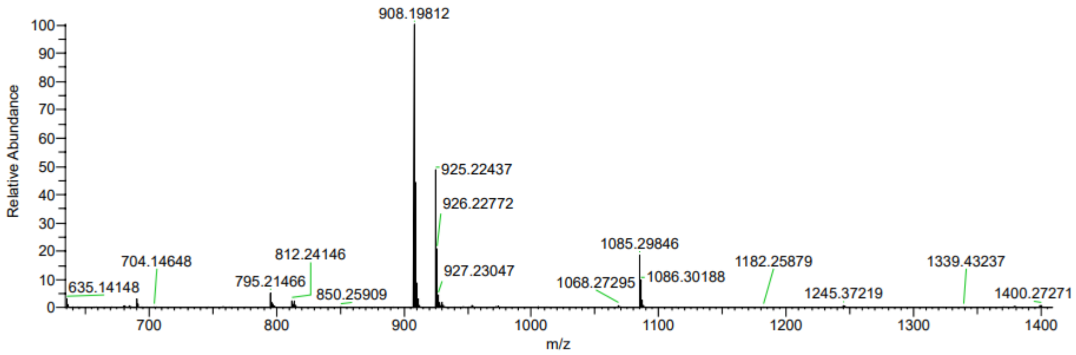

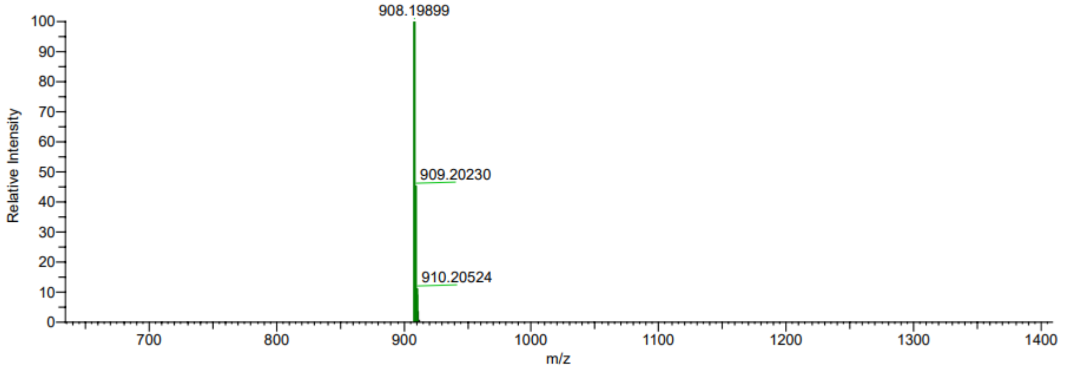

Figure S22 HPLC chromatogram and MS spectrum of compound P3b

Compound P3c

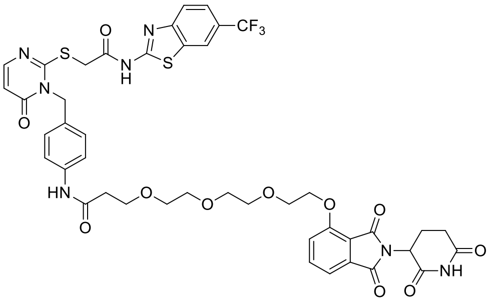

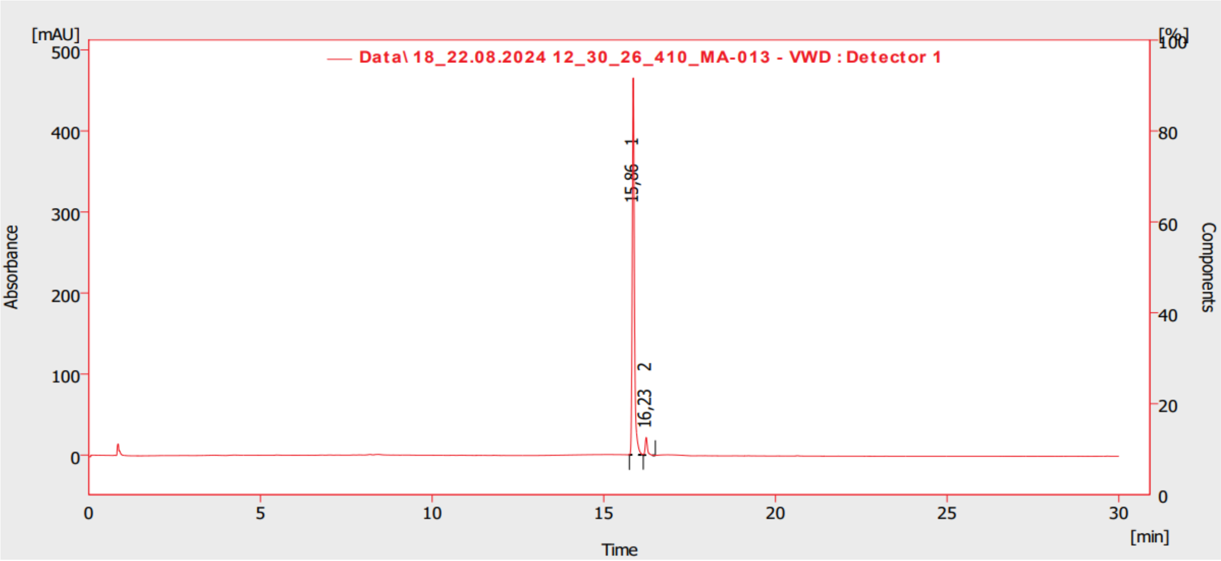

Result Table (Uncal - Data\18_22.08.2024 12_30_26_410_MA-013 - VWD : Detector 1)

	Reten. Time [min]	Area [mAU.s]	Height [mAU]	Area [%]	Height [%]	W05 [min]
1	15,859	2050,142	465,147	95,4	95,5	0,07
2	16,235	98,950	21,933	4,6	4,5	0,07
	Total	2149,092	487,080	100,0	100,0	

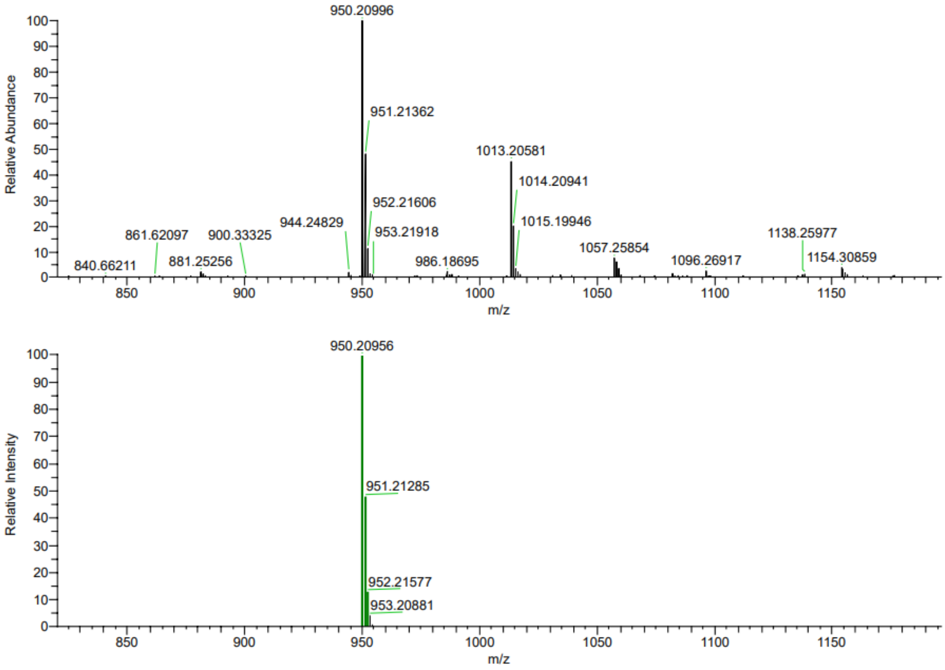

Figure S23 HPLC chromatogram and MS spectrum of compound P3c

Compound P3d

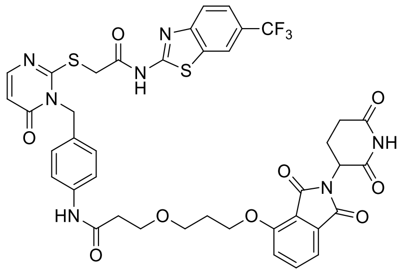

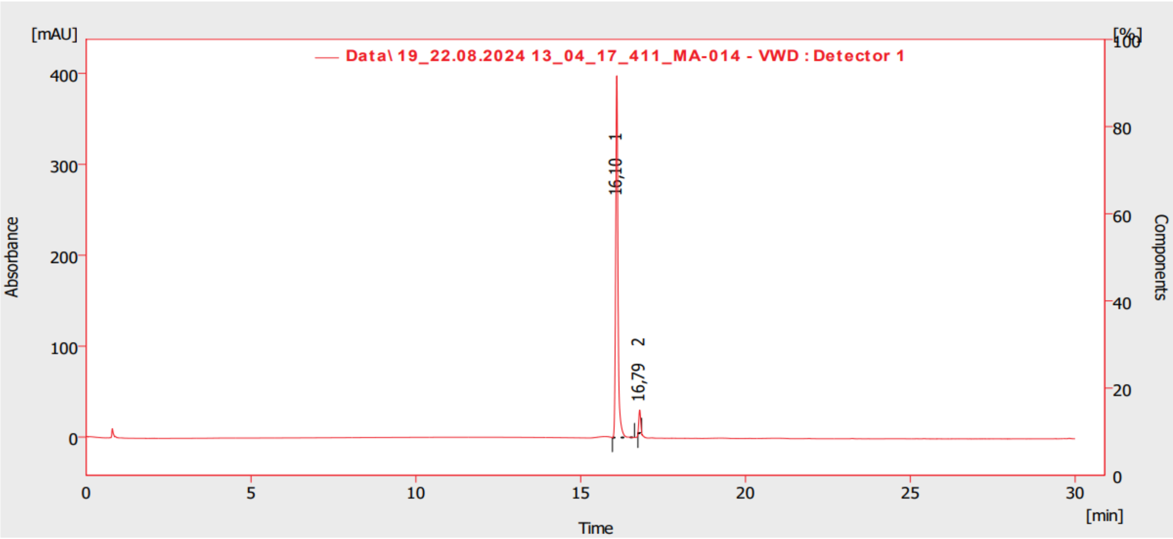

Result Table (Uncal - Data\19_22.08.2024 13_04_17_411_MA-014 - VWD : Detector 1)

	Reten. Time [min]	Area [mAU.s]	Height [mAU]	Area [%]	Height [%]	W05 [min]
1	16,097	1923,304	397,995	95,4	94,0	0,07
2	16,790	92,458	25,194	4,6	6,0	0,06
	Total	2015,762	423,188	100,0	100,0	

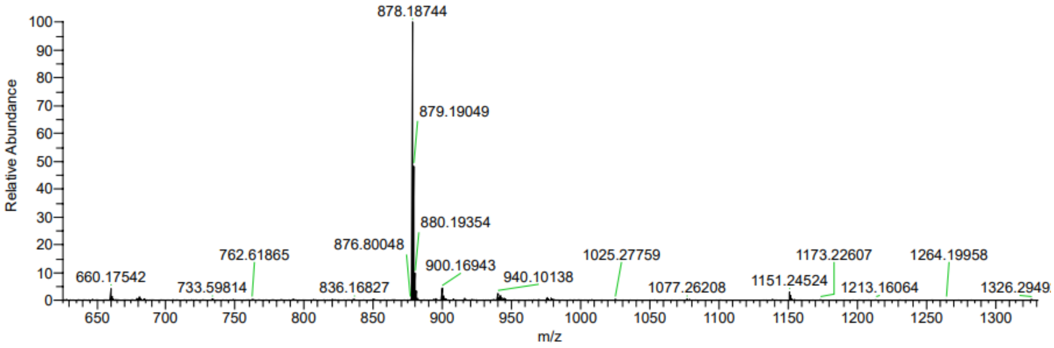

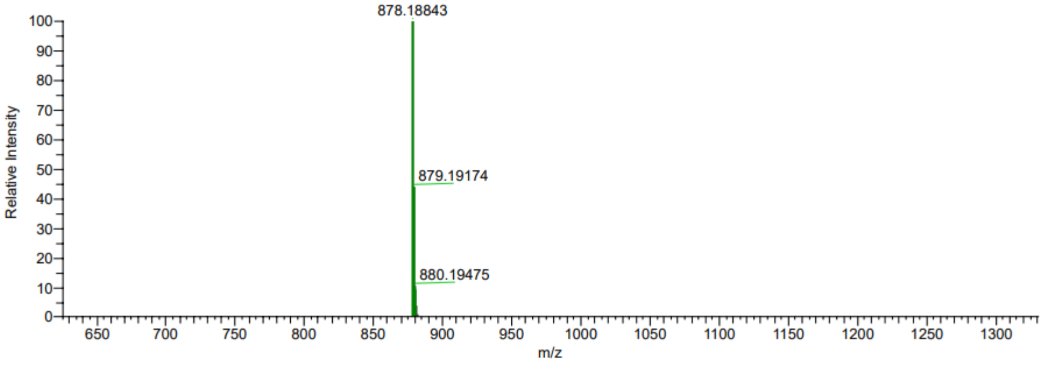

Figure S24 HPLC chromatogram and MS spectrum of compound P3d

Compound P4a

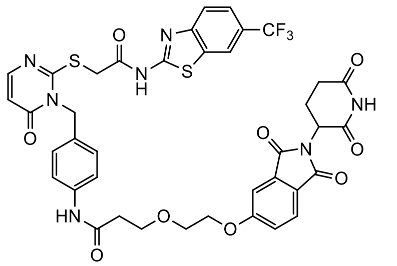

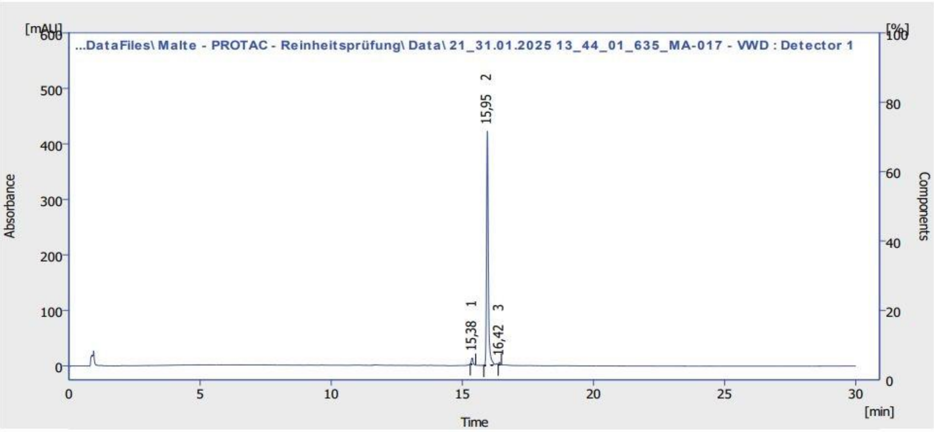

Result Table (Uncal - D:\Clarity\DataFiles\Malte - PROTAC - Reinheitsprüfung\Data\21_31.01.2025 13_44_01_635_MA-017 - VV

	Reten. Time [min]	Area [mAU.s]	Height [mAU]	Area [%]	Height [%]	W05 [min]
1	15,376	48,221	11,565	2,4	2,6	0,07
2	15,953	1973,524	422,055	96,7	96,3	0,07
3	16,419	19,167	4,784	0,9	1,1	0,07
	Total	2040,912	438,404	100,0	100,0	

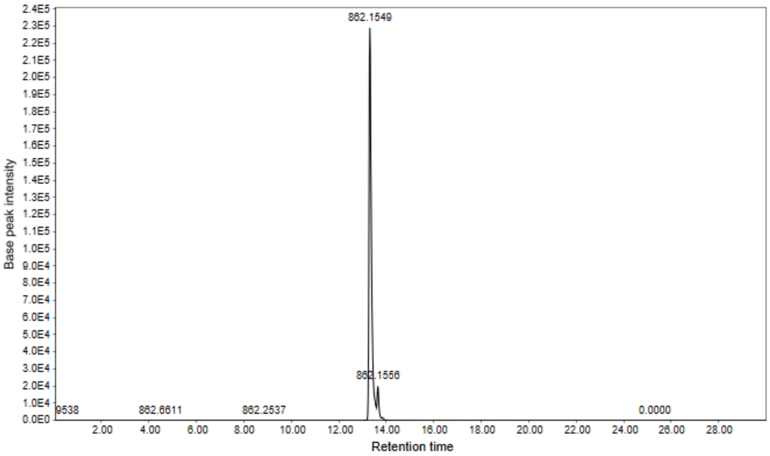

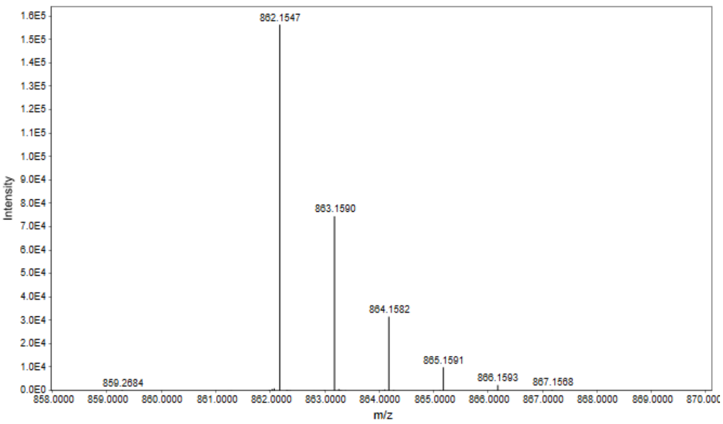

Figure S25 HPLC chromatogram and MS spectrum of compound P4a

Compound P4b

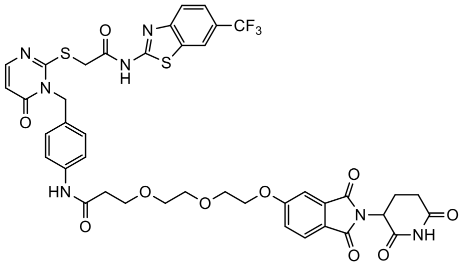

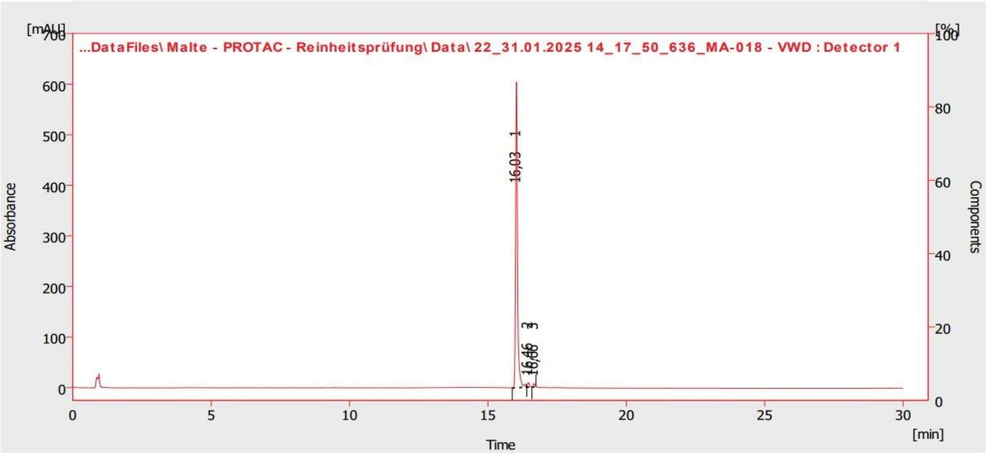

Result Table (Uncal - D:\Clarity\DataFiles\Malte - PROTAC - Reinheitsprüfung\Data\22_31.01.2025 14_17_50_636_MA-018 - VV

	Reten. Time [min]	Area [mAU.s]	Height [mAU]	Area [%]	Height [%]	W05 [min]
1	16,030	2879,713	603,934	97,4	97,4	0,07
2	16,462	49,369	9,171	1,7	1,5	0,10
3	16,662	26,187	6,647	0,9	1,1	0,06
	Total	2955,269	619,752	100,0	100,0	

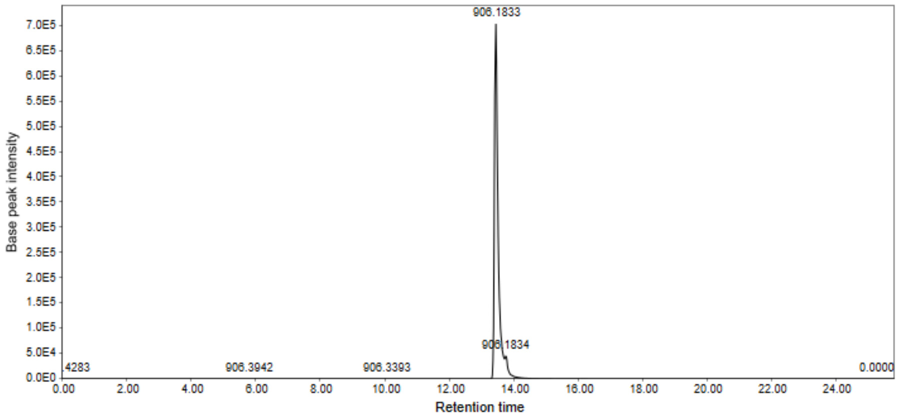

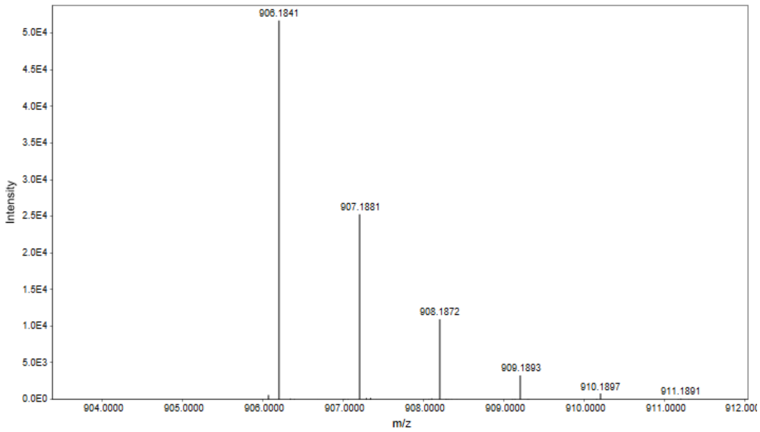

Figure S26 HPLC chromatogram and MS spectrum of compound P4b

Compound P4c

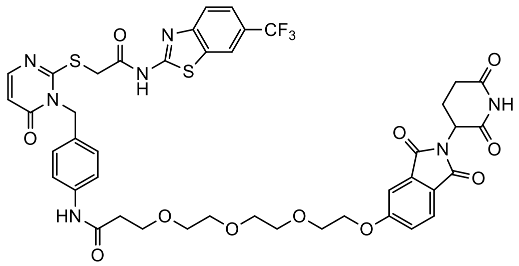

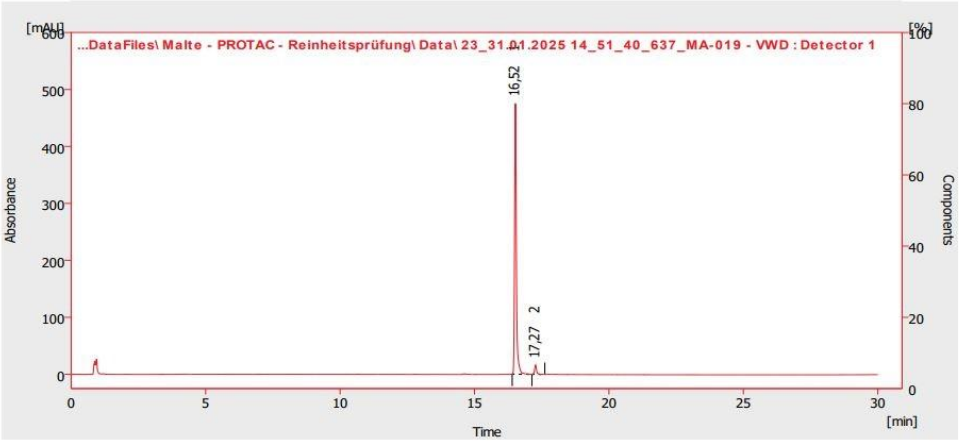

Result Table (Uncal - D:\Clarity\DataFiles\Malte - PROTAC - Reinheitsprüfung\Data\23_31.01.2025 14_51_40_637_MA-019 - VV

	Reten. Time [min]	Area [mAU.s]	Height [mAU]	Area [%]	Height [%]	W05 [min]
1	16,521	2209,949	475,208	96,4	96,6	0,07
2	17,268	83,546	16,763	3,6	3,4	0,07
	Total	2293,496	491,972	100,0	100,0	

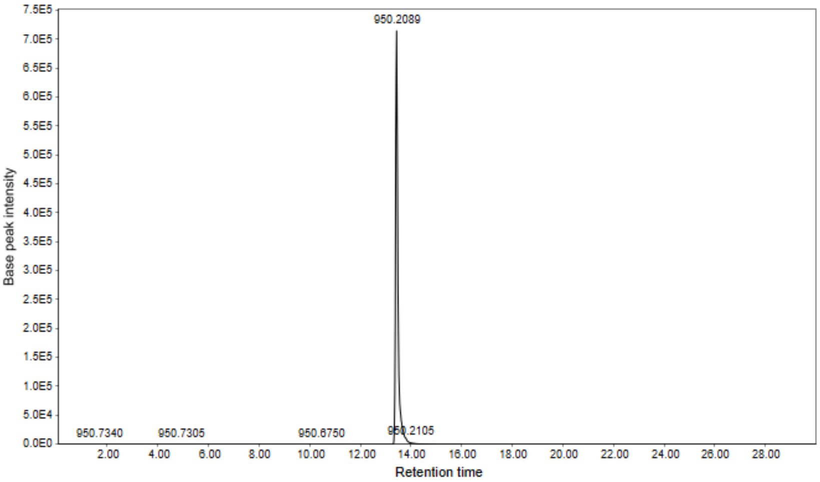

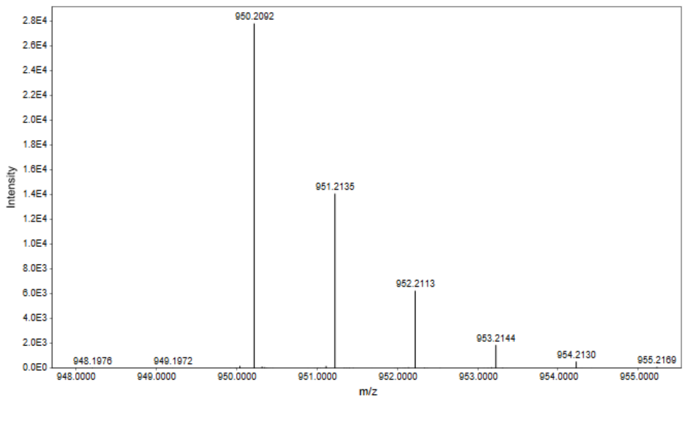

Figure S27 HPLC chromatogram and MS spectrum of compound P4c

Compound P4d

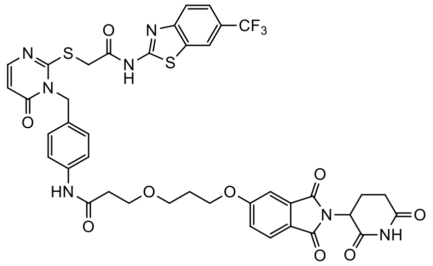

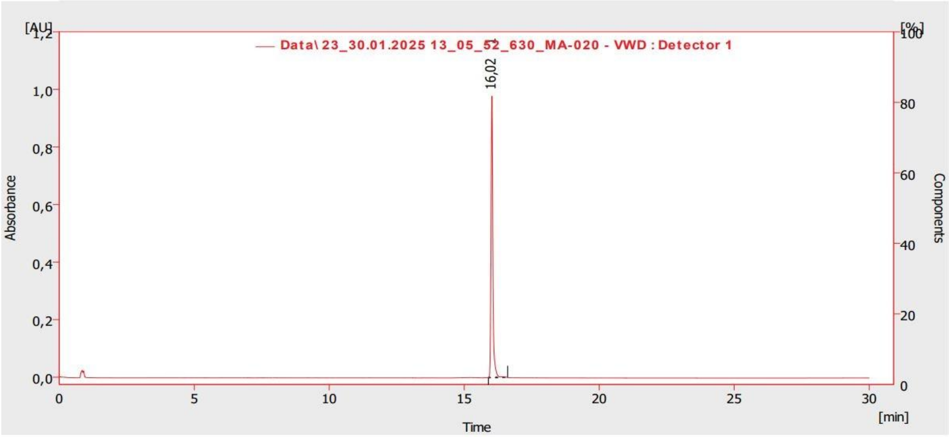

Result Table (Uncal - Data\23_30.01.2025 13_05_52_630_MA-020 - VWD : Detector 1)

	Reten. Time [min]	Area [mAU.s]	Height [mAU]	Area [%]	Height [%]	W05 [min]
1	16,023	4611,278	978,170	100,0	100,0	0,07
	Total	4611,278	978,170	100,0	100,0	

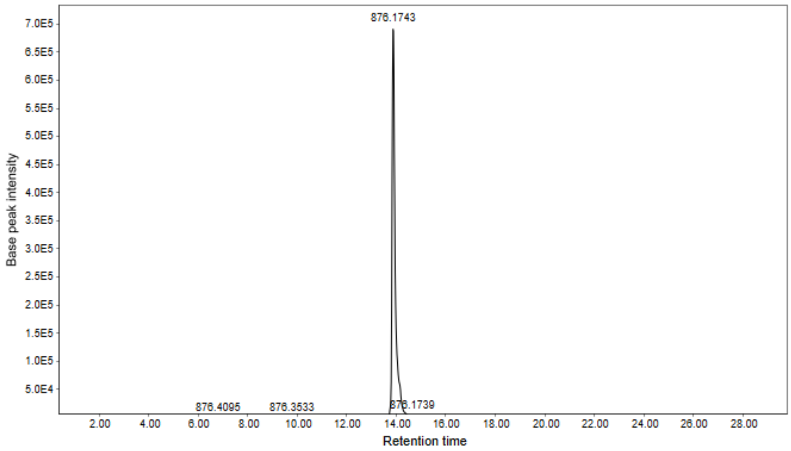

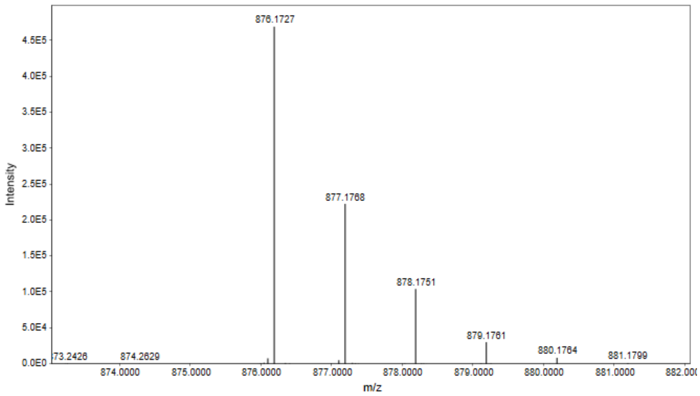

Figure S28 HPLC chromatogram and MS spectrum of compound P4d
